# Supplementary material for: Association of Metabolomic Biomarkers with Sleeve Gastrectomy Weight Loss Outcomes
Source: Metabolites. 2023 Mar 31;13(4):506. doi: 10.3390/metabo13040506 (PMC10145663; doi:10.3390/metabo13040506)

**Figure S2:** Fecal metabolite predictive models and ROC plots for three-month post-SG weight loss outcomes: Tertile 1 versus all patients at baseline


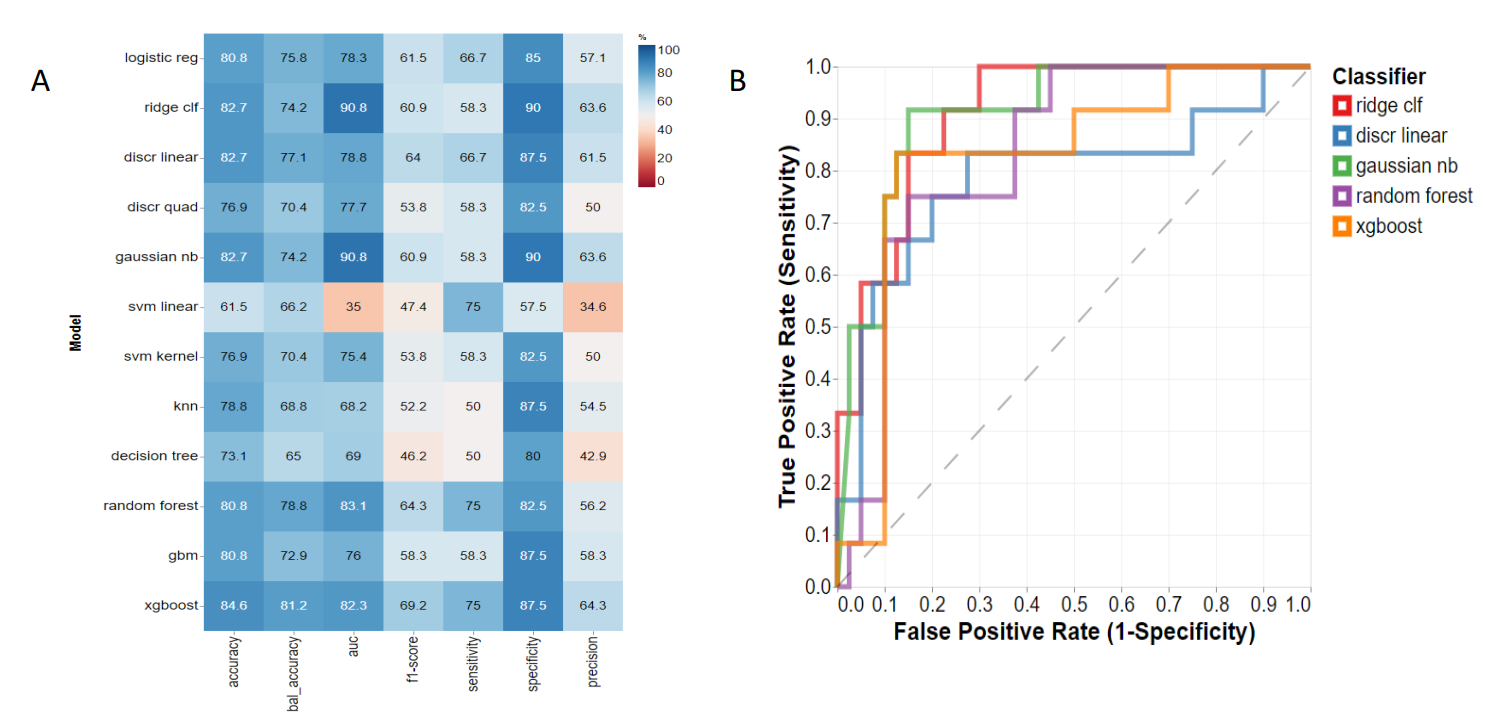

Supplement: Supplementary file 1 [file metabolites-13-00506-s001.zip › Supplementary Figure 2.docx]
