# Supplementary material for: Association of Metabolomic Biomarkers with Sleeve Gastrectomy Weight Loss Outcomes
Source: Metabolites. 2023 Mar 31;13(4):506. doi: 10.3390/metabo13040506 (PMC10145663; doi:10.3390/metabo13040506)
Supplement: Supplementary file 1 [file metabolites-13-00506-s001.zip › Supplementary Figure 3.docx]

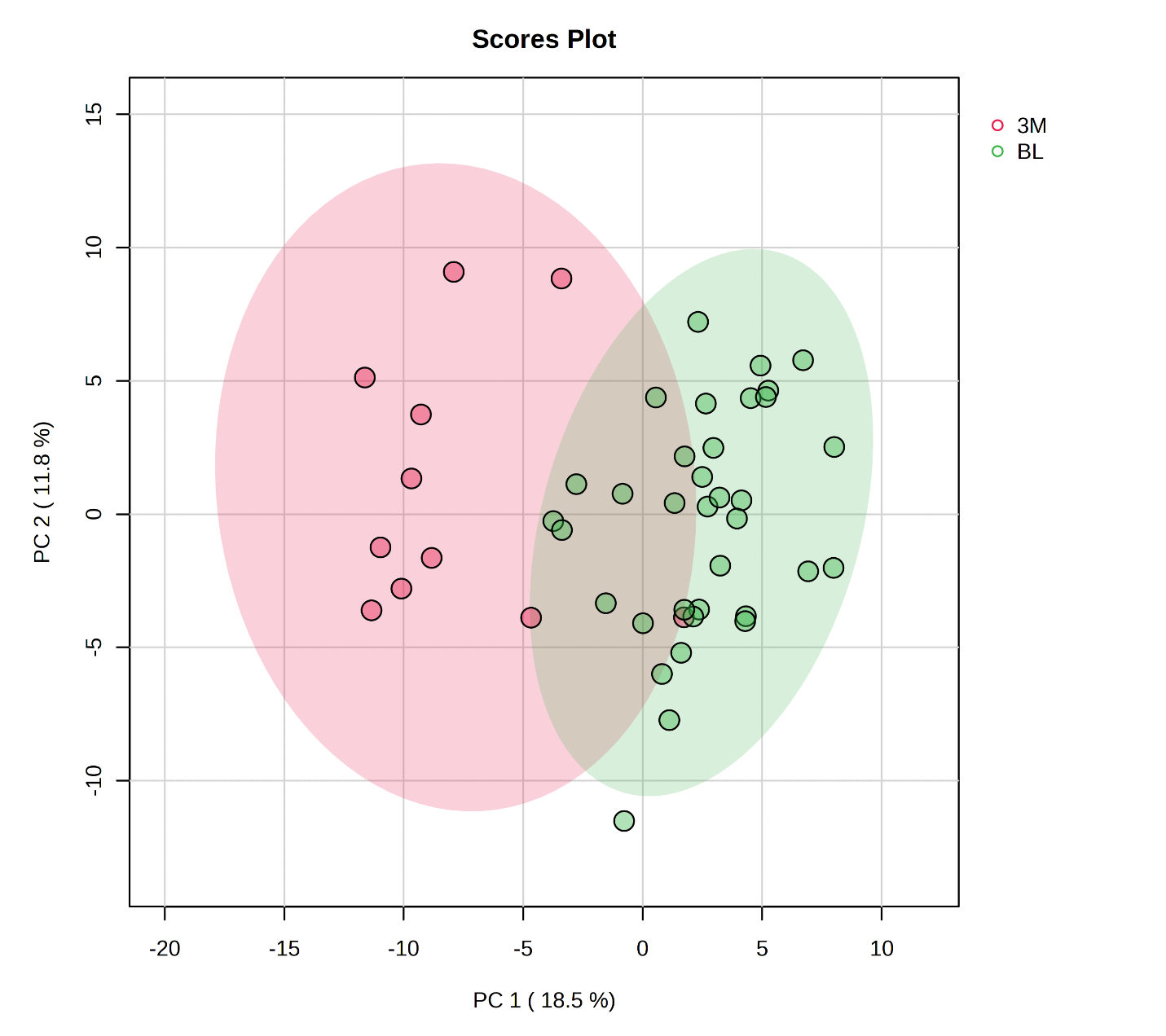


**Supplementary Figure S3A**: Serum Data, PCA scores of only females of Tertile 3 at three months post-sleeve gastrectomy compared with only female patients at baseline.


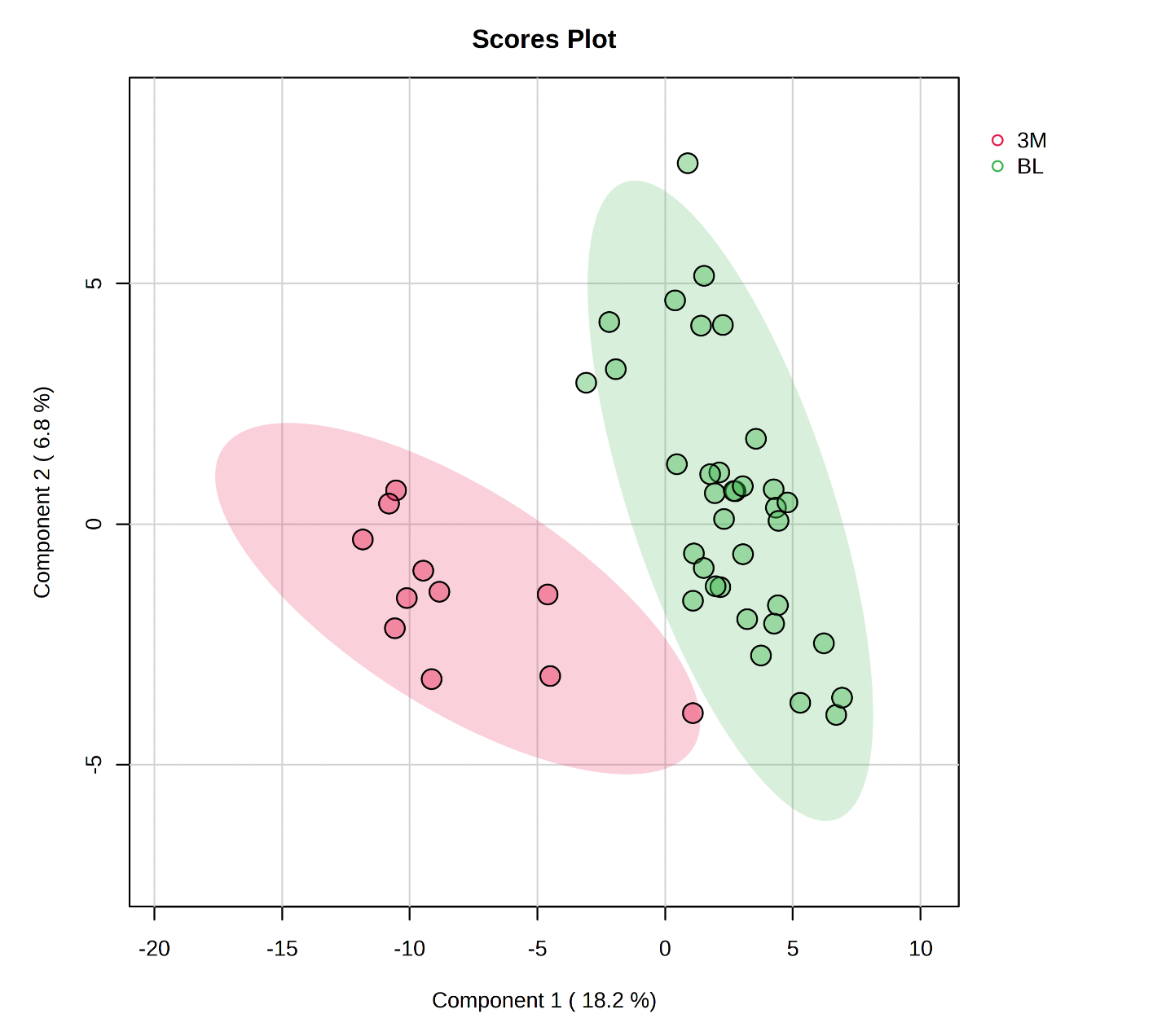


**Supplementary Figure S3B**: Serum Data, PLSDA scores of only females of Tertile 3 at three months post-sleeve gastrectomy compared with only female patients at baseline.


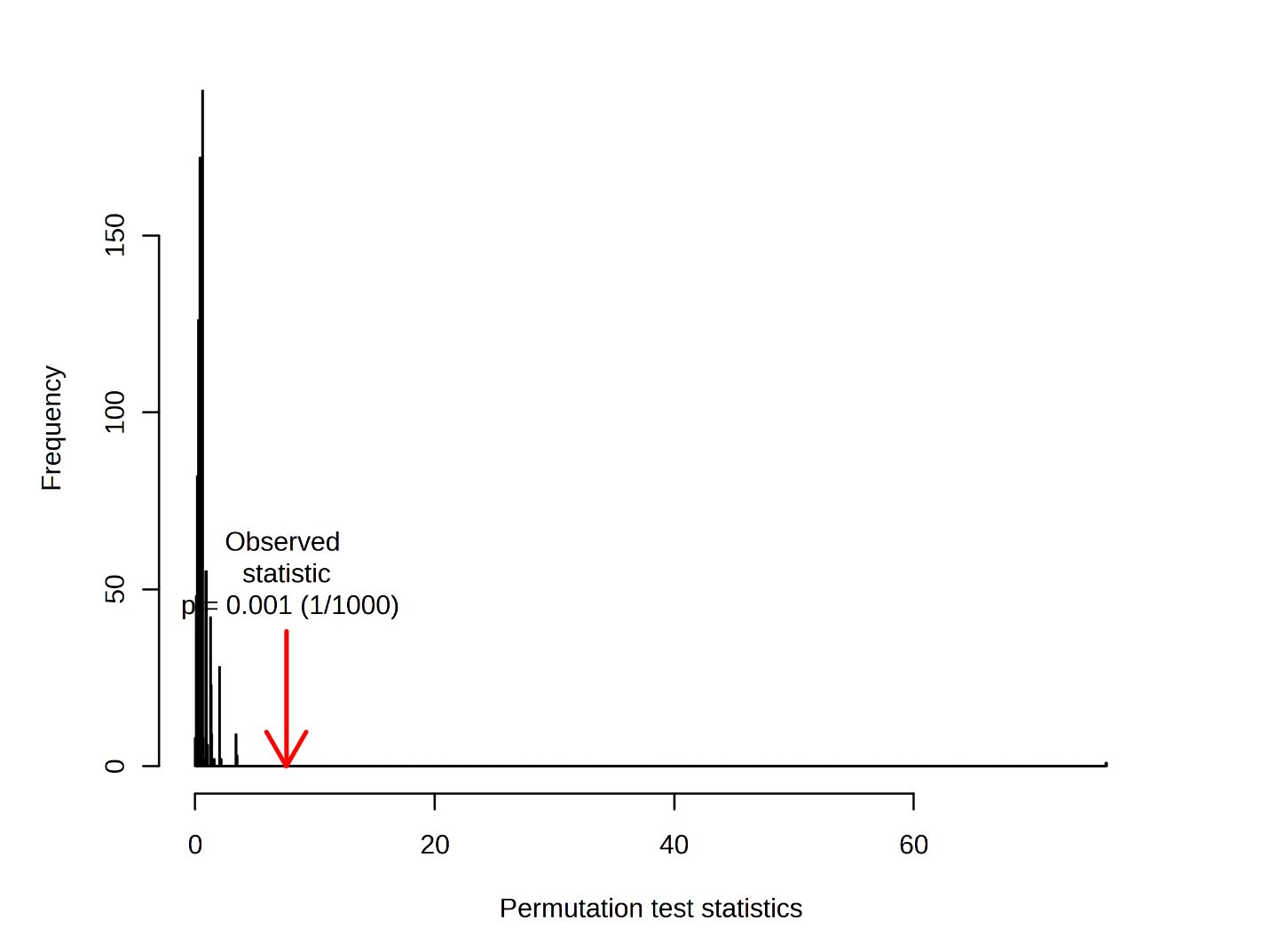


**Supplementary Figure S3C**: Serum Data, Permutation test of only females of Tertile 3 at three months post-sleeve gastrectomy compared with only female patients at baseline.


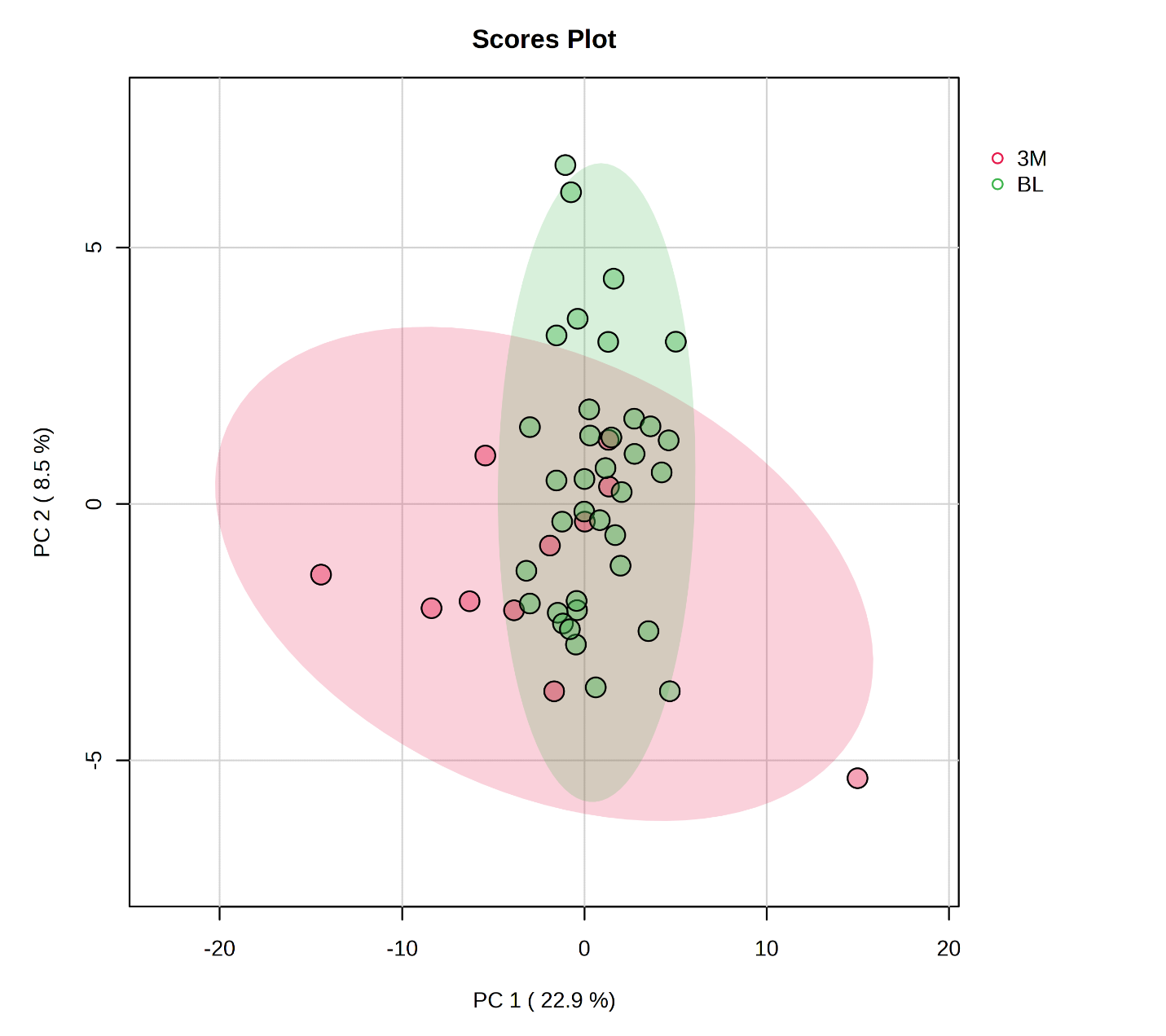


**Supplementary Figure S3D**: Fecal Data, PCA scores of only females of Tertile 3 at three months post-sleeve gastrectomy compared with only female patients at baseline.


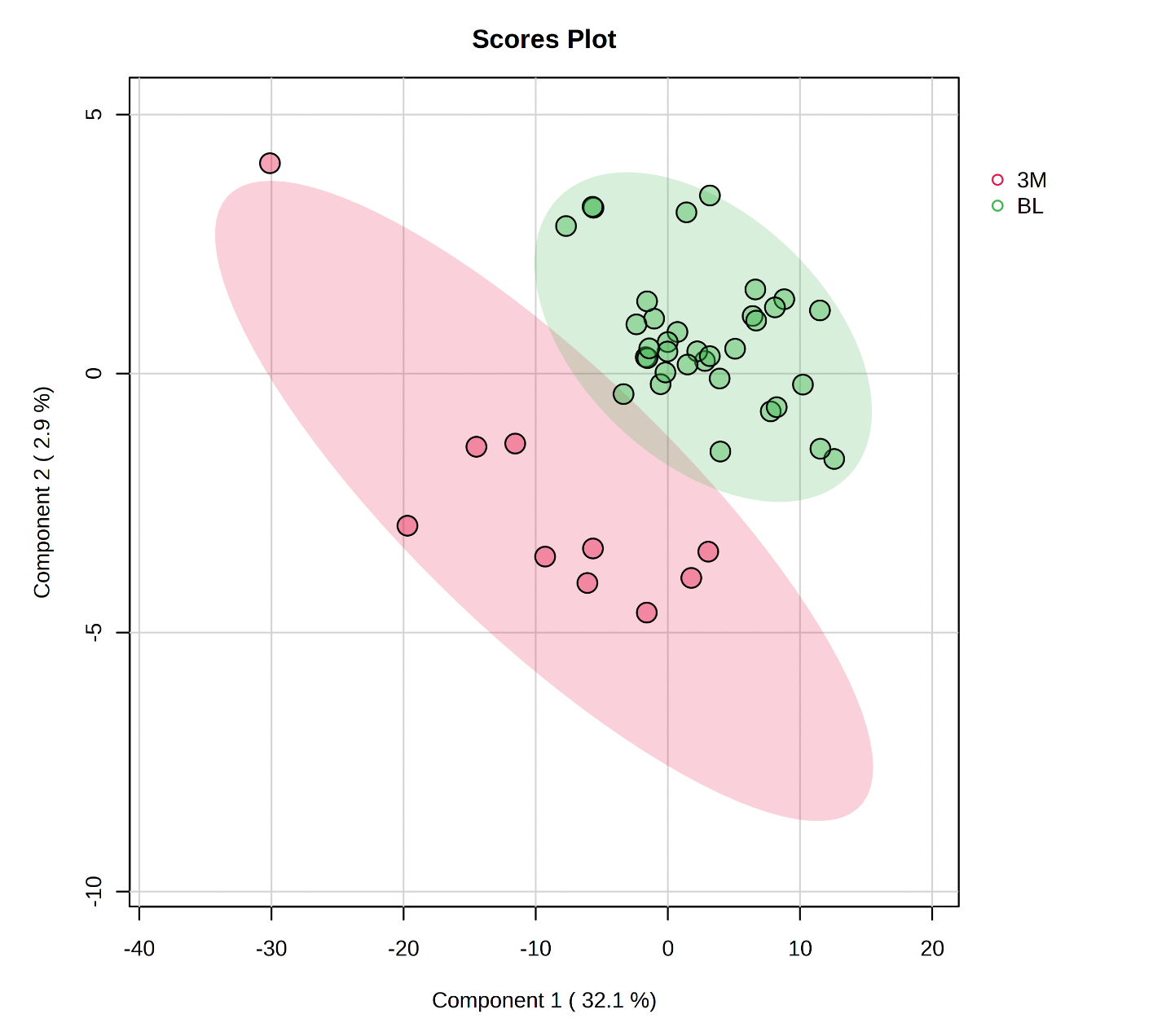


**Supplementary Figure S3E**: Fecal Data, PLSDA scores of only females of Tertile 3 at three months post-sleeve gastrectomy compared with only female patients at baseline.


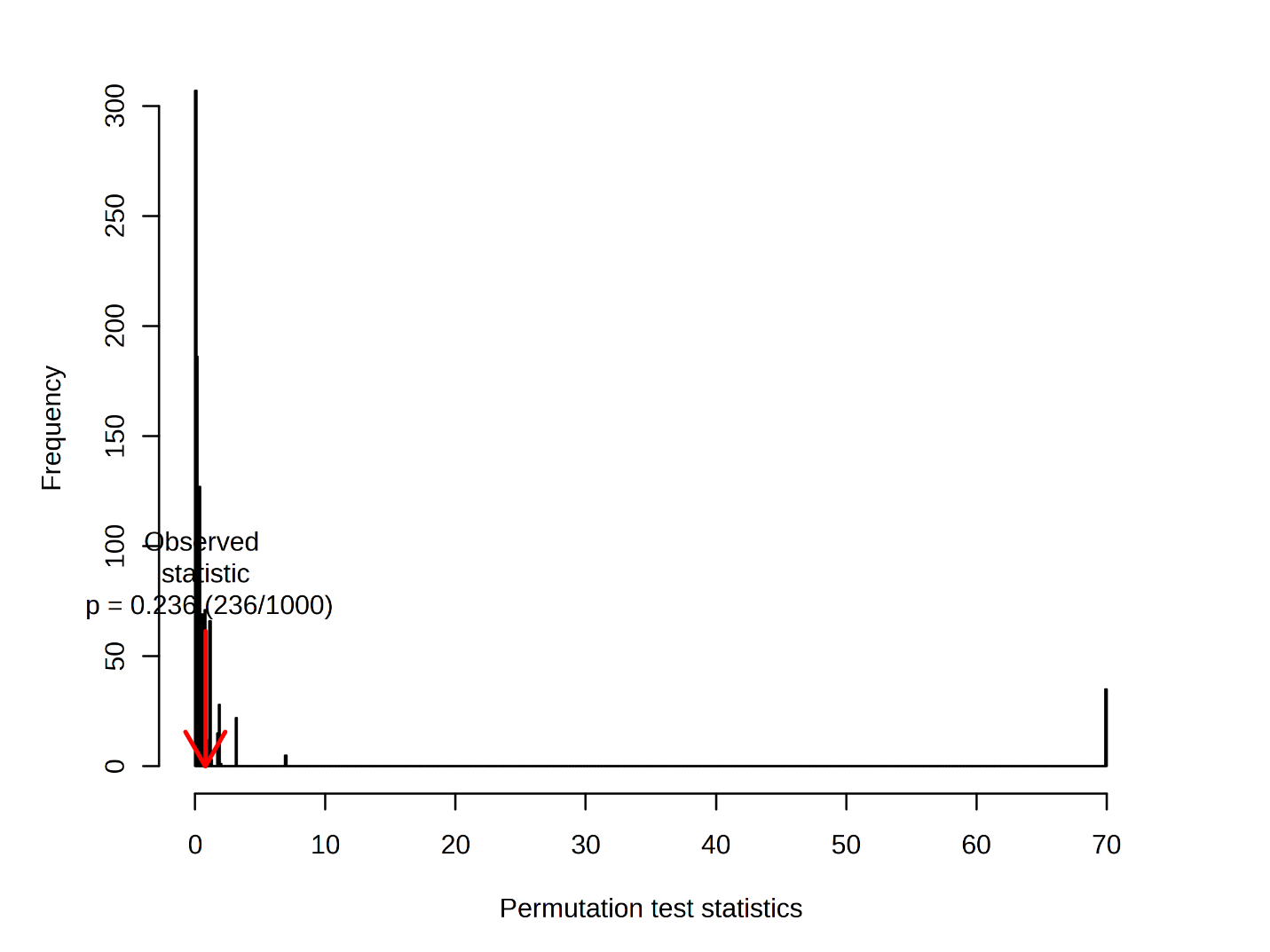

**Supplementary Figure S3F**: Fecal Data, Permutation test of only females of Tertile 3 at three months post-sleeve gastrectomy compared with only female patients at baseline.
