# Supplementary material for: Association of Metabolomic Biomarkers with Sleeve Gastrectomy Weight Loss Outcomes
Source: Metabolites. 2023 Mar 31;13(4):506. doi: 10.3390/metabo13040506 (PMC10145663; doi:10.3390/metabo13040506)
Supplement: Supplementary file 1 [file metabolites-13-00506-s001.zip › Supplementary Information Methods.pdf]

Supplementary Information

Methods

Metabolomic Analyses

^{1}H NMR Analysis Sample Preparation

^{1}H NMR Data Acquisition

Two hundred and fifty-six transients were acquired. Chemical shifts are reported in parts per million (ppm) of the operating frequency. For profiling, spectral data collected from serum samples were analyzed using Bayesil that is a web system automatically identifies and quantifies metabolites in serum. For fecal samples, all 1D ^{1}H-NMR spectra were processed and profiled using the Chenomx NMR Suite Professional Software package (version 8.4, Chenomx Inc, Edmonton, AB) using customized library of metabolites based on our literature search. For quality assurance, each spectrum was analyzed to determine if they demonstrated perfect Lorentzian line shape (indicating magnetic homogeneity during the data collection) and exhibited a flat baseline with symmetric signals.

DI-LC/MS/MS Sample Preparation and Analysis

Briefly, 200 mg fecal samples were extracted twice by first using extraction solvent A (85% ethanol and 15% phosphate-buffered saline solution) and then by extraction solvent B (20% ethanol and 80% phosphate-buffered saline solution). In total 10 µl of extraction solvent, the internal standards, 7 calibration standards, 3 quality controls, fecal extracts, and serum samples were directly applied to the kit plate and then dried under nitrogen (Waters, Milford, MA, USA). Amino acids and biogenic amines were derivatized using 50 µl PITC (phenylisothiocyanate). Subsequently, the metabolites were eluted with

5 mM ammonium acetate in methanol, incubated for 20 mins and then dried under nitrogen. The extracts were attained by centrifugation and then diluted for Liquid Chromatography (LC) and Flow Injection Analysis (FIA). Ultra-Performance Liquid Chromatography (UPLC) system coupled with a column (Waters ACQUITY UPLC BEH C18 1.7 µm 2.1 x 100 mm; Waters, Manchester, UK) was used for separation of the polar metabolites and the remaining metabolites were analyzed via the FIA method. Data acquisition was performed by using MetIQ software (BIOCRATES Life Sciences AG, Innsbruck, Austria).
