# Supplementary material for: Association of Metabolomic Biomarkers with Sleeve Gastrectomy Weight Loss Outcomes
Source: Metabolites. 2023 Mar 31;13(4):506. doi: 10.3390/metabo13040506 (PMC10145663; doi:10.3390/metabo13040506)
Supplement: Supplementary file 1 [file metabolites-13-00506-s001.zip › Supplementary Table 1.docx]

**Table S1**: Serum Univariate analysis of Tertile 3 at three months post-sleeve gastrectomy compared with all patients at baseline (Mean Concentration: μM)

| **Metabolites** | **Mean (SD) of 3M** | **Mean (SD) of BL** | **p-value** | **Fold Change** |
| --- | --- | --- | --- | --- |
| 2-Hydroxybutyric acid | 74.693 (74.246) | 37.867 (15.726) | < 0.0001 (W) | 1.94 |
| 3-Hydroxybutyric acid | 450.807 (402.488) | 42.145 (42.174) | < 0.0001 (W) | 10.02 |
| Acetoacetate | 139.607 (123.264) | 21.218 (16.261) | < 0.0001 (W) | 6.3 |
| Acetone | 343.164 (924.393) | 18.870 (11.635) | < 0.0001 (W) | 12.58 |
| Acetylcarnitine (C2) | 12.413 (4.605) | 7.279 (3.002) | < 0.0001 (W) | 1.93 |
| C3-DC (C4-OH) | 0.114 (0.061) | 0.063 (0.022) | < 0.0001 (W) | 1.92 |
| Citric acid | 166.993 (144.493) | 70.225 (26.177) | < 0.0001 (W) | 2.39 |
| Glutamate (Glu) | 34.757 (10.394) | 75.670 (31.221) | < 0.0001 (W) | -1.93 |
| Phosphatidylcholine (PC aa C32:2) | 1.287 (1.026) | 2.944 (1.353) | < 0.0001 (W) | -1.98 |
| Phosphatidylcholine (PC aa C34:4) | 0.465 (0.277) | 1.079 (0.423) | < 0.0001 (W) | -2.09 |
| Phosphatidylcholine (PC aa C36:6) | 0.252 (0.185) | 0.554 (0.202) | 0.0001 | -2.02 |
| Kynurenine | 1.627 (0.353) | 2.557 (0.567) | 0.0004 | -1.38 |
| Octadecenoylcarnitine (C18:1) | 0.241 (0.170) | 0.146 (0.053) | 0.0005 (W) | 1.91 |
| Creatine | 18.614 (14.473) | 46.195 (24.300) | 0.0005 (W) | -2 |
| Hexadecanoylcarnitine (C16) | 0.120 (0.075) | 0.078 (0.024) | 0.0007 (W) | 1.81 |
| Phosphatidylcholine (PC aa C38:3) | 20.736 (6.904) | 36.990 (12.240) | 0.0013 | -1.58 |
| Phosphatidylcholine (PC aa C34:3) | 5.611 (2.720) | 9.963 (3.205) | 0.0015 | -1.57 |
| Phosphatidylcholine (PC aa C36:5) | 7.468 (4.479) | 13.316 (5.099) | 0.0018 (W) | -1.61 |
| Phosphatidylcholine (PC aa C36:2) | 121.850 (37.541) | 190.355 (39.616) | 0.0022 | -1.38 |
| Asparagine (Asp) | 9.612 (3.003) | 14.882 (4.663) | 0.0031 (W) | -1.39 |
| Tyrosine (Tyr) | 76.386 (22.230) | 127.515 (40.739) | 0.0033 (W) | -1.48 |
| Ethanol | 149.079 (244.060) | 66.392 (46.373) | 0.0043 (W) | 1.87 |
| Glycine (Gly) | 363.643 (77.632) | 306.325 (104.358) | 0.0052 | 1.34 |
| α-Aminoadipic acid (alpha-AAA) | 0.690 (0.642) | 1.504 (1.145) | 0.0057 (W) | -2.07 |
| Succinate | 27.457 (5.921) | 22.997 (6.939) | 0.0077 | 1.36 |
| Phosphatidylcholine (PC aa C36:1) | 22.757 (7.963) | 34.307 (10.146) | 0.0094 (W) | -1.33 |
| Phosphatidylcholine (PC aa C36:3) | 59.743 (23.645) | 90.800 (25.488) | 0.0107 (W) | -1.35 |
| Lysophosphatidylcholine (PC a C20:3) | 1.175 (0.462) | 2.020 (0.835) | 0.0113 (W) | -1.48 |
| Octadecadienylcarnitine (C18:2) | 0.111 (0.078) | 0.082 (0.027) | 0.0128 (W) | 1.58 |
| Acetic acid | 21.640 (9.954) | 23.122 (32.076) | 0.0144 (W) | 1.04 |
| Methionine (Met) | 21.950 (7.134) | 33.348 (9.476) | 0.015 | -1.35 |
| Tryptophan (Trp) | 54.450 (16.283) | 82.692 (24.397) | 0.0151 | -1.34 |
| Symmetric dimethylarginine (SDMA) | 0.541 (0.072) | 0.486 (0.121) | 0.0152 (W) | 1.26 |
| Sphingomyelin (SM (OH) C22:1) | 8.541 (2.885) | 12.358 (3.053) | 0.0225 | -1.28 |
| Octadecanoylcarnitine (C18) | 2.139 (1.372) | 1.464 (1.367) | 0.0239 (W) | 1.72 |
| Propionylcarnitine (C3) | 0.238 (0.089) | 0.399 (0.174) | 0.0252 (W) | -1.44 |
| Methionine sulfoxide (Met-SO) | 0.282 (0.335) | 1.147 (2.042) | 0.028 (W) | -4.01 |
| Sphingomyelin (SM C26:1) | 0.238 (0.082) | 0.191 (0.072) | 0.0304 | 1.33 |
| Hexoses (H1) | 6364.143 (3072.559) | 8104.500 (2902.740) | 0.0315 | -1.16 |
| Sphingomyelin (SM C24:1) | 53.014 (18.497) | 42.583 (11.590) | 0.0341 | 1.37 |
| Phosphatidylcholine (PC ae C38:0) | 1.061 (0.471) | 1.521 (0.448) | 0.0379 | -1.32 |
| Glutamine (Gln) | 678.071 (77.313) | 656.150 (80.972) | 0.0382 | 1.16 |
| Phosphatidylcholine (PC ae C32:1) | 1.836 (0.528) | 1.628 (0.410) | 0.0383 | 1.26 |
| Lysine (Lys) | 275.857 (47.612) | 373.300 (82.407) | 0.0385 | -1.21 |
| Leucine (Leu) | 187.000 (55.606) | 281.325 (121.743) | 0.0421 (W) | -1.35 |
| Sphingomyelin (SM C18:1) | 14.700 (5.706) | 11.493 (3.489) | 0.0424 | 1.43 |
| Phosphatidylcholine (PC ae C38:3) | 3.047 (0.895) | 4.295 (1.225) | 0.0442 | -1.27 |
| L-Histidine | 8.567 (13.794) | 26.707 (26.915) | 0.0464 (W) | -3.09 |
| L-Phenylalanine (Phe) | 39.879 (8.563) | 52.992 (17.344) | 0.048 | -1.19 |
| Phosphatidylcholine (PC aa C32:1) | 4.991 (2.393) | 8.203 (4.565) | 0.0511 (W) | -1.44 |
| Phosphatidylcholine (PC ae C36:5) | 9.931 (3.089) | 8.962 (1.990) | 0.0521 | 1.22 |
| Phosphatidylcholine (PC ae C38:2) | 1.371 (0.680) | 2.046 (0.675) | 0.0526 | -1.32 |
| Phosphatidylcholine (PC aa C40:6) | 13.311 (3.917) | 18.602 (5.851) | 0.0531 | -1.26 |
| Decanoylcarnitine (C10) | 0.807 (1.914) | 0.256 (0.129) | 0.0536 (W) | 3.81 |
| Valine (Val) | 300.571 (69.735) | 422.750 (120.353) | 0.0539 | -1.25 |
| Lysophosphatidylcholine (lysoPC a C28:1) | 0.181 (0.085) | 0.261 (0.074) | 0.054 | -1.3 |
| Dimethyl sulfone | 12.007 (7.851) | 17.637 (57.320) | 0.0616 (W) | -1.28 |
| Phosphatidylcholine (PC ae C40:5) | 3.404 (0.805) | 3.153 (0.777) | 0.0631 | 1.19 |
| Sphingomyelin (SM C18:0) | 27.079 (10.847) | 22.245 (6.392) | 0.0635 | 1.34 |
| Phosphatidylcholine (PC aa C40:5) | 5.235 (1.527) | 7.379 (2.576) | 0.0647 | -1.26 |
| Phosphatidylcholine (PC aa C40:4) | 1.678 (0.365) | 2.335 (0.768) | 0.0708 | -1.24 |
| Sphingomyelin (SM C16:0) | 108.450 (34.755) | 96.110 (22.852) | 0.0846 | 1.25 |
| Acetylornithine (Ac-Orn) | 0.254 (0.218) | 0.543 (0.498) | 0.0879 (W) | -1.92 |
| Serotonin | 0.230 (0.221) | 0.461 (0.416) | 0.0879 (W) | -1.69 |
| Ornithine (Orn) | 75.879 (22.779) | 105.703 (34.577) | 0.0882 | -1.24 |
| Glycerol | 349.921 (588.572) | 201.787 (134.953) | 0.0956 (W) | 1.52 |
| Phosphatidylcholine (PC ae C42:5) | 2.001 (0.564) | 1.849 (0.516) | 0.0989 | 1.18 |
| Phosphatidylcholine (PC ae C34:3) | 5.659 (1.706) | 5.148 (1.223) | 0.1083 (W) | 1.23 |
| Lysophosphatidylcholine (lysoPC a C18:0) | 16.759 (4.593) | 23.925 (8.231) | 0.1128 (W) | -1.24 |
| Phosphatidylcholine (PC ae C38:5 | 12.240 (3.315) | 11.555 (2.483) | 0.1153 | 1.16 |
| L-Arginine (Arg) | 39.807 (66.688) | 18.078 (18.832) | 0.1222 (W) | 1.9 |
| Phosphatidylcholine PC ae C44:5) | 1.386 (0.600) | 1.240 (0.466) | 0.1239 | 1.21 |
| Phosphatidylcholine (PC aa C32:0) | 10.071 (3.309) | 9.065 (2.151) | 0.1254 | 1.24 |
| Phosphatidylcholine (PC aa C42:5) | 0.183 (0.047) | 0.233 (0.077) | 0.1274 | -1.16 |
| Sphingomyelin (SM C16:1) | 19.064 (6.255) | 17.102 (4.327) | 0.1276 | 1.24 |
| Phosphatidylcholine (PC aa C38:4) | 70.686 (17.509) | 91.130 (22.960) | 0.1313 | -1.16 |
| Histamine | 0.248 (0.057) | 0.246 (0.056) | 0.1374 (W) | 1.11 |
| Sarcosine | 0.857 (0.279) | 1.122 (0.363) | 0.1374 (W) | -1.2 |
| Lysophosphatidylcholine (lysoPC a C16:1) | 1.632 (0.699) | 2.321 (0.987) | 0.1522 | -1.25 |
| Formate | 18.157 (4.306) | 34.587 (106.277) | 0.154 (W) | -1.71 |
| Sphingomyelin (SM C20:2) | 0.463 (0.268) | 0.359 (0.134) | 0.1599 (W) | 1.4 |
| Phosphatidylcholine (PC ae C42:2) | 0.251 (0.084) | 0.325 (0.088) | 0.1633 | -1.17 |
| Proline (Pro) | 217.786 (77.483) | 291.300 (84.223) | 0.1824 | -1.17 |
| Phosphatidylcholine (PC ae C36:1) | 4.871 (1.412) | 6.352 (1.667) | 0.1831 | -1.15 |
| Phosphatidylcholine (PC ae C36:3) | 3.556 (1.115) | 4.633 (1.175) | 0.1831 | -1.18 |
| Valerylcarnitine (C5) | 0.206 (0.234) | 0.215 (0.085) | 0.1849 (W) | 1.17 |
| Phosphatidylcholine (PC ae C32:2) | 0.495 (0.191) | 0.443 (0.123) | 0.1883 | 1.23 |
| Sphingomyelin (SM (OH) C16:1) | 3.051 (1.062) | 2.857 (0.945) | 0.1888 | 1.18 |
| Phosphatidylcholine (PC aa C30:0) | 1.901 (0.761) | 2.512 (0.913) | 0.1938 | -1.19 |
| Lysophosphatidylcholine (lysoPC a C20:4) | 7.243 (2.706) | 6.982 (2.617) | 0.1985 (W) | 1.19 |
| Phosphatidylcholine (PC ae C40:1) | 0.721 (0.276) | 0.881 (0.238) | 0.1985 (W) | -1.09 |
| Isopropyl alcohol | 2.013 (1.546) | 3.735 (2.548) | 0.2055 (W) | -1.49 |
| Phosphatidylcholine (PC aa C42:6) | 0.218 (0.041) | 0.288 (0.079) | 0.2055 (W) | -1.19 |
| Phosphatidylcholine (PC ae C36:2) | 6.878 (2.143) | 8.928 (2.373) | 0.2076 | -1.16 |
| Tetradecenoylcarnitine (C14:1) | 0.147 (0.169) | 0.100 (0.027) | 0.2276 (W) | 1.68 |
| Phosphatidylcholine (PC ae C30:0) | 0.141 (0.042) | 0.182 (0.055) | 0.2361 | -1.15 |
| D-Glucose | 4846.614 (2573.452) | 4835.062 (1692.741) | 0.2382 | 1.07 |
| Sphingomyelin (SM (OH) C24:1) | 0.870 (0.327) | 1.117 (0.291) | 0.2502 | -1.15 |
| Methanol | 300.343 (27.782) | 339.712 (112.245) | 0.2514 (W) | -1.02 |
| Phosphatidylcholine (PC aa C28:1) | 2.034 (0.821) | 2.655 (0.772) | 0.2594 | -1.16 |
| Creatinine | 57.571 (7.239) | 59.665 (17.937) | 0.2651 | 1.11 |
| Asymmetric dimethylarginine (ADMA) | 0.344 (0.170) | 0.465 (0.203) | 0.2681 (W) | -1.2 |
| Phosphatidylcholine (PC ae C30:1) | 0.087 (0.035) | 0.084 (0.035) | 0.27 | 1.18 |
| Phosphatidylcholine (PC ae C44:3) | 0.050 (0.018) | 0.063 (0.022) | 0.2785 | -1.16 |
| Phosphatidylcholine (PC ae C42:3) | 0.422 (0.140) | 0.528 (0.148) | 0.2826 | -1.13 |
| Phosphatidylcholine (PC aa C36:4) | 144.143 (34.816) | 145.597 (36.845) | 0.2853 | 1.1 |
| Malonate | 17.300 (42.195) | 9.482 (7.487) | 0.2946 (W) | 1.33 |
| Histidine (His) | 83.643 (13.063) | 103.662 (14.814) | 0.304 | -1.08 |
| Carnitine | 41.479 (64.965) | 27.820 (10.661) | 0.3132 (W) | 1.37 |
| Choline | 8.510 (14.692) | 4.575 (2.899) | 0.3132 (W) | 1.58 |
| Pyruvic acid | 53.086 (24.392) | 82.040 (61.742) | 0.3132 (W) | -1.2 |
| Putrescine | 0.138 (0.031) | 0.137 (0.045) | 0.3545 | 1.11 |
| Sphingomyelin (SM (OH) C14:1) | 5.534 (1.843) | 5.540 (1.748) | 0.3632 | 1.12 |
| Phosphatidylcholine (PC aa C38:6) | 44.964 (14.879) | 45.038 (14.501) | 0.3761 | 1.11 |
| Phosphatidylcholine (PC ae C40:4) | 1.724 (0.349) | 2.026 (0.460) | 0.3948 | -1.06 |
| Lysophosphatidylcholine (lysoPC a C18:1) | 13.837 (4.885) | 14.216 (4.905) | 0.3979 | 1.11 |
| Phosphatidylcholine (PC ae C34:0) | 0.605 (0.196) | 0.748 (0.217) | 0.4064 | -1.1 |
| Asparagine (Asn) | 39.386 (5.268) | 47.235 (9.920) | 0.4387 | -1.07 |
| Urea | 116.229 (85.658) | 131.273 (131.062) | 0.44 (W) | -1.06 |
| Phosphatidylcholine (PC ae C42:4) | 0.638 (0.191) | 0.769 (0.235) | 0.453 | -1.09 |
| Phosphatidylcholine (PC ae C36:0) | 0.371 (0.114) | 0.381 (0.098) | 0.4587 | 1.09 |
| Phosphatidylcholine (PC ae C44:4) | 0.225 (0.060) | 0.270 (0.076) | 0.4747 | -1.08 |
| Threonine (Thr) | 138.607 (30.485) | 164.825 (39.551) | 0.4908 | -1.07 |
| Phosphatidylcholine (PC ae C38:6) | 5.014 (1.864) | 5.082 (1.211) | 0.4936 | 1.08 |
| Butyrylcarnitine (C4) | 0.213 (0.171) | 0.218 (0.075) | 0.5002 (W) | 1.14 |
| Isoleucine | 61.157 (25.898) | 80.385 (42.338) | 0.5002 | -1.23 |
| Sphingomyelin (SM C24:0) | 14.883 (5.565) | 17.822 (3.915) | 0.5006 | -1.07 |
| Isobutyric acid | 9.579 (4.596) | 10.002 (4.454) | 0.5128 (W) | 1.03 |
| L-Alanine (Ala) | 60.314 (24.320) | 84.550 (44.299) | 0.5128 (W) | -1.27 |
| Lysophosphatidylcholine (lysoPC a C18:2) | 22.478 (11.000) | 27.745 (8.885) | 0.5128 (W) | -1.05 |
| Phosphatidylcholine (PC aa C38:0) | 1.659 (0.451) | 1.974 (0.592) | 0.52 | -1.08 |
| Phosphatidylcholine (PC ae C34:1) | 5.100 (1.443) | 5.335 (1.416) | 0.5208 | 1.07 |
| Phosphatidylcholine (PC aa C40:1) | 0.246 (0.035) | 0.261 (0.048) | 0.5366 | 1.06 |
| trans -4-Hydroxyproline(t4-OH-Pro) | 15.021 (6.002) | 18.233 (13.132) | 0.5513 (W) | -1.07 |
| Phosphatidylcholine (PC ae C38:1) | 0.878 (0.489) | 1.076 (0.687) | 0.5645 (W) | -1.08 |
| Propylene glycol | 1.001 (1.703) | 1.054 (1.794) | 0.5778 (W) | 1.11 |
| Phosphatidylcholine (PC ae C40:3) | 1.404 (0.440) | 1.646 (0.457) | 0.582 | -1.07 |
| L-Lactic acid | 1822.129 (767.681) | 2011.260 (1001.683) | 0.5913 | 1.02 |
| Carnitine (C0) | 28.893 (7.253) | 35.290 (10.226) | 0.6338 | -1.05 |
| Hypoxanthine | 0.264 (0.473) | 0.216 (0.241) | 0.6605 (W) | 1.02 |
| Lysophosphatidylcholine (lysoPC a C17:0) | 1.170 (0.369) | 1.450 (0.586) | 0.6747 (W) | -1.08 |
| Sphingomyelin (SM C26:0) | 0.092 (0.030) | 0.098 (0.028) | 0.6821 | 1.05 |
| Phosphatidylcholine (PC aa C42:1) | 0.168 (0.043) | 0.175 (0.068) | 0.6839 | 1.05 |
| Lysophosphatidylcholine (lysoPC a C16:0) | 84.979 (24.046) | 93.468 (29.695) | 0.6857 | 1.05 |
| Phosphatidylcholine (PC aa C34:1) | 116.757 (39.590) | 126.080 (40.922) | 0.6879 | 1.05 |
| Phosphatidylcholine (PC ae C34:2) | 6.454 (2.053) | 7.526 (1.921) | 0.7043 | -1.05 |
| Citrulline (Cit) | 25.693 (6.345) | 29.300 (8.357) | 0.7181 (W) | -1.02 |
| Betaine | 84.221 (220.936) | 31.540 (16.497) | 0.7772 (W) | 1.98 |
| Phosphatidylcholine (PC ae C44:6) | 0.894 (0.288) | 0.950 (0.317) | 0.7905 | 1.03 |
| Phosphatidylcholine (PC ae C38:4) | 8.466 (1.684) | 9.534 (2.046) | 0.8078 | -1.02 |
| Phosphatidylcholine (PC aa C32:3) | 0.318 (0.151) | 0.341 (0.100) | 0.8225 (W) | 1.04 |
| Phosphatidylcholine (PC ae C40:6) | 2.668 (0.646) | 2.881 (0.724) | 0.8389 | 1.02 |
| Phosphatidylcholine (PC aa C38:5) | 29.321 (10.040) | 33.345 (9.527) | 0.8424 | -1.02 |
| Phosphatidylcholine (PC ae C42:1) | 0.200 (0.064) | 0.220 (0.068) | 0.8683 | -1 |
| Phosphatidylcholine (PC aa C42:0) | 0.343 (0.110) | 0.381 (0.144) | 0.8841 | -1.02 |
| Taurine (Thr) | 66.564 (21.663) | 73.817 (30.620) | 0.8991 (W) | -1 |
| Phosphatidylcholine (PC ae C36:4) | 12.629 (3.415) | 14.022 (3.238) | 0.8994 | -1.01 |
| Phosphatidylcholine (PC aa C34:2) | 192.214 (28.211) | 217.775 (36.949) | 0.9349 | 1.01 |
| 1-Methylhistidine | 124.443 (52.775) | 135.390 (43.321) | 0.9456 (W) | -1 |
| Sphingomyelin (SM (OH) C22:2) | 8.657 (2.291) | 9.729 (2.778) | 0.976 | 1 |
| Phosphatidylcholine (PC aa C36:0) | 1.303 (0.612) | 1.390 (0.624) | 0.9948 | 1 |
| Phosphatidylcholine (PC ae C40:2) | 1.449 (0.486) | 1.590 (0.485) | 0.9963 | 1 |
| 2-Hydroxybutyric acid | 74.693 (74.246) | 37.867 (15.726) | < 0.0001 (W) | 1.94 |
| 3-Hydroxybutyric acid | 450.807 (402.488) | 42.145 (42.174) | < 0.0001 (W) | 10.02 |
| Acetoacetate | 139.607 (123.264) | 21.218 (16.261) | < 0.0001 (W) | 6.3 |
| Acetone | 343.164 (924.393) | 18.870 (11.635) | < 0.0001 (W) | 12.58 |
| Acetylcarnitine (C2) | 12.413 (4.605) | 7.279 (3.002) | < 0.0001 (W) | 1.93 |
| C3-DC (C4-OH) | 0.114 (0.061) | 0.063 (0.022) | < 0.0001 (W) | 1.92 |
| Citric acid | 166.993 (144.493) | 70.225 (26.177) | < 0.0001 (W) | 2.39 |
| Glutamate (Glu) | 34.757 (10.394) | 75.670 (31.221) | < 0.0001 (W) | -1.93 |
| Phosphatidylcholine (PC aa C32:2) | 1.287 (1.026) | 2.944 (1.353) | < 0.0001 (W) | -1.98 |
| Phosphatidylcholine (PC aa C34:4) | 0.465 (0.277) | 1.079 (0.423) | < 0.0001 (W) | -2.09 |
