# Supplementary material for: Association of Metabolomic Biomarkers with Sleeve Gastrectomy Weight Loss Outcomes
Source: Metabolites. 2023 Mar 31;13(4):506. doi: 10.3390/metabo13040506 (PMC10145663; doi:10.3390/metabo13040506)
Supplement: Supplementary file 1 [file metabolites-13-00506-s001.zip › Supplementary Table 2.docx]

**Table S2:** Serum Univariate analysis of Tertile 1 at three months post-sleeve gastrectomy compared with all patients at baseline (Mean Concentration: μM)

| **Metabolites** | **Mean (SD) of 3M** | **Mean (SD) of BL** | **p-value** | **Fold Change** |
| --- | --- | --- | --- | --- |
| Sphingomyelin (SM C18:1) | 16.592 (3.568) | 11.493 (3.489) | < 0.0001 | 1.64 |
| Sphingomyelin (SM C24:1) | 60.567 (11.148) | 42.583 (11.590) | < 0.0001 | 1.6 |
| 3-Hydroxybutyric acid | 209.458 (182.803) | 42.145 (42.174) | < 0.0001 (W) | 5.37 |
| Acetone | 56.025 (44.990) | 18.870 (11.635) | < 0.0001 (W) | 3.27 |
| Citric acid | 109.533 (37.458) | 70.225 (26.177) | < 0.0001 (W) | 1.77 |
| Acetoacetate | 80.292 (75.494) | 21.218 (16.261) | 0.0001 (W) | 4.16 |
| Octadecenoylcarnitine (C18:1) | 0.196 (0.044) | 0.146 (0.053) | 0.0001 | 1.56 |
| Phosphatidylcholine (PC ae C40:5) | 3.966 (0.842) | 3.153 (0.777) | 0.0002 | 1.41 |
| Sphingomyelin (SM C18:0) | 30.117 (6.687) | 22.245 (6.392) | 0.0002 (W) | 1.54 |
| Sphingomyelin (SM (OH) C16:1) | 3.761 (0.847) | 2.857 (0.945) | 0.0003 (W) | 1.5 |
| Phosphatidylcholine (PC ae C42:5) | 2.357 (0.658) | 1.849 (0.516) | 0.0005 (W) | 1.43 |
| Glycerol | 394.083 (138.340) | 306.325 (104.358) | 0.0007 | 1.48 |
| Phosphatidylcholine (PC aa C32:2) | 1.457 (0.590) | 2.944 (1.353) | 0.0007 (W) | -1.76 |
| Phosphatidylcholine PC ae C44:5) | 1.577 (0.454) | 1.240 (0.466) | 0.0009 (W) | 1.41 |
| Sphingomyelin (SM C16:0) | 114.733 (16.972) | 96.110 (22.852) | 0.0011 | 1.36 |
| Acetic acid | 60.667 (128.781) | 23.122 (32.076) | 0.0012 (W) | 3.35 |
| Phosphatidylcholine (PC aa C34:4) | 0.585 (0.203) | 1.079 (0.423) | 0.0012 (W) | -1.62 |
| Phosphatidylcholine (PC ae C36:5) | 10.977 (3.142) | 8.962 (1.990) | 0.0016 | 1.39 |
| Phosphatidylcholine (PC ae C38:5 | 13.758 (3.053) | 11.555 (2.483) | 0.0016 | 1.34 |
| 2-Hydroxybutyric acid | 57.325 (25.914) | 37.867 (15.726) | 0.0016 (W) | 1.78 |
| Phosphatidylcholine (PC aa C34:3) | 6.640 (1.699) | 9.963 (3.205) | 0.0018 | -1.33 |
| Phosphatidylcholine (PC ae C44:6) | 1.164 (0.313) | 0.950 (0.317) | 0.0026 (W) | 1.37 |
| Phosphatidylcholine (PC ae C40:6 | 3.405 (0.616) | 2.881 (0.724) | 0.0027 | 1.33 |
| Hexadecanoylcarnitine (C16) | 0.090 (0.019) | 0.078 (0.024) | 0.0029 (W) | 1.35 |
| Decanoylcarnitine (C10) | 0.353 (0.196) | 0.256 (0.129) | 0.0031 (W) | 1.56 |
| Succinate | 26.700 (5.980) | 22.997 (6.939) | 0.0034 (W) | 1.31 |
| Hypoxanthine | 0.283 (0.153) | 0.216 (0.241) | 0.0039 (W) | 1.4 |
| Sphingomyelin (SM C16:1) | 20.017 (3.105) | 17.102 (4.327) | 0.0042 | 1.32 |
| Phosphatidylcholine (PC aa C42:1) | 0.226 (0.071) | 0.175 (0.068) | 0.0046 | 1.45 |
| Sphingomyelin (SM C20:2) | 0.482 (0.152) | 0.359 (0.134) | 0.0046 (W) | 1.51 |
| Phosphatidylcholine (PC ae C38:4) | 10.807 (2.434) | 9.534 (2.046) | 0.0048 | 1.28 |
| Acetylcarnitine (C2) | 9.394 (3.476) | 7.279 (3.002) | 0.0053 (W) | 1.5 |
| Sphingomyelin (SM C26:1) | 0.248 (0.064) | 0.191 (0.072) | 0.0057 | 1.45 |
| Phosphatidylcholine (PC aa C38:6) | 53.567 (15.037) | 45.038 (14.501) | 0.0068 | 1.36 |
| Hydroxybutyrylcarnitine (C3-DC (C4-OH)) | 0.086 (0.039) | 0.063 (0.022) | 0.0071 (W) | 1.6 |
| Phosphatidylcholine (PC ae C40:2) | 1.929 (0.469) | 1.590 (0.485) | 0.0072 | 1.37 |
| Phosphatidylcholine (PC ae C34:3) | 5.820 (1.076) | 5.148 (1.223) | 0.0076 (W) | 1.27 |
| Formate | 20.413 (4.452) | 34.587 (106.277) | 0.0094 (W) | -1.48 |
| Phosphatidylcholine (PC aa C36:4) | 160.283 (41.259) | 145.597 (36.845) | 0.0108 | 1.27 |
| Phosphatidylcholine (PC aa C42:0) | 0.468 (0.142) | 0.381 (0.144) | 0.0111 | 1.38 |
| Octadecadienylcarnitine (C18:2) | 0.089 (0.014) | 0.082 (0.027) | 0.0119 | 1.28 |
| Valerylcarnitine (C5) | 0.133 (0.024) | 0.215 (0.085) | 0.0123 (W) | -1.39 |
| Phosphatidylcholine (PC ae C32:2) | 0.517 (0.095) | 0.443 (0.123) | 0.0142 | 1.31 |
| Phosphatidylcholine (PC aa C38:3) | 25.942 (4.694) | 36.990 (12.240) | 0.015 | -1.25 |
| Pyruvic acid | 41.438 (20.520) | 82.040 (61.742) | 0.015 (W) | -1.63 |
| Phosphatidylcholine (PC aa C36:0) | 1.792 (0.718) | 1.390 (0.624) | 0.0161 | 1.47 |
| Histamine | 0.261 (0.002) | 0.246 (0.056) | 0.0181 (W) | 1.2 |
| Sphingomyelin (SM (OH) C14:1) | 6.357 (1.246) | 5.540 (1.748) | 0.0182 | 1.3 |
| Phosphatidylcholine (PC ae C34:1) | 5.885 (0.925) | 5.335 (1.416) | 0.0186 | 1.25 |
| Tetradecenoylcarnitine (C14:1) | 0.112 (0.020) | 0.100 (0.027) | 0.0187 | 1.27 |
| Propionylcarnitine (C3) | 0.241 (0.065) | 0.399 (0.174) | 0.0193 (W) | -1.41 |
| Phosphatidylcholine (PC ae C32:1) | 1.884 (0.479) | 1.628 (0.410) | 0.0193 (W) | 1.3 |
| Phosphatidylcholine (PC aa C36:6) | 0.341 (0.102) | 0.554 (0.202) | 0.0203 | -1.42 |
| Glutamate | 45.650 (16.162) | 75.670 (31.221) | 0.0232 (W) | -1.42 |
| Phosphatidylcholine (PC aa C36:5) | 8.723 (3.265) | 13.316 (5.099) | 0.0246 (W) | -1.33 |
| Tryptophan | 59.433 (10.214) | 82.692 (24.397) | 0.0256 | -1.21 |
| Phosphatidylcholine (PC aa C32:0) | 9.794 (1.626) | 9.065 (2.151) | 0.029 | 1.23 |
| Phosphatidylcholine (PC aa C32:1) | 4.690 (1.123) | 8.203 (4.565) | 0.0294 (W) | -1.52 |
| Phosphatidylcholine (PC ae C38:6) | 5.581 (1.340) | 5.082 (1.211) | 0.0304 | 1.24 |
| Octadecanoylcarnitine (C18) | 1.923 (1.192) | 1.464 (1.367) | 0.0329 (W) | 1.61 |
| Isopropyl alcohol | 8.733 (24.506) | 3.735 (2.548) | 0.0329 (W) | 3.12 |
| Tyrosine | 82.608 (18.978) | 127.515 (40.739) | 0.0329 (W) | -1.35 |
| Symmetric dimethylarginine | 0.539 (0.148) | 0.486 (0.121) | 0.0369 (W) | 1.29 |
| Lysophosphatidylcholine (lysoPC a C20:4) | 7.742 (2.446) | 6.982 (2.617) | 0.0412 (W) | 1.28 |
| Dimethyl sulfone | 10.696 (4.656) | 17.637 (57.320) | 0.0435 (W) | -1.45 |
| Phosphatidylcholine (PC ae C42:3) | 0.578 (0.147) | 0.528 (0.148) | 0.0438 | 1.22 |
| Phosphatidylcholine (PC ae C40:3) | 1.836 (0.345) | 1.646 (0.457) | 0.0459 (W) | 1.25 |
| Sphingomyelin (SM (OH) C22:2) | 10.463 (1.654) | 9.729 (2.778) | 0.0482 | 1.22 |
| Phosphatidylcholine (PC aa C40:1) | 0.273 (0.060) | 0.261 (0.048) | 0.0551 | 1.19 |
| Phosphatidylcholine (PC ae C36:0) | 0.411 (0.087) | 0.381 (0.098) | 0.0583 | 1.22 |
| Glutamine | 653.083 (71.199) | 656.150 (80.972) | 0.0588 | 1.14 |
| α-Aminoadipic acid | 0.743 (0.206) | 1.504 (1.145) | 0.0628 (W) | -1.79 |
| Ethanol | 69.383 (27.248) | 66.392 (46.373) | 0.066 (W) | 1.19 |
| Phosphatidylcholine (PC aa C34:2) | 215.500 (33.495) | 217.775 (36.949) | 0.0841 | 1.13 |
| Lysophosphatidylcholine (lysoPC a C17:0) | 1.544 (0.455) | 1.450 (0.586) | 0.0884 | 1.22 |
| Putrescine | 0.149 (0.059) | 0.137 (0.045) | 0.097 | 1.24 |
| Phosphatidylcholine (PC aa C38:0) | 2.123 (0.563) | 1.974 (0.592) | 0.1011 | 1.21 |
| Phosphatidylcholine (PC ae C40:4) | 2.212 (0.555) | 2.026 (0.460) | 0.1016 (W) | 1.23 |
| Phosphatidylcholine (PC aa C40:5) | 5.497 (1.053) | 7.379 (2.576) | 0.1022 | -1.17 |
| Phosphatidylcholine (PC ae C30:1) | 0.096 (0.043) | 0.084 (0.035) | 0.1028 | 1.27 |
| Methionine | 24.167 (5.285) | 33.348 (9.476) | 0.1068 | -1.21 |
| Valine | 327.833 (47.476) | 422.750 (120.353) | 0.1276 | -1.13 |
| Kynurenine | 1.948 (0.424) | 2.557 (0.567) | 0.1321 | -1.14 |
| Lysophosphatidylcholine (PC a C20:3) | 1.377 (0.466) | 2.020 (0.835) | 0.1325 (W) | -1.28 |
| Sphingomyelin (SM C26:0) | 0.102 (0.032) | 0.098 (0.028) | 0.135 | 1.18 |
| Lysophosphatidylcholine (lysoPC a C18:1) | 14.914 (4.579) | 14.216 (4.905) | 0.1502 | 1.2 |
| Phosphatidylcholine (PC ae C36:2) | 9.195 (1.843) | 8.928 (2.373) | 0.1588 | 1.15 |
| Phosphatidylcholine (PC ae C42:4) | 0.832 (0.234) | 0.769 (0.235) | 0.1633 (W) | 1.2 |
| Phosphatidylcholine (PC ae C36:4) | 14.258 (3.429) | 14.022 (3.238) | 0.1807 | 1.15 |
| Phosphatidylcholine (PC ae C42:1) | 0.215 (0.031) | 0.220 (0.068) | 0.1841 (W) | 1.11 |
| Sarcosine | 0.936 (0.540) | 1.122 (0.363) | 0.199 (W) | -1.05 |
| Methanol | 324.242 (75.332) | 339.712 (112.245) | 0.2148 (W) | 1.1 |
| Carnitine (C0) | 26.725 (4.775) | 35.290 (10.226) | 0.2193 | -1.13 |
| Phosphatidylcholine (PC aa C36:1) | 27.233 (6.310) | 34.307 (10.146) | 0.223 | -1.11 |
| Phosphatidylcholine (PC ae C34:0) | 0.746 (0.142) | 0.748 (0.217) | 0.2335 | 1.13 |
| Malonate | 10.125 (14.681) | 9.482 (7.487) | 0.24 (W) | 1.32 |
| Phosphatidylcholine (PC aa C32:3) | 0.340 (0.064) | 0.341 (0.100) | 0.2554 | 1.13 |
| Leucine | 195.250 (35.059) | 281.325 (121.743) | 0.2672 (W) | -1.27 |
| Proline | 228.417 (61.309) | 291.300 (84.223) | 0.2672 | -1.12 |
| Phosphatidylcholine (PC aa C30:0) | 1.927 (0.439) | 2.512 (0.913) | 0.292 | -1.14 |
| Phosphatidylcholine (PC ae C38:1) | 1.085 (0.383) | 1.076 (0.687) | 0.3065 (W) | 1.13 |
| L-Lactic acid | 1381.067 (494.332) | 2011.260 (1001.683) | 0.3169 (W) | -1.24 |
| Phosphatidylcholine (PC ae C36:1) | 6.134 (0.983) | 6.352 (1.667) | 0.3252 | 1.1 |
| 1-Methylhistidine | 123.133 (22.130) | 135.390 (43.321) | 0.3276 (W) | 1.05 |
| Phosphatidylcholine (PC aa C34:1) | 122.142 (20.871) | 126.080 (40.922) | 0.3379 | 1.1 |
| Phosphatidylcholine (PC ae C44:4) | 0.268 (0.083) | 0.270 (0.076) | 0.3494 (W) | 1.1 |
| Phosphatidylcholine (PC ae C34:2) | 7.222 (1.104) | 7.526 (1.921) | 0.3552 | 1.07 |
| Betaine | 27.783 (8.578) | 31.540 (16.497) | 0.3607 (W) | 1.02 |
| Lysine | 301.667 (38.684) | 373.300 (82.407) | 0.3607 (W) | -1.08 |
| Sphingomyelin (SM (OH) C24:1) | 1.087 (0.325) | 1.117 (0.291) | 0.3697 | 1.1 |
| Carnitine | 20.450 (8.370) | 27.820 (10.661) | 0.3722 (W) | -1.19 |
| Phosphatidylcholine (PC aa C40:4) | 1.829 (0.424) | 2.335 (0.768) | 0.3745 | -1.11 |
| Lysophosphatidylcholine (lysoPC a C16:0) | 89.758 (21.061) | 93.468 (29.695) | 0.3778 | 1.11 |
| Creatine | 34.188 (19.389) | 46.195 (24.300) | 0.399 | -1.17 |
| Lysophosphatidylcholine (lysoPC a C16:1) | 1.792 (0.624) | 2.321 (0.987) | 0.4179 | -1.13 |
| Phosphatidylcholine (PC aa C36:2) | 156.708 (34.514) | 190.355 (39.616) | 0.4212 | -1.07 |
| Phosphatidylcholine (PC aa C36:3) | 74.058 (19.711) | 90.800 (25.488) | 0.4318 | -1.09 |
| Asparagine | 12.088 (3.580) | 14.882 (4.663) | 0.4457 (W) | -1.06 |
| Phosphatidylcholine (PC aa C38:5) | 31.258 (5.133) | 33.345 (9.527) | 0.4616 | 1.07 |
| Isoleucine | 69.950 (30.384) | 80.385 (42.338) | 0.4853 (W) | 1 |
| Phosphatidylcholine (PC ae C38:3) | 4.102 (0.773) | 4.295 (1.225) | 0.487 | 1.07 |
| Phosphatidylcholine (PC ae C38:2) | 1.913 (0.369) | 2.046 (0.675) | 0.5265 | 1.07 |
| Phosphatidylcholine (PC aa C42:6) | 0.233 (0.038) | 0.288 (0.079) | 0.5266 (W) | -1.09 |
| Phosphatidylcholine (PC aa C38:4) | 83.908 (15.889) | 91.130 (22.960) | 0.5358 | 1.06 |
| Phosphatidylcholine (PC ae C36:3) | 4.353 (0.749) | 4.633 (1.175) | 0.5388 | 1.05 |
| Urea | 86.592 (39.188) | 131.273 (131.062) | 0.5408 (W) | -1.33 |
| Isobutyric acid | 18.592 (35.258) | 10.002 (4.454) | 0.5696 (W) | 2.41 |
| trans -4-Hydroxyproline (t4-OH-Pro) | 15.155 (8.405) | 18.233 (13.132) | 0.6142 (W) | -1.06 |
| Phosphatidylcholine (PC ae C40:1) | 0.807 (0.170) | 0.881 (0.238) | 0.6447 | 1.05 |
| D-Glucose | 3988.375 (1492.187) | 4835.062 (1692.741) | 0.6759 (W) | -1.04 |
| Lysophosphatidylcholine (lysoPC a C18:0) | 20.183 (5.694) | 23.925 (8.231) | 0.6759 (W) | -1.03 |
| Creatinine | 50.312 (17.920) | 59.665 (17.937) | 0.6932 | -1.04 |
| Phosphatidylcholine (PC aa C40:6) | 16.912 (4.817) | 18.602 (5.851) | 0.6955 | 1.05 |
| Hexoses (H1) | 6726.583 (1319.075) | 8104.500 (2902.740) | 0.7075 (W) | -1.01 |
| Lysophosphatidylcholine (lysoPC a C18:2) | 24.783 (7.838) | 27.745 (8.885) | 0.7235 (W) | 1.02 |
| Butyrylcarnitine (C4) | 0.182 (0.053) | 0.218 (0.075) | 0.7271 | -1.04 |
| Ornithine | 88.067 (25.927) | 105.703 (34.577) | 0.7333 | -1.04 |
| Phosphatidylcholine (PC ae C44:3) | 0.055 (0.022) | 0.063 (0.022) | 0.7375 | -1.05 |
| Lysophosphatidylcholine (lysoPC a C28:1) | 0.221 (0.073) | 0.261 (0.074) | 0.7509 | -1.04 |
| L-Arginine | 15.542 (12.316) | 18.078 (18.832) | 0.7888 (W) | -1.02 |
| Threonine (Thr) | 147.583 (26.428) | 164.825 (39.551) | 0.7904 | 1.02 |
| Phosphatidylcholine (PC aa C28:1) | 2.292 (0.459) | 2.655 (0.772) | 0.8262 | -1.02 |
| Histidine (His) | 88.908 (8.639) | 103.662 (14.814) | 0.8272 | -1.01 |
| Serotonin | 0.414 (0.338) | 0.461 (0.416) | 0.8387 (W) | 1.11 |
| Phosphatidylcholine (PC ae C42:2) | 0.282 (0.060) | 0.325 (0.088) | 0.8528 | -1.02 |
| L-Alanine | 70.042 (33.396) | 84.550 (44.299) | 0.8555 (W) | -1.01 |
| Choline | 3.642 (1.600) | 4.580 (2.892) | 0.8723 (W) | -1.08 |
| Citrulline | 25.161 (10.062) | 29.300 (8.357) | 0.8892 (W) | -1.01 |
| Propylene glycol | 0.597 (0.580) | 1.054 (1.794) | 0.8892 (W) | -1.33 |
| Sphingomyelin (SM C24:0) | 15.522 (2.986) | 17.822 (3.915) | 0.8964 | -1.01 |
| Phosphatidylcholine (PC ae C38:0) | 1.321 (0.273) | 1.521 (0.448) | 0.9135 | -1.01 |
| Asymmetric dimethylarginine | 0.402 (0.202) | 0.466 (0.201) | 0.9232 (W) | -1.03 |
| L-Phenylalanine | 48.358 (13.519) | 52.992 (17.344) | 0.9232 (W) | 1.07 |
| Methionine sulfoxide | 1.163 (2.365) | 1.147 (2.042) | 0.9232 (W) | 1.1 |
| Sphingomyelin (SM (OH) C22:1) | 10.892 (2.095) | 12.358 (3.053) | 0.9377 | 1.01 |
| Taurine | 60.375 (12.670) | 73.817 (30.620) | 0.9402 (W) | -1.06 |
| Phosphatidylcholine (PC aa C42:5) | 0.207 (0.045) | 0.233 (0.077) | 0.9482 | 1.01 |
| Asparagine | 41.075 (5.422) | 47.235 (9.920) | 0.9676 | 1 |
| Phosphatidylcholine (PC ae C30:0) | 0.160 (0.035) | 0.182 (0.055) | 0.9733 | -1 |
| Acetylornithine | 0.512 (0.551) | 0.543 (0.498) | 0.9744 (W) | 1.13 |
