# Supplementary material for: Association of Metabolomic Biomarkers with Sleeve Gastrectomy Weight Loss Outcomes
Source: Metabolites. 2023 Mar 31;13(4):506. doi: 10.3390/metabo13040506 (PMC10145663; doi:10.3390/metabo13040506)
Supplement: Supplementary file 1 [file metabolites-13-00506-s001.zip › Supplementary Table 3.docx]

**Table S3:** Serum Metabolite Set Enrichment Analysis of Tertile 3 at three months post-sleeve gastrectomy compared with all patients at baseline.

| Pathway | Total Cmpd | Hits | Raw p | Holm p | FDR |
| --- | --- | --- | --- | --- | --- |
| Fatty Acid Biosynthesis | 35 | 4 | 1.01E-14 | 7.10E-13 | 7.10E-13 |
| Ketone Body Metabolism | 13 | 3 | 8.83E-12 | 6.09E-10 | 3.09E-10 |
| Tyrosine Metabolism | 72 | 4 | 1.71E-09 | 1.16E-07 | 3.99E-08 |
| Arachidonic Acid Metabolism | 69 | 2 | 3.63E-09 | 2.43E-07 | 6.34E-08 |
| Phenylalanine and Tyrosine Metabolism | 28 | 4 | 8.01E-09 | 5.29E-07 | 1.12E-07 |
| Butyrate Metabolism | 19 | 2 | 1.97E-08 | 1.28E-06 | 2.30E-07 |
| Propanoate Metabolism | 42 | 3 | 8.25E-08 | 5.28E-06 | 8.25E-07 |
| Warburg Effect | 58 | 7 | 1.57E-07 | 9.88E-06 | 1.37E-06 |
| Citric Acid Cycle | 32 | 3 | 9.44E-07 | 5.85E-05 | 7.34E-06 |
| Transfer of Acetyl Groups into Mitochondria | 22 | 3 | 4.32E-06 | 0.000264 | 2.82E-05 |
| Beta-Alanine Metabolism | 34 | 3 | 4.44E-06 | 0.000266 | 2.82E-05 |
| Nicotinate and Nicotinamide Metabolism | 37 | 2 | 5.38E-06 | 0.000317 | 3.14E-05 |
| Purine Metabolism | 74 | 5 | 6.56E-06 | 0.00038 | 3.53E-05 |
| Malate-Aspartate Shuttle | 10 | 2 | 7.62E-06 | 0.000435 | 3.81E-05 |
| Glutathione Metabolism | 21 | 3 | 8.74E-06 | 0.00049 | 3.83E-05 |
| Valine, Leucine and Isoleucine Degradation | 60 | 6 | 8.75E-06 | 0.00049 | 3.83E-05 |
| Oxidation of Branched Chain Fatty Acids | 26 | 4 | 1.26E-05 | 0.00068 | 5.19E-05 |
| Tryptophan Metabolism | 60 | 6 | 3.23E-05 | 0.001714 | 0.000119 |
| Alanine Metabolism | 17 | 4 | 3.47E-05 | 0.001807 | 0.000119 |
| Glutamate Metabolism | 49 | 7 | 3.52E-05 | 0.001807 | 0.000119 |
| Folate Metabolism | 29 | 2 | 3.58E-05 | 0.001807 | 0.000119 |
| Ammonia Recycling | 32 | 7 | 4.33E-05 | 0.00212 | 0.000138 |
| Cysteine Metabolism | 26 | 2 | 5.68E-05 | 0.002728 | 0.000173 |
| Histidine Metabolism | 43 | 4 | 9.26E-05 | 0.004354 | 0.00027 |
| Beta Oxidation of Very Long Chain Fatty Acids | 17 | 2 | 0.000119 | 0.005463 | 0.000333 |
| Arginine and Proline Metabolism | 53 | 10 | 0.000129 | 0.005797 | 0.000337 |
| Lysine Degradation | 30 | 3 | 0.00013 | 0.005797 | 0.000337 |
| Glucose-Alanine Cycle | 13 | 4 | 0.000276 | 0.011852 | 0.000689 |
| Amino Sugar Metabolism | 33 | 4 | 0.000316 | 0.013268 | 0.000763 |
| Glycine and Serine Metabolism | 59 | 11 | 0.000468 | 0.019183 | 0.001092 |
| Fatty acid Metabolism | 43 | 2 | 0.00086 | 0.03441 | 0.001943 |
| Catecholamine Biosynthesis | 20 | 1 | 0.00172 | 0.067083 | 0.003592 |
| Thyroid hormone synthesis | 13 | 1 | 0.00172 | 0.067083 | 0.003592 |
| Urea Cycle | 29 | 9 | 0.001745 | 0.067083 | 0.003592 |
| Aspartate Metabolism | 35 | 8 | 0.003379 | 0.12163 | 0.006757 |
| Methionine Metabolism | 43 | 7 | 0.003686 | 0.12901 | 0.007167 |
| Carnitine Synthesis | 22 | 4 | 0.004966 | 0.16885 | 0.009395 |
| Porphyrin Metabolism | 40 | 1 | 0.008583 | 0.28325 | 0.015811 |
| Mitochondrial Electron Transport Chain | 19 | 1 | 0.014613 | 0.46763 | 0.024968 |
| Phytanic Acid Peroxisomal Oxidation | 26 | 1 | 0.014613 | 0.46763 | 0.024968 |
| Ethanol Degradation | 19 | 2 | 0.014624 | 0.46763 | 0.024968 |
| Methylhistidine Metabolism | 4 | 1 | 0.021677 | 0.62864 | 0.036129 |
| Spermidine and Spermine Biosynthesis | 18 | 3 | 0.022223 | 0.62864 | 0.036177 |
| Betaine Metabolism | 21 | 3 | 0.036939 | 0.99734 | 0.058766 |
| Bile Acid Biosynthesis | 65 | 2 | 0.040377 | 1 | 0.062809 |
| Biotin Metabolism | 8 | 1 | 0.051628 | 1 | 0.077032 |
| Glycerolipid Metabolism | 25 | 1 | 0.051721 | 1 | 0.077032 |
| Sphingolipid Metabolism | 40 | 2 | 0.076716 | 1 | 0.11188 |
| Galactose Metabolism | 38 | 2 | 0.078617 | 1 | 0.11231 |
| Pyrimidine Metabolism | 59 | 1 | 0.10169 | 1 | 0.13957 |
| Phenylacetate Metabolism | 9 | 1 | 0.10169 | 1 | 0.13957 |
| Mitochondrial Beta-Oxidation of Long Chain Saturated Fatty Acids | 28 | 2 | 0.10651 | 1 | 0.14337 |
| Selenoamino Acid Metabolism | 28 | 1 | 0.23381 | 1 | 0.30039 |
| Lactose Synthesis | 20 | 1 | 0.23602 | 1 | 0.30039 |
| Lactose Degradation | 9 | 1 | 0.23602 | 1 | 0.30039 |
| Mitochondrial Beta-Oxidation of Short Chain Saturated Fatty Acids | 27 | 1 | 0.26729 | 1 | 0.33411 |
| Pyruvate Metabolism | 48 | 4 | 0.29215 | 1 | 0.35878 |
| Glycolysis | 25 | 2 | 0.31109 | 1 | 0.37546 |
| Pyruvaldehyde Degradation | 10 | 1 | 0.33821 | 1 | 0.40127 |
| Gluconeogenesis | 35 | 3 | 0.4352 | 1 | 0.50773 |
| Phospholipid Biosynthesis | 29 | 3 | 0.46306 | 1 | 0.53138 |
| Threonine and 2-Oxobutanoate Degradation | 20 | 1 | 0.49369 | 1 | 0.55739 |
| D-Arginine and D-Ornithine Metabolism | 11 | 1 | 0.6542 | 1 | 0.72689 |
| Phosphatidylcholine Biosynthesis | 14 | 1 | 0.7294 | 1 | 0.75992 |
| Phosphatidylethanolamine Biosynthesis | 12 | 1 | 0.7294 | 1 | 0.75992 |
| Pterine Biosynthesis | 29 | 1 | 0.74907 | 1 | 0.75992 |
| Steroid Biosynthesis | 48 | 1 | 0.74907 | 1 | 0.75992 |
| Androgen and Estrogen Metabolism | 33 | 1 | 0.74907 | 1 | 0.75992 |
| Androstenedione Metabolism | 24 | 1 | 0.74907 | 1 | 0.75992 |
| Taurine and Hypotaurine Metabolism | 12 | 1 | 0.81746 | 1 | 0.81746 |
