# Supplementary material for: Association of Metabolomic Biomarkers with Sleeve Gastrectomy Weight Loss Outcomes
Source: Metabolites. 2023 Mar 31;13(4):506. doi: 10.3390/metabo13040506 (PMC10145663; doi:10.3390/metabo13040506)
Supplement: Supplementary file 1 [file metabolites-13-00506-s001.zip › Supplementary Table 4.docx]

**Table S4.** Serum Metabolite Set Enrichment Analysis of Tertile 1 at three months post-sleeve gastrectomy compared with all patients at baseline.

| **Pathway** | **Total Cmpd** | **Hits** | **Raw p** | **Holm p** | **FDR** |
| --- | --- | --- | --- | --- | --- |
| Ketone Body Metabolism | 13 | 3 | 7.60E-07 | 5.32E-05 | 4.39E-05 |
| Fatty Acid Biosynthesis | 35 | 4 | 1.26E-06 | 8.66E-05 | 4.39E-05 |
| Butyrate Metabolism | 19 | 2 | 1.67E-05 | 0.001137 | 0.00039 |
| Citric Acid Cycle | 32 | 3 | 3.05E-05 | 0.002041 | 0.000533 |
| Transfer of Acetyl Groups into Mitochondria | 22 | 3 | 5.67E-05 | 0.003744 | 0.000794 |
| Phenylalanine and Tyrosine Metabolism | 28 | 4 | 8.75E-05 | 0.005685 | 0.00102 |
| Warburg Effect | 58 | 7 | 0.000136 | 0.00871 | 0.001361 |
| Tyrosine Metabolism | 72 | 4 | 0.000203 | 0.012804 | 0.001671 |
| Oxidation of Branched Chain Fatty Acids | 26 | 4 | 0.000215 | 0.013321 | 0.001671 |
| Arachidonic Acid Metabolism | 69 | 2 | 0.000479 | 0.029211 | 0.003352 |
| Propanoate Metabolism | 42 | 3 | 0.00092 | 0.055207 | 0.005855 |
| Histidine Metabolism | 43 | 4 | 0.001079 | 0.063685 | 0.006052 |
| Purine Metabolism | 74 | 5 | 0.001124 | 0.065187 | 0.006052 |
| Sphingolipid Metabolism | 40 | 2 | 0.001409 | 0.080303 | 0.007044 |
| Alanine Metabolism | 17 | 4 | 0.001607 | 0.089988 | 0.007208 |
| Ammonia Recycling | 32 | 7 | 0.001647 | 0.09061 | 0.007208 |
| Porphyrin Metabolism | 40 | 1 | 0.001888 | 0.10197 | 0.007463 |
| Methylhistidine Metabolism | 4 | 1 | 0.001919 | 0.10197 | 0.007463 |
| Amino Sugar Metabolism | 33 | 4 | 0.002073 | 0.10777 | 0.007636 |
| Fatty acid Metabolism | 43 | 2 | 0.002217 | 0.11308 | 0.007663 |
| Carnitine Synthesis | 22 | 4 | 0.002299 | 0.11495 | 0.007663 |
| Glutathione Metabolism | 21 | 3 | 0.002621 | 0.12843 | 0.007681 |
| Beta-Alanine Metabolism | 34 | 3 | 0.002627 | 0.12843 | 0.007681 |
| Glutamate Metabolism | 49 | 7 | 0.002633 | 0.12843 | 0.007681 |
| Beta Oxidation of Very Long Chain Fatty Acids | 17 | 2 | 0.003295 | 0.15156 | 0.009225 |
| Valine, Leucine and Isoleucine Degradation | 60 | 6 | 0.003578 | 0.16103 | 0.009634 |
| Cysteine Metabolism | 26 | 2 | 0.005767 | 0.25375 | 0.014952 |
| Pyruvate Metabolism | 48 | 4 | 0.006907 | 0.29698 | 0.017266 |
| Mitochondrial Electron Transport Chain | 19 | 1 | 0.008051 | 0.33814 | 0.018786 |
| Phytanic Acid Peroxisomal Oxidation | 26 | 1 | 0.008051 | 0.33814 | 0.018786 |
| Nicotinate and Nicotinamide Metabolism | 37 | 2 | 0.010224 | 0.40897 | 0.023087 |
| Bile Acid Biosynthesis | 65 | 2 | 0.014012 | 0.54645 | 0.03065 |
| Ethanol Degradation | 19 | 2 | 0.015993 | 0.60772 | 0.033924 |
| Glucose-Alanine Cycle | 13 | 4 | 0.018817 | 0.69625 | 0.038742 |
| Catecholamine Biosynthesis | 20 | 1 | 0.026431 | 0.95151 | 0.051393 |
| Thyroid hormone synthesis | 13 | 1 | 0.026431 | 0.95151 | 0.051393 |
| Arginine and Proline Metabolism | 53 | 10 | 0.030965 | 1 | 0.057596 |
| Folate Metabolism | 29 | 2 | 0.031624 | 1 | 0.057596 |
| Pyruvaldehyde Degradation | 10 | 1 | 0.032089 | 1 | 0.057596 |
| Glycolysis | 25 | 2 | 0.042278 | 1 | 0.073986 |
| Gluconeogenesis | 35 | 3 | 0.045306 | 1 | 0.077351 |
| Glycine and Serine Metabolism | 59 | 11 | 0.054993 | 1 | 0.091655 |
| Malate-Aspartate Shuttle | 10 | 2 | 0.057054 | 1 | 0.092879 |
| Methionine Metabolism | 43 | 7 | 0.066897 | 1 | 0.10595 |
| Lysine Degradation | 30 | 3 | 0.06811 | 1 | 0.10595 |
| Galactose Metabolism | 38 | 2 | 0.069709 | 1 | 0.10608 |
| Pyrimidine Metabolism | 59 | 1 | 0.07471 | 1 | 0.10765 |
| Phenylacetate Metabolism | 9 | 1 | 0.07471 | 1 | 0.10765 |
| Glycerolipid Metabolism | 25 | 1 | 0.075406 | 1 | 0.10765 |
| Aspartate Metabolism | 35 | 8 | 0.076894 | 1 | 0.10765 |
| Mitochondrial Beta-Oxidation of Long Chain Saturated Fatty Acids | 28 | 2 | 0.087301 | 1 | 0.11982 |
| Tryptophan Metabolism | 60 | 6 | 0.09793 | 1 | 0.13183 |
| Mitochondrial Beta-Oxidation of Short Chain Saturated Fatty Acids | 27 | 1 | 0.10321 | 1 | 0.13632 |
| Phospholipid Biosynthesis | 29 | 3 | 0.14687 | 1 | 0.18409 |
| Lactose Synthesis | 20 | 1 | 0.14858 | 1 | 0.18409 |
| Lactose Degradation | 9 | 1 | 0.14858 | 1 | 0.18409 |
| Urea Cycle | 29 | 9 | 0.1499 | 1 | 0.18409 |
| Spermidine and Spermine Biosynthesis | 18 | 3 | 0.21665 | 1 | 0.26148 |
| Pterine Biosynthesis | 29 | 1 | 0.28739 | 1 | 0.32447 |
| Steroid Biosynthesis | 48 | 1 | 0.28739 | 1 | 0.32447 |
| Androgen and Estrogen Metabolism | 33 | 1 | 0.28739 | 1 | 0.32447 |
| Androstenedione Metabolism | 24 | 1 | 0.28739 | 1 | 0.32447 |
| Betaine Metabolism | 21 | 3 | 0.51879 | 1 | 0.57643 |
| Biotin Metabolism | 8 | 1 | 0.58707 | 1 | 0.64211 |
| Threonine and 2-Oxobutanoate Degradation | 20 | 1 | 0.64799 | 1 | 0.69784 |
| Phosphatidylcholine Biosynthesis | 14 | 1 | 0.67949 | 1 | 0.70992 |
| Phosphatidylethanolamine Biosynthesis | 12 | 1 | 0.67949 | 1 | 0.70992 |
| D-Arginine and D-Ornithine Metabolism | 11 | 1 | 0.71262 | 1 | 0.73358 |
| Selenoamino Acid Metabolism | 28 | 1 | 0.94316 | 1 | 0.95683 |
| Taurine and Hypotaurine Metabolism | 12 | 1 | 0.99742 | 1 | 0.99742 |
