# Supplementary material for: Association of Metabolomic Biomarkers with Sleeve Gastrectomy Weight Loss Outcomes
Source: Metabolites. 2023 Mar 31;13(4):506. doi: 10.3390/metabo13040506 (PMC10145663; doi:10.3390/metabo13040506)
Supplement: Supplementary file 1 [file metabolites-13-00506-s001.zip › Supplementary Table 5.docx]

**Table S5**: Fecal Univariate analysis of Tertile 3 at three months post-sleeve gastrectomy compared with all patients at baseline (Mean concentration: μM)

| **Metabolites** | **Mean (SD) of 3M** | **Mean (SD) of BL** | **P-value** | **Fold Change** |
| --- | --- | --- | --- | --- |
| Lysophosphatidylcholine (lysoPC a C14:0) | 4.073 (0.259) | 3.891 (0.282) | < 0.0001 (W) | 1.71 |
| Phosphatidylcholine (PC aa C26:0) | 0.224 (0.017) | 0.214 (0.017) | < 0.0001 (W) | 1.72 |
| Phosphatidylcholine (PC aa C40:1) | 0.102 (0.015) | 0.096 (0.014) | < 0.0001 (W) | 1.77 |
| Phosphatidylcholine (PC ae C42:0) | 0.222 (0.017) | 0.222 (0.014) | < 0.0001 (W) | 1.64 |
|  |  |  |  |  |
| Phosphatidylcholine (PC ae C42:5) | 0.271 (0.022) | 0.276 (0.023) | 0.0001 (W) | -1.6 |
| Phosphatidylcholine (PC aa C42:0) | 0.061 (0.010) | 0.061 (0.009) | 0.0002 (W) | 1.56 |
| Phosphatidylcholine (PC aa C38:4) | 0.054 (0.009) | 0.051 (0.008) | 0.0003 (W) | 1.75 |
| Phosphatidylcholine (PC ae C40:6) | 0.033 (0.006) | 0.033 (0.006) | 0.0005 (W) | 1.58 |
| Phosphatidylcholine (PC aa C40:4) | 0.058 (0.006) | 0.059 (0.008) | 0.0006 (W) | -1.58 |
| Phosphatidylcholine (PC aa C42:4) | 0.045 (0.009) | 0.044 (0.008) | 0.0006 (W) | 1.66 |
| Phosphatidylcholine (PC aa C40:2) | 0.086 (0.014) | 0.089 (0.012) | 0.0007 (W) | -1.58 |
| Phosphatidylcholine (PC aa C42:2) | 0.036 (0.005) | 0.038 (0.008) | 0.0007 (W) | -1.53 |
| Phosphatidylcholine (PC ae C40:1) | 0.047 (0.007) | 0.049 (0.007) | 0.0007 (W) | -1.56 |
| Sphingomyelin (SM C20:2) | 0.089 (0.014) | 0.088 (0.020) | 0.0007 (W) | 1.59 |
| Phosphatidylcholine (PC aa C36:0) | 0.172 (0.025) | 0.172 (0.023) | 0.0008 (W) | 1.61 |
| Phosphatidylcholine (PC ae C36:0) | 0.036 (0.006) | 0.038 (0.006) | 0.0008 (W) | -1.56 |
| Phosphatidylcholine (PC ae C38:4) | 0.045 (0.008) | 0.046 (0.008) | 0.0008 (W) | -1.57 |
| Phosphatidylcholine (PC ae C40:3) | 0.095 (0.011) | 0.098 (0.017) | 0.0008 (W) | -1.53 |
| Phosphatidylcholine (PC aa C40:3) | 0.185 (0.023) | 0.191 (0.022) | 0.0009 (W) | -1.57 |
| Butenylcarnitine (C4:1) | 0.051 (0.016) | 0.050 (0.015) | 0.001 (W) | 1.59 |
| Phosphatidylcholine (PC ae C42:3) | 0.050 (0.007) | 0.050 (0.008) | 0.0011 (W) | -1.59 |
| Phosphatidylcholine (PC ae C38:0) | 0.023 (0.003) | 0.024 (0.004) | 0.0012 (W) | -1.54 |
| Sphingomyelin (SM C24:0) | 0.080 (0.016) | 0.093 (0.074) | 0.0012 (W) | -1.45 |
| Phosphatidylcholine (PC aa C40:6) | 0.097 (0.012) | 0.099 (0.014) | 0.0014 (W) | -1.6 |
| Phosphatidylcholine (PC ae C40:4) | 0.036 (0.005) | 0.038 (0.008) | 0.0014 (W) | -1.5 |
| Phosphatidylcholine (PC ae C32:2) | 0.027 (0.005) | 0.028 (0.005) | 0.0015 (W) | -1.55 |
| Phosphatidylcholine (PC aa C38:3) | 0.216 (0.031) | 0.220 (0.026) | 0.0016 (W) | -1.58 |
| Phosphatidylcholine (PC ae C40:2) | 0.068 (0.011) | 0.069 (0.012) | 0.0016 (W) | -1.53 |
| Phosphatidylcholine (PC ae C42:1) | 0.043 (0.007) | 0.043 (0.009) | 0.0016 (W) | -1.55 |
| Phosphatidylcholine (PC ae C44:4) | 0.045 (0.008) | 0.048 (0.008) | 0.0016 (W) | -1.55 |
| Phosphatidylcholine PC ae C44:5) | 0.029 (0.005) | 0.031 (0.007) | 0.0016 (W) | -1.52 |
| Phosphatidylcholine (PC a C24:0) | 0.097 (0.016) | 0.095 (0.016) | 0.0017 (W) | 1.64 |
| Phosphatidylcholine (PC ae C40:5) | 0.056 (0.008) | 0.058 (0.009) | 0.0017 (W) | -1.58 |
| Phosphatidylcholine (PC ae C44:3) | 0.038 (0.006) | 0.037 (0.008) | 0.0017 (W) | 1.6 |
| Phosphatidylcholine (PC aa C24:0) | 0.037 (0.006) | 0.039 (0.014) | 0.0018 (W) | -1.41 |
| Phosphatidylcholine (PC a C28:1) | 0.048 (0.012) | 0.048 (0.012) | 0.002 (W) | 1.69 |
| Phosphatidylcholine (PC aa C42:1) | 0.041 (0.007) | 0.041 (0.006) | 0.002 (W) | 1.58 |
| Phosphatidylcholine (PC a C26:0) | 0.113 (0.013) | 0.120 (0.014) | 0.0021 (W) | -1.54 |
| Phosphatidylcholine (PC aa C32:0) | 0.193 (0.042) | 0.213 (0.089) | 0.0021 (W) | -1.47 |
| Phosphatidylcholine (PC aa C38:6) | 0.031 (0.005) | 0.031 (0.006) | 0.0025 (W) | -1.63 |
| Phosphatidylcholine (PC ae C42:2) | 0.042 (0.007) | 0.043 (0.006) | 0.0025 (W) | -1.61 |
| Phosphatidylcholine (PC aa C32:3) | 0.035 (0.005) | 0.037 (0.007) | 0.0031 (W) | -1.45 |
| Phosphatidylcholine (PC a C28:0) | 0.121 (0.015) | 0.129 (0.015) | 0.0033 (W) | -1.52 |
| Dodecenoylcarnitine (C12:1) | 0.438 (0.133) | 0.393 (0.144) | 0.0035 (W) | 1.74 |
| Phosphatidylcholine (PC ae C34:0) | 0.046 (0.007) | 0.048 (0.009) | 0.0035 (W) | -1.58 |
| Phosphatidylcholine (PC ae C38:5 | 0.045 (0.007) | 0.048 (0.009) | 0.0035 (W) | -1.57 |
| Phosphatidylcholine (PC aa C36:6) | 0.017 (0.002) | 0.018 (0.010) | 0.0038 (W) | -1.55 |
| Octanoylcarnitine (C8) | 0.062 (0.008) | 0.067 (0.013) | 0.004 (W) | -1.52 |
| Phosphatidylcholine (PC aa C34:4) | 0.023 (0.004) | 0.024 (0.004) | 0.004 (W) | -1.53 |
| Phosphatidylcholine (PC aa C38:0) | 0.036 (0.005) | 0.039 (0.007) | 0.004 (W) | -1.5 |
| Phosphatidylcholine (PC aa C40:5) | 0.026 (0.006) | 0.026 (0.007) | 0.0043 (W) | -1.58 |
| Decenoylcarnitine (C10:1) | 0.327 (0.071) | 0.320 (0.099) | 0.005 (W) | 1.7 |
| Phosphatidylcholine (PC ae C30:0) | 0.030 (0.005) | 0.034 (0.009) | 0.0061 (W) | -1.4 |
| Dodecanedioylcarnitine (C12-DC) | 0.071 (0.016) | 0.077 (0.024) | 0.0065 (W) | -1.49 |
| Phosphatidylcholine (PC aa C36:5) | 0.022 (0.004) | 0.025 (0.022) | 0.0065 (W) | -1.48 |
| Phosphatidylcholine (PC ae C44:6) | 0.040 (0.007) | 0.041 (0.006) | 0.0073 (W) | -1.59 |
| Sphingomyelin (SM (OH) C22:2) | 0.017 (0.008) | 0.015 (0.011) | 0.0073 (W) | 1.92 |
| trans -4-Hydroxyproline (t4-OH-Pro) | 1.048 (1.539) | 7.385 (27.371) | 0.0083 (W) | -5.61 |
| Phosphatidylcholine (PC aa C34:3) | 0.017 (0.005) | 0.023 (0.028) | 0.0089 (W) | -1.25 |
| Phosphatidylcholine (PC ae C36:4) | 0.063 (0.011) | 0.070 (0.023) | 0.0094 (W) | -1.46 |
| Phosphatidylcholine (PC ae C38:2) | 0.047 (0.010) | 0.049 (0.017) | 0.0094 (W) | -1.58 |
| Phosphatidylcholine (PC ae C38:3) | 0.078 (0.014) | 0.087 (0.020) | 0.0094 (W) | -1.48 |
| Taurine | 8.182 (13.285) | 56.947 (91.191) | 0.0094 (W) | -4.7 |
| Lysophosphatidylcholine (PC a C20:3) | 0.113 (0.031) | 0.117 (0.027) | 0.0104 | -1.55 |
| Phosphatidylcholine (PC aa C38:5) | 0.030 (0.005) | 0.036 (0.024) | 0.0113 (W) | -1.26 |
| Phosphatidylcholine (PC ae C32:1) | 0.021 (0.004) | 0.023 (0.014) | 0.0113 (W) | -1.44 |
| Phosphatidylcholine (PC ae C36:5) | 0.029 (0.006) | 0.033 (0.007) | 0.012 (W) | -1.46 |
| Decanoylcarnitine (C10) | 0.047 (0.011) | 0.058 (0.020) | 0.0128 (W) | -1.38 |
| Phosphatidylcholine (PC ae C38:1) | 0.104 (0.014) | 0.102 (0.019) | 0.0133 | 1.68 |
| Tiglylcarnitine (C5:1) | 0.031 (0.008) | 0.038 (0.030) | 0.0144 (W) | -1.26 |
| Phosphatidylcholine (PC ae C38:6) | 0.018 (0.004) | 0.021 (0.006) | 0.0161 (W) | -1.42 |
| Acetic acid | 788.214 (491.840) | 1410.917 (510.929) | 0.017 | -1.36 |
| Dodecanoylcarnitine (C12) | 0.060 (0.017) | 0.067 (0.036) | 0.0181 (W) | -1.5 |
| Sphingomyelin (SM C16:1) | 0.045 (0.009) | 0.049 (0.023) | 0.0191 (W) | -1.46 |
| Phosphatidylcholine (PC a C26:1) | 0.030 (0.008) | 0.030 (0.010) | 0.0202 (W) | -1.63 |
| Phosphatidylcholine (PC aa C42:5) | 0.039 (0.006) | 0.044 (0.008) | 0.0226 (W) | -1.44 |
| Phosphatidylcholine (PC ae C30:2) | 0.016 (0.004) | 0.017 (0.003) | 0.0226 (W) | -1.59 |
| Sphingomyelin (SM (OH) C24:1) | 0.015 (0.009) | 0.012 (0.006) | 0.0239 (W) | 2.04 |
| Phosphatidylcholine (PC aa C32:2) | 0.024 (0.008) | 0.027 (0.011) | 0.0252 (W) | -1.48 |
| Isobutyric acid | 682.849 (113.921) | 1029.718 (1542.053) | 0.0266 (W) | -1.2 |
| Phosphatidylcholine (PC ae C34:3) | 0.033 (0.006) | 0.040 (0.022) | 0.0266 (W) | -1.3 |
| Xanthine | 1.499 (1.484) | 5.385 (25.405) | 0.0266 (W) | -2.38 |
| Phosphatidylcholine (PC ae C36:3) | 0.038 (0.006) | 0.046 (0.019) | 0.028 (W) | -1.31 |
| Symmetric dimethylarginine (SDMA) | 0.291 (0.619) | 1.119 (3.859) | 0.0295 (W) | -3.16 |
| Tetradecadienoylcarnitine (C14:2) | 0.017 (0.007) | 0.021 (0.013) | 0.0311 (W) | -1.42 |
| Hexenoylcarnitine (C6:1) | 0.029 (0.006) | 0.035 (0.009) | 0.0345 (W) | -1.34 |
| Spermidine | 38.899 (124.191) | 74.152 (142.590) | 0.0363 (W) | -4.61 |
| Hydroxyhexadecenoylcarnitine (C16:1-OH) | 0.232 (0.190) | 0.181 (0.185) | 0.0381 (W) | 2.25 |
| Phosphatidylcholine (PC aa C42:6) | 0.042 (0.014) | 0.045 (0.012) | 0.0381 (W) | -1.46 |
| Hydroxyhexadecanoylcarnitine (C16-OH) | 0.209 (0.173) | 0.163 (0.180) | 0.0442 (W) | 2.22 |
| Phosphatidylcholine (PC ae C36:1) | 0.089 (0.035) | 0.099 (0.035) | 0.0462 | -1.52 |
| p-Cresol | 70.186 (66.237) | 62.799 (46.335) | 0.0464 (W) | 2.62 |
| Phosphatidylcholine (PC aa C28:1) | 0.022 (0.007) | 0.025 (0.006) | 0.0536 (W) | -1.49 |
| Valerylcarnitine (C5) | 0.032 (0.010) | 0.047 (0.042) | 0.0588 (W) | -1.14 |
| Isovaleric acid | 0.969 (1.413) | 5.550 (14.047) | 0.0588 (W) | -3.54 |
| Hydroxytetradecenoylcarnitine (C14:1-OH) | 0.028 (0.014) | 0.033 (0.025) | 0.0616 (W) | -1.49 |
| Sphingomyelin (SM (OH) C22:1) | 0.019 (0.007) | 0.032 (0.066) | 0.0645 (W) | -1.02 |
| Lysophosphatidylcholine (lysoPC a C18:1) | 0.362 (0.573) | 0.630 (0.922) | 0.0675 (W) | -1.07 |
| Lysophosphatidylcholine (PC a C20:4) | 0.042 (0.015) | 0.062 (0.066) | 0.0675 (W) | -1.09 |
| Lysophosphatidylcholine (lysoPC a C17:0) | 0.099 (0.029) | 0.128 (0.073) | 0.0772 (W) | -1.2 |
| Acetylcarnitine (C2) | 0.060 (0.019) | 0.107 (0.213) | 0.0806 (W) | -1.17 |
| Butyrylcarnitine (C4) | 0.028 (0.009) | 0.039 (0.031) | 0.0879 (W) | -1.19 |
| Sphingomyelin (SM (OH) C14:1) | 0.016 (0.006) | 0.023 (0.031) | 0.1039 (W) | -1.13 |
| Urocanate | 13.388 (9.549) | 14.311 (8.521) | 0.1083 (W) | -2.13 |
| Methylamine | 22.015 (12.425) | 22.499 (10.226) | 0.1174 (W) | -1.9 |
| Fumarylcarnitine (C6 (C4:1-DC)) | 0.037 (0.009) | 0.045 (0.013) | 0.1244 | -1.31 |
| Phosphatidylcholine (PC aa C30:0) | 0.070 (0.022) | 0.096 (0.052) | 0.1271 (W) | -1.15 |
| Sphingomyelin (SM C26:0) | 0.012 (0.007) | 0.011 (0.006) | 0.1271 (W) | 1.69 |
| Nonaylcarnitine (C9) | 0.020 (0.006) | 0.031 (0.026) | 0.1322 (W) | -1.07 |
| Phosphatidylcholine (PC ae C30:1) | 0.008 (0.002) | 0.010 (0.004) | 0.1322 (W) | -1.37 |
| Histidine (His) | 8.755 (10.504) | 23.052 (27.933) | 0.1374 (W) | -1.74 |
| Phosphatidylcholine (PC ae C36:2) | 0.027 (0.005) | 0.036 (0.015) | 0.1374 (W) | 1.22 |
| Lysophosphatidylcholine (lysoPC a C18:0) | 0.672 (0.112) | 1.240 (1.756) | 0.1428 (W) | -1.08 |
| Octadecanoylcarnitine (C18) | 0.066 (0.048) | 0.145 (0.163) | 0.1659 (W) | -1.54 |
| Choline | 2.734 (2.839) | 4.891 (4.750) | 0.1659 (W) | -1.25 |
| 3-Hydroxybutyric acid | 9.769 (6.340) | 11.786 (13.525) | 0.1784 (W) | -1.27 |
| Phosphatidylcholine (PC ae C34:1) | 0.032 (0.009) | 0.057 (0.062) | 0.1784 (W) | -1.1 |
| Phosphatidylcholine (PC ae C34:2) | 0.032 (0.004) | 0.061 (0.070) | 0.1784 (W) | -1.19 |
| Citrulline (Cit) | 135.890 (78.473) | 251.661 (144.234) | 0.1824 | -1.26 |
| Butyrate | 474.642 (299.534) | 862.093 (616.086) | 0.1849 (W) | -1.33 |
| Tetradecanoylcarnitine (C14) | 0.033 (0.013) | 0.049 (0.034) | 0.1849 (W) | -1.12 |
| Methylglutarylcarnitine (C5-M-DC) | 0.027 (0.006) | 0.035 (0.011) | 0.2104 | -1.26 |
| Threonine (Thr) | 171.813 (70.489) | 222.194 (104.833) | 0.2151 | -1.17 |
| Hydroxytetradecadienoylcarnitine (C14:2-OH) | 0.014 (0.005) | 0.019 (0.009) | 0.2201 (W) | -1.16 |
| Propenoylcarnitine (C3:1) | 0.014 (0.008) | 0.018 (0.009) | 0.2276 (W) | -1.27 |
| Proline (Pro) | 191.199 (88.043) | 261.914 (137.346) | 0.2331 | -1.23 |
| Methanol | 0.496 (0.464) | 26.759 (158.031) | 0.2433 (W) | -29.4 |
| Lysophosphatidylcholine (lysoPC a C16:1) | 0.026 (0.017) | 0.041 (0.044) | 0.2597 (W) | -1.06 |
| Decadienoylcarnitine (C10:2) | 0.038 (0.012) | 0.065 (0.061) | 0.2681 (W) | -1.13 |
| Methylmalonylcarnitine (C5-DC (C6-OH)) | 0.027 (0.012) | 0.033 (0.010) | 0.2681 (W) | -1.34 |
| Lysophosphatidylcholine (lysoPC a C18:2) | 0.434 (0.686) | 0.776 (1.809) | 0.2946 (W) | -1.11 |
| N6-Acetyllysine | 27.905 (26.342) | 26.669 (23.594) | 0.2946 (W) | 2.16 |
| Sphingomyelin (SM C18:1) | 0.010 (0.008) | 0.019 (0.036) | 0.2946 (W) | -1.09 |
| Tetradecenoylcarnitine (C14:1) | 0.010 (0.003) | 0.016 (0.015) | 0.3228 (W) | -1.15 |
| Pimeloylcarnitine (C7-DC) | 0.019 (0.004) | 0.035 (0.042) | 0.3325 (W) | -1.17 |
| 2-Oxoglutarate | 28.062 (53.434) | 18.485 (13.293) | 0.3424 (W) | 4.44 |
| Methionine (Met) | 82.226 (30.770) | 121.675 (78.403) | 0.3424 (W) | -1.06 |
| Sphingomyelin (SM (OH) C16:1) | 0.015 (0.009) | 0.028 (0.044) | 0.3424 (W) | -1.05 |
| Fumaric acid | 17.950 (11.802) | 20.844 (14.157) | 0.3525 (W) | -1.37 |
| L-Fucose | 19.652 (19.459) | 28.320 (55.201) | 0.3525 (W) | -1.01 |
| Octadecadienylcarnitine (C18:2) | 0.024 (0.024) | 0.041 (0.051) | 0.3628 (W) | -1.14 |
| D-Glucose | 239.834 (223.570) | 425.231 (376.520) | 0.3628 (W) | -1.38 |
| Thymine | 35.928 (16.832) | 46.416 (23.188) | 0.3628 (W) | -1.3 |
| Hexadecadienoylcarnitine (C16:2) | 0.015 (0.005) | 0.024 (0.022) | 0.3733 (W) | -1.06 |
| Nicotinate | 11.629 (8.469) | 20.277 (12.557) | 0.3733 (W) | -1.07 |
| Propionate | 918.809 (657.503) | 1379.153 (679.798) | 0.3733 (W) | -1.16 |
| 3-Hydroxyisovaleric acid | 14.183 (18.318) | 22.650 (52.540) | 0.384 (W) | -1.04 |
| Hydroxyhexadecadienoylcarnitine (C16:2-OH) | 0.017 (0.005) | 0.029 (0.023) | 0.3948 (W) | -1.03 |
| Pyruvic acid | 6.278 (5.271) | 8.401 (5.789) | 0.3998 | -1.19 |
| Alanine (Ala) | 668.209 (261.695) | 1145.361 (570.460) | 0.4248 | -1.11 |
| Asparagine (Asn) | 107.028 (88.081) | 133.452 (92.492) | 0.4284 (W) | -1.23 |
| Carnitine (C0) | 2.572 (0.738) | 3.925 (2.393) | 0.4284 (W) | -1.12 |
| Hydroxyoctadecenoylcarnitine (C18:1-OH) | 0.024 (0.022) | 0.052 (0.094) | 0.4284 (W) | -1.48 |
| 1-Methylhistidine | 10.620 (12.290) | 15.083 (10.662) | 0.44 (W) | -1.63 |
| Caprylate | 59.521 (55.463) | 86.167 (204.573) | 0.44 (W) | -1.15 |
| Dimethylamine | 6.924 (12.606) | 5.343 (5.813) | 0.44 (W) | 3.7 |
| Hypoxanthine | 41.414 (23.699) | 52.856 (31.070) | 0.44 (W) | -1.31 |
| Hydroxybutyrylcarnitine(C3-DC (C4-OH)) | 0.118 (0.076) | 0.135 (0.085) | 0.4517 (W) | -1.37 |
| N-Acetylcysteine | 21.288 (18.559) | 22.976 (10.849) | 0.4517 (W) | -1.28 |
| Glycine (Gly) | 226.738 (117.663) | 296.679 (134.151) | 0.4746 | -1.15 |
| Phosphatidylcholine (PC aa C36:4) | 0.092 (0.141) | 0.325 (1.526) | 0.4756 (W) | -2.15 |
| Formate | 27.637 (15.904) | 34.777 (18.607) | 0.4878 (W) | -1.15 |
| Phenylalanine (Phe) | 221.231 (166.143) | 284.154 (191.112) | 0.4878 (W) | -1.08 |
| Phosphatidylcholine (PC aa C32:1) | 0.025 (0.014) | 0.034 (0.025) | 0.5002 (W) | -1.31 |
| Phosphatidylcholine (PC aa C36:3) | 0.109 (0.169) | 0.210 (0.750) | 0.5128 (W) | -1.2 |
| Uracil | 96.619 (51.594) | 132.057 (65.064) | 0.5658 | -1.09 |
| Lysophosphatidylcholine (lysoPC a C16:0) | 0.895 (0.508) | 2.213 (3.234) | 0.5778 (W) | -1.37 |
| Phosphatidylcholine (PC aa C34:1) | 0.197 (0.159) | 0.368 (0.404) | 0.6048 (W) | -1.29 |
| α-Aminoadipic acid (alpha-AAA) | 8.781 (14.135) | 15.219 (63.520) | 0.6186 (W) | -1.35 |
| Aspartate (Asp) | 121.117 (62.182) | 170.479 (98.923) | 0.6186 (W) | -1.03 |
| Hexadecanoylcarnitine (C16) | 0.065 (0.046) | 0.133 (0.170) | 0.6186 (W) | -1.39 |
| Serine (Ser) | 161.141 (85.707) | 255.905 (205.735) | 0.6186 (W) | -1.01 |
| Lysophosphatidylcholine (lysoPC a C16:1) | 0.052 (0.012) | 0.080 (0.040) | 0.6324 (W) | -1.13 |
| Valerate | 179.341 (155.906) | 239.750 (152.895) | 0.6324 (W) | -1 |
| Hydroxypropionylcarnitine (C3-OH) | 0.012 (0.004) | 0.017 (0.006) | 0.6482 | -1.09 |
| Glutaconylcarnitine (C5:1-DC) | 0.064 (0.020) | 0.100 (0.036) | 0.6484 | -1.08 |
| 4-Hydroxyphenyllactate | 22.345 (47.294) | 22.884 (30.009) | 0.6605 (W) | -1.62 |
| Creatinine | 14.907 (11.074) | 45.343 (87.702) | 0.6747 (W) | -1.99 |
| Tyrosine (Tyr) | 159.642 (81.552) | 283.432 (174.441) | 0.6793 | -1.07 |
| Phosphatidylcholine (PC aa C36:2) | 0.090 (0.072) | 0.202 (0.586) | 0.6891 (W) | -1.35 |
| Tryptophan (Trp) | 28.559 (12.684) | 45.378 (27.415) | 0.6952 | -1.07 |
| L-Lysine (Lys) | 135.713 (82.166) | 224.705 (186.741) | 0.7035 (W) | -1.05 |
| Arabinose | 60.368 (41.683) | 81.803 (69.134) | 0.7181 (W) | -1.04 |
| Propionylcarnitine (C3) | 0.023 (0.012) | 0.032 (0.016) | 0.7181 (W) | -1.05 |
| Glutamine (Gln) | 59.406 (38.102) | 98.243 (101.024) | 0.7181 (W) | -1.1 |
| Trimethylamine | 12.517 (15.809) | 11.341 (10.414) | 0.7181 (W) | 2.91 |
| Malonate | 55.646 (27.743) | 84.650 (39.903) | 0.728 | -1.05 |
| Isopropyl alcohol | 193.539 (97.977) | 265.294 (118.953) | 0.7349 | -1.04 |
| D-Galactose | 35.061 (20.811) | 75.359 (173.282) | 0.7475 (W) | -1.53 |
| Sphingomyelin (SM C18:0) | 0.052 (0.048) | 0.179 (0.312) | 0.7475 (W) | -2.07 |
| Creatine | 42.024 (31.811) | 55.438 (30.345) | 0.7623 (W) | -1.16 |
| Phosphatidylcholine (PC aa C36:1) | 0.040 (0.018) | 0.073 (0.070) | 0.7623 (W) | -1.03 |
| L-Phenylalanine (Phe) | 84.644 (35.650) | 127.908 (54.929) | 0.7699 | -1.04 |
| Acetylornithine (Ac-Orn) | 2706.145 (8875.251) | 496.622 (1348.812) | 0.7772 (W) | 2.09 |
| Glutamate (Glu) | 739.173 (321.929) | 1118.856 (557.396) | 0.7778 | -1.04 |
| Ethanol | 68.574 (76.098) | 119.934 (237.995) | 0.7922 (W) | -1.07 |
| Ornithine (Orn) | 79.292 (110.682) | 69.358 (54.847) | 0.7922 (W) | 1.27 |
| Isoleucine | 112.166 (49.471) | 156.446 (74.682) | 0.7955 | -1.04 |
| 3-Phenylpropionate | 38.066 (65.912) | 32.410 (19.280) | 0.8073 (W) | 3.29 |
| Phenylacetate | 56.468 (22.440) | 81.509 (47.041) | 0.8171 | -1.04 |
| Methionine sulfoxide | 12.861 (10.248) | 23.983 (23.341) | 0.853 | -1.01 |
| Serotonin | 0.430 (0.707) | 0.447 (0.515) | 0.853 (W) | -1.87 |
| Valine (Val) | 8.671 (14.790) | 18.462 (57.771) | 0.853 (W) | -1.03 |
| Acetone | 210.611 (116.047) | 311.244 (160.845) | 0.8631 | -1.02 |
| Hydroxyvalerylcarnitine(C5-OH (C3-DC-M)) | 0.039 (0.016) | 0.073 (0.059) | 0.8683 (W) | -1.21 |
| L-Lactic acid | 41.793 (45.754) | 45.955 (28.103) | 0.8683 (W) | -1.27 |
| Betaine | 11.349 (23.059) | 10.001 (13.175) | 0.8837 (W) | 3.39 |
| Octadecenoylcarnitine (C18:1) | 0.046 (0.042) | 0.095 (0.127) | 0.8837 (W) | -1.47 |
| Sphingomyelin (SM C16:0) | 0.279 (0.193) | 0.715 (1.266) | 0.8837 (W) | -1.56 |
| 4-Hydroxyphenylacetate | 9.098 (8.297) | 10.599 (7.280) | 0.9301 (W) | -1.26 |
| Sarcosine | 109.912 (281.825) | 139.453 (270.882) | 0.9611 (W) | -1.12 |
| Succinate | 80.691 (121.620) | 273.788 (739.267) | 0.9611 (W) | -1.91 |
| Acetoacetate | 7.054 (5.437) | 17.584 (34.714) | 0.9767 (W) | -1 |
| L-Arginine (Arg) | 8.938 (6.923) | 78.894 (226.042) | 0.9767 (W) | -6.26 |
| L-Leucine (Leu) | 39.991 (20.383) | 209.266 (631.389) | 0.9922 (W) | -2.63 |
| N-Acetylglutamate | 15.949 (12.871) | 20.095 (13.888) | 0.9922 (W) | -1.22 |
| Phosphatidylcholine (PC aa C34:2) | 0.135 (0.130) | 0.280 (0.617) | 0.9922 (W) | -1.3 |
| Putrescine | 12.563 (17.521) | 14.739 (20.000) | 0.9922 (W) | -1.24 |
| Lysophosphatidylcholine (lysoPC a C14:0) | 4.073 (0.259) | 3.891 (0.282) | < 0.0001 (W) | 1.71 |
| Phosphatidylcholine (PC aa C26:0) | 0.224 (0.017) | 0.214 (0.017) | < 0.0001 (W) | 1.72 |
| Phosphatidylcholine (PC aa C40:1) | 0.102 (0.015) | 0.096 (0.014) | < 0.0001 (W) | 1.77 |
| Phosphatidylcholine (PC ae C42:0) | 0.222 (0.017) | 0.222 (0.014) | < 0.0001 (W) | 1.64 |
