# Supplementary material for: Association of Metabolomic Biomarkers with Sleeve Gastrectomy Weight Loss Outcomes
Source: Metabolites. 2023 Mar 31;13(4):506. doi: 10.3390/metabo13040506 (PMC10145663; doi:10.3390/metabo13040506)
Supplement: Supplementary file 1 [file metabolites-13-00506-s001.zip › Supplementary Table 6.docx]

**Table S6:** Fecal Univariate analysis of Tertile 1 at three months post-sleeve gastrectomy compared with all patients at baseline (Mean Concentration: μM)

| **Metabolites** | **Mean (SD) of 3M** | **Mean (SD) of BL** | **P-value** | **Fold Change** |
| --- | --- | --- | --- | --- |
| 3-Hydroxyisovaleric acid | 51.548 (89.266) | 22.650 (52.540) | 0.0004 (W) | 2.7 |
| Isoleucine (Ile) | 248.620 (131.215) | 156.446 (74.682) | 0.0007 (W) | 1.95 |
| Isopropyl alcohol | 333.117 (88.525) | 265.294 (118.953) | 0.0026 | 1.41 |
| α-Aminoadipic acid (alpha-AAA) | 11.481 (11.720) | 15.210 (63.523) | 0.0071 (W) | -1.41 |
| Phosphatidylcholine (PC aa C40:1) | 0.109 (0.013) | 0.096 (0.014) | 0.016 (W) | 1.25 |
| Spermidine | 30.935 (86.914) | 74.220 (142.554) | 0.017 (W) | -2.75 |
| p-Cresol | 79.333 (33.282) | 62.799 (46.335) | 0.0218 (W) | 1.45 |
| Phosphatidylcholine (PC aa C42:6) | 0.048 (0.019) | 0.045 (0.012) | 0.039 (W) | 1.14 |
| Phosphatidylcholine (PC ae C34:2) | 0.060 (0.030) | 0.061 (0.070) | 0.0412 (W) | 1.07 |
| Phosphatidylcholine (PC a C24:0) | 0.102 (0.015) | 0.095 (0.016) | 0.0435 (W) | 1.14 |
| Sphingomyelin (SM (OH) C22:2) | 0.019 (0.010) | 0.015 (0.011) | 0.0511 (W) | 1.42 |
| N6-Acetyllysine | 96.185 (228.947) | 26.669 (23.594) | 0.0538 (W) | 5.56 |
| Malonate | 96.646 (29.928) | 84.650 (39.903) | 0.0557 | 1.28 |
| Methionine (Met) | 149.393 (86.910) | 121.675 (78.403) | 0.0628 (W) | 1.36 |
| Phosphatidylcholine (PC aa C36:0) | 0.185 (0.028) | 0.172 (0.023) | 0.0628 (W) | 1.17 |
| Phosphatidylcholine (PC aa C42:2) | 0.039 (0.007) | 0.038 (0.008) | 0.0628 (W) | 1.08 |
| Phosphatidylcholine (PC ae C32:2) | 0.029 (0.005) | 0.028 (0.005) | 0.066 (W) | 1.11 |
| Phosphatidylcholine (PC aa C40:2) | 0.094 (0.017) | 0.089 (0.012) | 0.0729 (W) | 1.13 |
| Phosphatidylcholine (PC ae C30:2) | 0.024 (0.004) | 0.023 (0.014) | 0.0843 (W) | 1.07 |
| Phosphatidylcholine (PC aa C40:5) | 0.028 (0.006) | 0.026 (0.007) | 0.0926 (W) | 1.13 |
| Phosphatidylcholine (PC ae C30:0) | 0.036 (0.009) | 0.034 (0.009) | 0.1063 (W) | 1.13 |
| Phosphatidylcholine (PC aa C38:5) | 0.051 (0.037) | 0.036 (0.024) | 0.1112 (W) | 1.49 |
| Phosphatidylcholine (PC ae C38:1) | 0.113 (0.021) | 0.103 (0.019) | 0.1112 (W) | 1.17 |
| Trimethylamine | 20.264 (27.048) | 11.341 (10.414) | 0.1112 (W) | 2.53 |
| Phosphatidylcholine (PC ae C36:0) | 0.040 (0.008) | 0.038 (0.006) | 0.1162 (W) | 1.14 |
| Methylamine | 31.377 (24.860) | 22.499 (10.226) | 0.1215 (W) | 1.5 |
| Decenoylcarnitine (C10:1) | 0.378 (0.151) | 0.320 (0.099) | 0.1269 (W) | 1.32 |
| Tiglylcarnitine (C5:1) | 0.039 (0.011) | 0.038 (0.030) | 0.1269 (W) | 1.09 |
| Aspartate (Asp) | 182.376 (59.987) | 170.479 (98.923) | 0.1383 (W) | 1.22 |
| Phosphatidylcholine (PC aa C42:0) | 0.063 (0.007) | 0.061 (0.009) | 0.1383 (W) | 1.1 |
| Phenylalanine (Phe) | 320.563 (128.721) | 286.409 (187.860) | 0.1383 (W) | 1.28 |
| L-Lactic acid | 56.347 (42.035) | 45.955 (28.103) | 0.1442 (W) | 1.55 |
| Phosphatidylcholine (PC aa C38:3) | 0.228 (0.032) | 0.220 (0.026) | 0.1442 (W) | 1.11 |
| L-Phenylalanine (Phe) | 130.893 (46.028) | 127.908 (54.929) | 0.1567 (W) | 1.13 |
| Phosphatidylcholine (PC aa C38:4) | 0.052 (0.013) | 0.051 (0.008) | 0.1567 (W) | 1.12 |
| Phosphatidylcholine (PC ae C36:2) | 0.037 (0.011) | 0.036 (0.015) | 0.1567 (W) | 1.11 |
| Phosphatidylcholine (PC ae C38:6) | 0.021 (0.007) | 0.021 (0.006) | 0.1567 (W) | 1.05 |
| Phosphatidylcholine (PC ae C42:0) | 0.231 (0.014) | 0.222 (0.014) | 0.1567 (W) | 1.14 |
| Asparagine (Asn) | 160.251 (98.443) | 133.509 (92.410) | 0.1633 (W) | 1.35 |
| Hydroxyoctadecenoylcarnitine (C18:1-OH) | 0.042 (0.077) | 0.052 (0.094) | 0.1633 (W) | -1.17 |
| Methanol | 0.992 (0.352) | 26.759 (158.031) | 0.1633 (W) | -19.65 |
| Phosphatidylcholine (PC aa C40:6) | 0.102 (0.019) | 0.099 (0.014) | 0.1633 (W) | 1.13 |
| Phosphatidylcholine (PC ae C38:4) | 0.047 (0.008) | 0.046 (0.008) | 0.1633 (W) | 1.09 |
| Sphingomyelin (SM C20:2) | 0.087 (0.018) | 0.088 (0.020) | 0.1633 (W) | 1.02 |
| Phosphatidylcholine (PC aa C36:3) | 0.612 (1.758) | 0.210 (0.750) | 0.17 (W) | 2.98 |
| Phosphatidylcholine (PC aa C38:6) | 0.032 (0.007) | 0.031 (0.006) | 0.17 (W) | 1.1 |
| Symmetric dimethylarginine | 0.481 (0.996) | 1.119 (3.859) | 0.177 (W) | -2.17 |
| Phosphatidylcholine (PC aa C36:5) | 0.029 (0.023) | 0.025 (0.022) | 0.1841 (W) | 1.2 |
| L-Fucose | 14.209 (7.663) | 28.281 (55.221) | 0.199 (W) | -1.8 |
| Phosphatidylcholine (PC aa C40:4) | 0.059 (0.006) | 0.059 (0.008) | 0.2068 (W) | 1.06 |
| Sphingomyelin (SM C18:1) | 0.014 (0.008) | 0.019 (0.036) | 0.2068 (W) | -1.18 |
| Octadecanoylcarnitine (C18) | 0.108 (0.098) | 0.145 (0.163) | 0.2148 (W) | -1.17 |
| Phosphatidylcholine (PC aa C28:1) | 0.026 (0.005) | 0.025 (0.006) | 0.2148 (W) | 1.11 |
| Phosphatidylcholine (PC ae C44:4) | 0.047 (0.006) | 0.048 (0.008) | 0.2148 (W) | 1.06 |
| Sphingomyelin (SM C26:0) | 0.007 (0.005) | 0.011 (0.006) | 0.2148 (W) | -1.4 |
| 2-Oxoglutarate | 40.452 (78.855) | 18.485 (13.293) | 0.223 (W) | 3.27 |
| Hydroxyhexadecenoylcarnitine (C16:1-OH) | 0.189 (0.127) | 0.181 (0.185) | 0.223 (W) | 1.14 |
| Pimelylcarnitine (C7-DC) | 0.035 (0.030) | 0.035 (0.042) | 0.223 (W) | 1.04 |
| Formate | 35.374 (8.971) | 34.777 (18.607) | 0.223 (W) | 1.17 |
| Lysophosphatidylcholine (lysoPC a C20:4) | 0.037 (0.012) | 0.062 (0.066) | 0.223 (W) | -1.62 |
| Taurine | 39.216 (103.086) | 56.947 (91.191) | 0.223 (W) | -1.08 |
| Acetone | 334.491 (155.562) | 311.244 (160.845) | 0.2244 | 1.18 |
| Choline | 3.111 (2.208) | 4.891 (4.750) | 0.2314 (W) | -1.34 |
| Phosphatidylcholine (PC ae C42:5) | 0.281 (0.022) | 0.276 (0.023) | 0.2314 (W) | 1.12 |
| Lysophosphatidylcholine (lysoPC a C18:2) | 0.872 (1.795) | 0.776 (1.809) | 0.24 (W) | 1.08 |
| Phosphatidylcholine (PC ae C40:5) | 0.059 (0.008) | 0.058 (0.009) | 0.24 (W) | 1.1 |
| 3-Hydroxybutyric acid | 14.680 (18.275) | 11.786 (13.525) | 0.2489 (W) | 1.55 |
| Hydroxyhexadecanoylcarnitine (C16-OH) | 0.182 (0.150) | 0.163 (0.180) | 0.2489 (W) | 1.21 |
| Phosphatidylcholine (PC aa C32:3) | 0.037 (0.006) | 0.037 (0.007) | 0.2489 (W) | 1.08 |
| Phosphatidylcholine (PC ae C40:3) | 0.098 (0.013) | 0.098 (0.017) | 0.2489 (W) | 1.08 |
| trans -4-Hydroxyprolinet4-OH-Pro | 2.191 (3.100) | 7.385 (27.371) | 0.2489 (W) | -3.24 |
| Butenoylcarnitine (C4:1) | 0.056 (0.021) | 0.050 (0.015) | 0.2579 (W) | 1.23 |
| Phosphatidylcholine (PC aa C42:4) | 0.044 (0.003) | 0.044 (0.008) | 0.2579 (W) | 1.09 |
| Sphingomyelin (SM (OH) C24:1) | 0.012 (0.004) | 0.012 (0.006) | 0.2579 (W) | 1.19 |
| Octadecenoylcarnitine (C18:1) | 0.044 (0.038) | 0.095 (0.127) | 0.2672 (W) | -2.03 |
| Hydroxyhexanoylcarnitine (C5-DC (C6-OH) | 0.034 (0.008) | 0.033 (0.010) | 0.2672 (W) | 1.15 |
| D-Galactose | 48.921 (18.036) | 75.359 (173.282) | 0.2672 (W) | -1.29 |
| Phosphatidylcholine (PC ae C42:2) | 0.044 (0.007) | 0.043 (0.006) | 0.2672 (W) | 1.09 |
| Hydroxybutyrylcarnitine (C3-DC (C4-OH) | 0.149 (0.089) | 0.135 (0.085) | 0.2767 (W) | 1.21 |
| Phosphatidylcholine (PC ae C34:0) | 0.049 (0.008) | 0.048 (0.009) | 0.2767 (W) | 1.12 |
| Sphingomyelin (SM C24:0) | 0.081 (0.013) | 0.093 (0.074) | 0.2767 (W) | -1.01 |
| Phosphatidylcholine (PC aa C36:4) | 0.302 (0.719) | 0.325 (1.526) | 0.2864 (W) | -1.03 |
| Phosphatidylcholine PC ae C44:5) | 0.031 (0.004) | 0.031 (0.007) | 0.2864 (W) | 1.06 |
| Phosphatidylcholine (PC aa C34:3) | 0.026 (0.026) | 0.023 (0.028) | 0.2964 (W) | 1.2 |
| Phosphatidylcholine (PC aa C36:1) | 0.069 (0.038) | 0.073 (0.070) | 0.2964 (W) | 1.11 |
| Phosphatidylcholine (PC ae C34:3) | 0.038 (0.010) | 0.040 (0.022) | 0.2964 (W) | 1.01 |
| Serotonin | 0.972 (1.940) | 0.447 (0.515) | 0.2964 (W) | 2.35 |
| Threonine (Thr) | 233.265 (142.352) | 222.194 (104.833) | 0.2969 | 1.16 |
| Ornithine (Orn) | 85.770 (66.158) | 69.299 (54.923) | 0.2998 | 1.25 |
| Lysophosphatidylcholine (lysoPC a C14:0) | 3.897 (0.284) | 3.891 (0.282) | 0.3065 (W) | 1.1 |
| Phenylacetate | 87.983 (47.712) | 81.509 (47.041) | 0.3127 | 1.18 |
| Caprylate | 78.746 (64.917) | 86.167 (204.573) | 0.3169 (W) | 1.12 |
| Phosphatidylcholine (PC ae C38:5 | 0.047 (0.005) | 0.048 (0.009) | 0.3169 (W) | 1.07 |
| Methylglutarylcarnitine (C5-M-DC) | 0.036 (0.015) | 0.035 (0.011) | 0.3262 | 1.13 |
| Phosphatidylcholine (PC ae C38:0) | 0.023 (0.002) | 0.024 (0.004) | 0.3276 (W) | 1.03 |
| Phosphatidylcholine (PC ae C40:2) | 0.073 (0.011) | 0.069 (0.012) | 0.3276 (W) | 1.13 |
| Phosphatidylcholine (PC ae C42:1) | 0.043 (0.005) | 0.043 (0.009) | 0.3276 (W) | 1.06 |
| Betaine | 3.878 (2.555) | 10.000 (13.176) | 0.3494 (W) | -2.23 |
| Acetic acid | 1201.740 (643.431) | 1410.917 (510.929) | 0.356 | -1.12 |
| Butyrate | 817.889 (869.377) | 862.093 (616.086) | 0.3607 (W) | -1.06 |
| Phosphatidylcholine (PC aa C36:6) | 0.017 (0.003) | 0.018 (0.010) | 0.3607 (W) | 1.06 |
| Phosphatidylcholine (PC a C26:1) | 0.031 (0.008) | 0.030 (0.010) | 0.3722 (W) | 1.07 |
| Lysophosphatidylcholine (lysoPC a C28:1) | 0.049 (0.011) | 0.048 (0.012) | 0.3722 (W) | 1.13 |
| N-Acetylglutamate | 22.655 (18.247) | 20.095 (13.888) | 0.3722 (W) | 1.44 |
| Lysophosphatidylcholine (lysoPC a C16:1) | 0.066 (0.039) | 0.080 (0.040) | 0.3839 (W) | -1.1 |
| Phosphatidylcholine (PC aa C40:3) | 0.192 (0.026) | 0.191 (0.022) | 0.3839 (W) | 1.09 |
| Phosphatidylcholine (PC aa C42:1) | 0.042 (0.006) | 0.041 (0.006) | 0.3839 (W) | 1.11 |
| 1-Methylhistidine | 18.063 (27.061) | 15.083 (10.662) | 0.3959 (W) | 1.63 |
| Glutamine (Gln) | 92.489 (61.721) | 98.243 (101.024) | 0.3959 (W) | 1.11 |
| Histidine (His) | 17.649 (26.511) | 23.052 (27.933) | 0.3959 (W) | -1.32 |
| Phosphatidylcholine (PC aa C24:0) | 0.038 (0.007) | 0.039 (0.014) | 0.3959 (W) | -1.02 |
| Phosphatidylcholine (PC ae C40:1) | 0.050 (0.009) | 0.049 (0.007) | 0.3959 (W) | 1.11 |
| Pyruvic acid | 9.968 (7.127) | 8.401 (5.789) | 0.3959 (W) | 1.34 |
| Acetylcarnitine (C2) | 0.067 (0.018) | 0.107 (0.213) | 0.408 (W) | -1.5 |
| Lysophosphatidylcholine (lysoPC a C17:0) | 0.114 (0.018) | 0.128 (0.073) | 0.408 (W) | -1.03 |
| Phosphatidylcholine (PC ae C40:4) | 0.037 (0.006) | 0.038 (0.008) | 0.408 (W) | 1.07 |
| Arabinose | 84.620 (59.931) | 81.820 (69.114) | 0.4204 (W) | 1.16 |
| Isovaleric acid | 2.209 (2.344) | 5.550 (14.047) | 0.4204 (W) | -2.36 |
| Phosphatidylcholine (PC a C26:0) | 0.120 (0.019) | 0.120 (0.014) | 0.4204 (W) | 1.11 |
| Phosphatidylcholine (PC ae C30:1) | 0.016 (0.005) | 0.017 (0.003) | 0.4204 (W) | 1.06 |
| Phosphatidylcholine (PC ae C40:6 | 0.033 (0.006) | 0.033 (0.006) | 0.4204 (W) | 1.06 |
| Tetradecanoylcarnitine (C14) | 0.034 (0.009) | 0.049 (0.034) | 0.4329 (W) | -1.3 |
| Butyrylcarnitine (C4) | 0.027 (0.013) | 0.039 (0.031) | 0.4329 (W) | -1.24 |
| Hydroxyvalerylcarnitine (C5-OH (C3-DC-M)) | 0.051 (0.033) | 0.073 (0.059) | 0.4329 (W) | -1.25 |
| D-Glucose | 266.971 (230.362) | 425.231 (376.520) | 0.4329 (W) | -1.53 |
| L-Lysine (Lys) | 206.939 (86.084) | 224.705 (186.741) | 0.4329 (W) | 1.05 |
| Phosphatidylcholine (PC ae C42:3) | 0.051 (0.008) | 0.050 (0.008) | 0.4329 (W) | 1.09 |
| Dodecanoylcarnitine (C12:1) | 0.424 (0.178) | 0.393 (0.144) | 0.4457 (W) | 1.2 |
| Lysophosphatidylcholine (lysoPC a C16:0) | 1.664 (2.581) | 2.213 (3.234) | 0.4457 (W) | -1.31 |
| Phosphatidylcholine (PC aa C34:4) | 0.024 (0.005) | 0.024 (0.004) | 0.4457 (W) | 1.11 |
| Phosphatidylcholine (PC ae C36:4) | 0.066 (0.012) | 0.070 (0.023) | 0.4457 (W) | 1.02 |
| Dodecanoylcarnitine (C12) | 0.066 (0.025) | 0.067 (0.036) | 0.4587 (W) | 1.15 |
| Propionylcarnitine (C3) | 0.032 (0.018) | 0.032 (0.016) | 0.4587 (W) | 1.08 |
| Tetradecenoylcarnitine (C14:1) | 0.015 (0.006) | 0.016 (0.015) | 0.4719 (W) | 1.09 |
| Phosphatidylcholine (PC a C20:3) | 0.112 (0.021) | 0.117 (0.027) | 0.4754 | 1.07 |
| Nonanoylcarnitine (C9) | 0.025 (0.016) | 0.031 (0.026) | 0.4853 (W) | -1.07 |
| Phosphatidylcholine (PC aa C42:5) | 0.043 (0.005) | 0.044 (0.008) | 0.4853 (W) | 1.05 |
| Phosphatidylcholine (PC ae C36:5) | 0.033 (0.006) | 0.033 (0.007) | 0.4853 (W) | 1.1 |
| Phosphatidylcholine (PC ae C44:3) | 0.037 (0.006) | 0.037 (0.008) | 0.4853 (W) | 1.07 |
| Proline (Pro) | 256.836 (150.015) | 261.914 (137.346) | 0.4902 | 1.13 |
| Glycine (Gly) | 536.540 (276.723) | 556.189 (323.615) | 0.4989 (W) | 1.07 |
| Phosphatidylcholine (PC ae C36:3) | 0.042 (0.007) | 0.046 (0.019) | 0.4989 (W) | -1.03 |
| Hypoxanthine | 53.580 (33.846) | 52.856 (31.070) | 0.5057 | 1.13 |
| Lysophosphatidylcholine (lysoPC a C18:0) | 0.666 (0.117) | 1.240 (1.756) | 0.5126 (W) | -1.63 |
| N-Acetylcysteine | 23.355 (11.717) | 22.976 (10.849) | 0.5126 (W) | 1.16 |
| L-Glutamic acid | 897.180 (355.520) | 905.123 (495.406) | 0.5164 | 1.1 |
| 4-Hydroxyphenylacetate | 9.273 (3.105) | 10.599 (7.280) | 0.5266 (W) | -1.07 |
| Phosphatidylcholine (PC aa C26:0) | 0.213 (0.018) | 0.214 (0.017) | 0.5266 (W) | 1.12 |
| Phosphatidylcholine (PC aa C36:2) | 0.463 (1.200) | 0.202 (0.586) | 0.5408 (W) | 2.43 |
| Propionate | 1419.941 (763.434) | 1379.153 (679.798) | 0.5408 (W) | 1.06 |
| Serine (Ser) | 221.205 (109.321) | 255.905 (205.735) | 0.5408 (W) | 1.05 |
| Phosphatidylcholine (PC aa C34:1) | 0.556 (0.956) | 0.368 (0.404) | 0.5551 (W) | 1.6 |
| Methionine sulfoxide (Met-SO) | 22.578 (16.040) | 23.983 (23.341) | 0.5696 (W) | 1.1 |
| Nicotinate | 24.850 (27.693) | 20.277 (12.557) | 0.5696 (W) | 1.65 |
| Phosphatidylcholine (PC aa C30:0) | 0.071 (0.025) | 0.096 (0.052) | 0.5696 (W) | -1.21 |
| Sphingomyelin (SM (OH) C22:1) | 0.021 (0.009) | 0.032 (0.066) | 0.5696 (W) | -1.25 |
| Acetoacetate | 10.479 (5.179) | 17.594 (34.709) | 0.5992 (W) | -1.31 |
| Hexenoylcarnitine (C6:1) | 0.036 (0.013) | 0.035 (0.009) | 0.5992 (W) | 1.14 |
| Phosphatidylcholine (PC aa C34:2) | 0.370 (0.756) | 0.280 (0.617) | 0.6142 (W) | 1.37 |
| L-Alanine (Ala) | 1109.232 (716.920) | 1145.361 (570.460) | 0.6152 | 1.07 |
| 3-Phenylpropionate | 26.741 (15.148) | 32.410 (19.280) | 0.6294 (W) | -1.05 |
| Sphingomyelin (SM C16:0) | 0.360 (0.357) | 0.715 (1.266) | 0.6294 (W) | -1.73 |
| Valine (Val) | 10.456 (11.800) | 18.462 (57.771) | 0.6294 (W) | -1.46 |
| Ethanol | 49.232 (25.333) | 119.934 (237.995) | 0.6447 (W) | -1.96 |
| Valerylcarnitine (C5) | 0.034 (0.008) | 0.047 (0.042) | 0.6602 (W) | -1.25 |
| Phosphatidylcholine (PC aa C32:2) | 0.026 (0.005) | 0.027 (0.011) | 0.6602 (W) | 1.06 |
| Acetylornithine (Ac-Orn) | 200.958 (658.729) | 496.622 (1348.812) | 0.6759 (W) | -3.1 |
| Hydroxytetradecenoylcarnitine (C14:1-OH) | 0.026 (0.016) | 0.033 (0.025) | 0.6759 (W) | -1.12 |
|  | 28.201 (31.398) | 20.844 (14.157) | 0.6759 (W) | 1.78 |
| L-Leucine (Leu) | 78.072 (95.518) | 209.266 (631.389) | 0.6759 (W) | -2.15 |
| Phosphatidylcholine (PC ae C44:6) | 0.040 (0.007) | 0.041 (0.006) | 0.6759 (W) | 1.04 |
| Hydroxytetradecadienylcarnitine (C14:2-OH) | 0.016 (0.006) | 0.019 (0.009) | 0.6916 (W) | -1.04 |
| Phosphatidylcholine (PC aa C38:0) | 0.038 (0.006) | 0.039 (0.007) | 0.6916 (W) | 1.04 |
| Sarcosine | 47.224 (30.766) | 139.453 (270.882) | 0.6916 (W) | -2.88 |
| Lysophosphatidylcholine (lysoPC a C18:1) | 1.383 (3.328) | 0.630 (0.922) | 0.7075 (W) | 2.1 |
| Phosphatidylcholine (PC a C28:0) | 0.125 (0.022) | 0.129 (0.015) | 0.7075 (W) | 1.06 |
| Phosphatidylcholine (PC aa C32:0) | 0.182 (0.025) | 0.213 (0.089) | 0.7075 (W) | -1.05 |
| Sphingomyelin (SM C16:1) | 0.043 (0.009) | 0.049 (0.023) | 0.7075 (W) | -1.03 |
| Citrulline (Cit) | 217.699 (153.732) | 251.796 (143.999) | 0.7101 | -1.07 |
| Tyrosine (Tyr) | 269.662 (125.737) | 284.217 (173.176) | 0.717 | 1.06 |
| Propenoylcarnitine (C3:1) | 0.017 (0.009) | 0.018 (0.009) | 0.7235 (W) | 1.02 |
| Hydroxypropionylcarnitine (C3-OH) | 0.018 (0.007) | 0.017 (0.006) | 0.7235 (W) | 1.22 |
| Dimethylamine | 6.656 (8.131) | 5.343 (5.813) | 0.7235 (W) | 1.63 |
| Glutamate (Glu) | 926.128 (299.328) | 1118.856 (557.396) | 0.7296 | -1.05 |
| Phosphatidylcholine (PC ae C30:1) | 0.009 (0.003) | 0.010 (0.004) | 0.7397 (W) | 1.03 |
| Phosphatidylcholine (PC ae C38:2) | 0.046 (0.010) | 0.049 (0.017) | 0.7397 (W) | -1.01 |
| Tryptophan (Trp) | 42.706 (22.610) | 45.405 (27.370) | 0.7397 (W) | 1.04 |
| Urocanate | 12.136 (9.179) | 14.311 (8.521) | 0.7397 (W) | -1.07 |
| Dodecanedioylcarnitine (C12-DC) | 0.073 (0.022) | 0.077 (0.024) | 0.7559 (W) | 1.09 |
| Phosphatidylcholine (PC ae C34:1) | 0.043 (0.028) | 0.057 (0.062) | 0.7723 (W) | -1.19 |
| Putrescine | 10.389 (13.836) | 14.710 (20.021) | 0.7723 (W) | -1.05 |
| Octanoylcarnitine (C8) | 0.065 (0.019) | 0.067 (0.013) | 0.7888 (W) | 1.08 |
| Phosphatidylcholine (PC ae C38:3) | 0.082 (0.012) | 0.087 (0.020) | 0.7888 (W) | 1.03 |
| Decanoylcarnitine (C10) | 0.056 (0.022) | 0.058 (0.020) | 0.822 (W) | 1.12 |
| Creatine | 48.827 (21.367) | 55.438 (30.345) | 0.822 (W) | -1.04 |
| L-Arginine (Arg) | 85.251 (261.795) | 78.894 (226.042) | 0.822 (W) | 1.11 |
| Octadecadienylcarnitine (C18:2) | 0.028 (0.026) | 0.041 (0.051) | 0.8387 (W) | -1.3 |
| Fumarylcarnitine (C6 (C4:1-DC)) | 0.040 (0.015) | 0.045 (0.013) | 0.8409 | -1.02 |
| Sphingomyelin (SM (OH) C16:1) | 0.014 (0.008) | 0.028 (0.044) | 0.8555 (W) | -1.54 |
| Thymine | 42.664 (21.925) | 46.416 (23.188) | 0.8591 | -1.03 |
| Decenoylcarnitine (C10:2) | 0.054 (0.038) | 0.065 (0.061) | 0.8723 (W) | -1.14 |
| 4-Hydroxyphenyllactate | 14.058 (7.108) | 22.884 (30.009) | 0.9062 (W) | -1.49 |
| Carnitine (C0) | 3.108 (1.060) | 3.925 (2.393) | 0.9062 (W) | -1.1 |
| Hydroxyhexadecadienylcarnitine (C16:2-OH) | 0.024 (0.010) | 0.029 (0.023) | 0.9062 (W) | -1.07 |
| Isobutyric acid | 741.321 (148.583) | 1029.718 (1542.053) | 0.9062 (W) | -1.17 |
| Uracil | 122.506 (64.225) | 132.057 (65.064) | 0.9169 | -1.02 |
| Tetradecadienylcarnitine (C14:2) | 0.017 (0.006) | 0.021 (0.013) | 0.9232 (W) | -1.04 |
| Hexadecadienylcarnitine (C16:2) | 0.019 (0.008) | 0.024 (0.022) | 0.9232 (W) | -1.14 |
| Creatinine | 52.914 (91.371) | 45.343 (87.702) | 0.9232 (W) | 1.47 |
| Phosphatidylcholine (PC aa C32:1) | 0.027 (0.015) | 0.034 (0.025) | 0.9232 (W) | -1.15 |
| Sphingomyelin (SM C18:0) | 0.085 (0.134) | 0.179 (0.312) | 0.9232 (W) | -2 |
| Phosphatidylcholine (PC ae C36:1) | 0.087 (0.015) | 0.099 (0.035) | 0.9344 | -1.01 |
| Hexadecanoylcarnitine (C16) | 0.073 (0.058) | 0.133 (0.170) | 0.9402 (W) | -1.69 |
| Succinate | 76.960 (72.251) | 273.788 (739.267) | 0.9402 (W) | -2.51 |
| Glutaconylcarnitine (C5:1-DC) | 0.090 (0.058) | 0.100 (0.036) | 0.9417 | -1.01 |
| Valerate | 228.740 (120.633) | 239.750 (152.895) | 0.9425 | 1.01 |
| Xanthine | 1.468 (1.766) | 5.385 (25.405) | 0.9573 (W) | -2.8 |
| Lysophosphatidylcholine (lysoPC a C16:1) | 0.030 (0.019) | 0.041 (0.044) | 0.9744 (W) | -1.21 |
| Sphingomyelin (SM (OH) C14:1) | 0.015 (0.007) | 0.023 (0.031) | 1 | -1.42 |
