# Supplementary material for: Association of Metabolomic Biomarkers with Sleeve Gastrectomy Weight Loss Outcomes
Source: Metabolites. 2023 Mar 31;13(4):506. doi: 10.3390/metabo13040506 (PMC10145663; doi:10.3390/metabo13040506)
Supplement: Supplementary file 1 [file metabolites-13-00506-s001.zip › Supplementary Table 7.docx]

**Table S7:** Fecal Metabolite Set Enrichment Analysis of all tertiles at three months post-sleeve gastrectomy compared with all patients at baseline.

| Pathway | Total Cmpd | Hits | P-Value | Holm p | FDR |
| --- | --- | --- | --- | --- | --- |
| Arachidonic Acid Metabolism | 69 | 3 | 0.002199 | 0.15831 | 0.15831 |
| Tyrosine Metabolism | 72 | 6 | 0.01814 | 1 | 0.2461 |
| Glycolysis | 25 | 2 | 0.030004 | 1 | 0.2461 |
| Transfer of Acetyl Groups into Mitochondria | 22 | 2 | 0.030004 | 1 | 0.2461 |
| Pyruvaldehyde Degradation | 10 | 1 | 0.033311 | 1 | 0.2461 |
| Galactose Metabolism | 38 | 2 | 0.036537 | 1 | 0.2461 |
| Lactose Degradation | 9 | 2 | 0.036537 | 1 | 0.2461 |
| Fatty Acid Biosynthesis | 35 | 6 | 0.038807 | 1 | 0.2461 |
| Nucleotide Sugars Metabolism | 20 | 1 | 0.04241 | 1 | 0.2461 |
| Pterine Biosynthesis | 29 | 1 | 0.05152 | 1 | 0.2461 |
| Steroid Biosynthesis | 48 | 1 | 0.05152 | 1 | 0.2461 |
| Androgen and Estrogen Metabolism | 33 | 1 | 0.05152 | 1 | 0.2461 |
| Androstenedione Metabolism | 24 | 1 | 0.05152 | 1 | 0.2461 |
| Mitochondrial Beta-Oxidation of Short Chain Saturated Fatty Acids | 27 | 3 | 0.054259 | 1 | 0.2461 |
| Bile Acid Biosynthesis | 65 | 2 | 0.054565 | 1 | 0.2461 |
| Pyruvate Metabolism | 48 | 3 | 0.05469 | 1 | 0.2461 |
| Gluconeogenesis | 35 | 3 | 0.064966 | 1 | 0.27397 |
| Porphyrin Metabolism | 40 | 1 | 0.068491 | 1 | 0.27397 |
| Alanine Metabolism | 17 | 4 | 0.089816 | 1 | 0.32558 |
| Ketone Body Metabolism | 13 | 4 | 0.099093 | 1 | 0.32558 |
| Amino Sugar Metabolism | 33 | 4 | 0.10086 | 1 | 0.32558 |
| Cysteine Metabolism | 26 | 2 | 0.10091 | 1 | 0.32558 |
| Phospholipid Biosynthesis | 29 | 3 | 0.10915 | 1 | 0.32558 |
| Lactose Synthesis | 20 | 1 | 0.11166 | 1 | 0.32558 |
| Glucose-Alanine Cycle | 13 | 4 | 0.1131 | 1 | 0.32558 |
| Taurine and Hypotaurine Metabolism | 12 | 1 | 0.11757 | 1 | 0.32558 |
| Sphingolipid Metabolism | 40 | 4 | 0.12583 | 1 | 0.3335 |
| Folate Metabolism | 29 | 2 | 0.13672 | 1 | 0.3335 |
| Citric Acid Cycle | 32 | 3 | 0.14082 | 1 | 0.3335 |
| Betaine Metabolism | 21 | 3 | 0.14337 | 1 | 0.3335 |
| Ammonia Recycling | 32 | 8 | 0.14359 | 1 | 0.3335 |
| Glutamate Metabolism | 49 | 7 | 0.16737 | 1 | 0.36604 |
| Methylhistidine Metabolism | 4 | 1 | 0.16777 | 1 | 0.36604 |
| Threonine and 2-Oxobutanoate Degradation | 20 | 1 | 0.18475 | 1 | 0.38008 |
| Glycine and Serine Metabolism | 59 | 12 | 0.18817 | 1 | 0.38008 |
| Methionine Metabolism | 43 | 9 | 0.19004 | 1 | 0.38008 |
| Spermidine and Spermine Biosynthesis | 18 | 4 | 0.21853 | 1 | 0.42525 |
| Ethanol Degradation | 19 | 2 | 0.22531 | 1 | 0.4269 |
| Tryptophan Metabolism | 60 | 5 | 0.23776 | 1 | 0.43893 |
| Glutathione Metabolism | 21 | 3 | 0.25995 | 1 | 0.46792 |
| Carnitine Synthesis | 22 | 4 | 0.27328 | 1 | 0.47241 |
| Warburg Effect | 58 | 7 | 0.27557 | 1 | 0.47241 |
| Fructose and Mannose Degradation | 32 | 1 | 0.30287 | 1 | 0.50714 |
| Purine Metabolism | 74 | 7 | 0.31708 | 1 | 0.51886 |
| Phosphatidylcholine Biosynthesis | 14 | 1 | 0.34503 | 1 | 0.54653 |
| Beta Oxidation of Very Long Chain Fatty Acids | 17 | 3 | 0.34917 | 1 | 0.54653 |
| Malate-Aspartate Shuttle | 10 | 2 | 0.37092 | 1 | 0.56033 |
| Fatty acid Metabolism | 43 | 2 | 0.38312 | 1 | 0.56033 |
| Beta-Alanine Metabolism | 34 | 4 | 0.38489 | 1 | 0.56033 |
| Mitochondrial Beta-Oxidation of Long Chain Saturated Fatty Acids | 28 | 2 | 0.38912 | 1 | 0.56033 |
| Lysine Degradation | 30 | 3 | 0.41111 | 1 | 0.58039 |
| Biotin Metabolism | 8 | 1 | 0.4271 | 1 | 0.59137 |
| Urea Cycle | 29 | 9 | 0.44747 | 1 | 0.60788 |
| Homocysteine Degradation | 9 | 1 | 0.48119 | 1 | 0.63585 |
| Aspartate Metabolism | 35 | 8 | 0.48572 | 1 | 0.63585 |
| Phosphatidylethanolamine Biosynthesis | 12 | 2 | 0.50039 | 1 | 0.64336 |
| Oxidation of Branched Chain Fatty Acids | 26 | 4 | 0.53195 | 1 | 0.67194 |
| Phenylacetate Metabolism | 9 | 1 | 0.56516 | 1 | 0.70158 |
| Mitochondrial Electron Transport Chain | 19 | 2 | 0.62139 | 1 | 0.75586 |
| Selenoamino Acid Metabolism | 28 | 2 | 0.62988 | 1 | 0.75586 |
| Catecholamine Biosynthesis | 20 | 1 | 0.65265 | 1 | 0.75792 |
| Thyroid hormone synthesis | 13 | 1 | 0.65265 | 1 | 0.75792 |
| Vitamin K Metabolism | 14 | 1 | 0.67935 | 1 | 0.76455 |
| Arginine and Proline Metabolism | 53 | 10 | 0.69158 | 1 | 0.76455 |
| Valine, Leucine and Isoleucine Degradation | 60 | 6 | 0.69867 | 1 | 0.76455 |
| Histidine Metabolism | 43 | 4 | 0.70122 | 1 | 0.76455 |
| Pyrimidine Metabolism | 59 | 3 | 0.71146 | 1 | 0.76455 |
| Butyrate Metabolism | 19 | 3 | 0.81386 | 1 | 0.86174 |
| Phenylalanine and Tyrosine Metabolism | 28 | 5 | 0.86157 | 1 | 0.89903 |
| Nicotinate and Nicotinamide Metabolism | 37 | 3 | 0.90144 | 1 | 0.9272 |
| Phytanic Acid Peroxisomal Oxidation | 26 | 1 | 0.92005 | 1 | 0.93301 |
| Propanoate Metabolism | 42 | 3 | 0.9413 | 1 | 0.9413 |
