# Supplementary material for: Association of Metabolomic Biomarkers with Sleeve Gastrectomy Weight Loss Outcomes
Source: Metabolites. 2023 Mar 31;13(4):506. doi: 10.3390/metabo13040506 (PMC10145663; doi:10.3390/metabo13040506)
Supplement: Supplementary file 1 [file metabolites-13-00506-s001.zip › Supplementary Table 8.docx]

**Table S8:** Fecal Metabolite Set Enrichment Analysis of Tertile 1 at three months post-sleeve gastrectomy compared with all patients at baseline.

| **Pathway** | **Total Cmpd** | **Hits** | **Raw p** | **Holm p** | **FDR** |
| --- | --- | --- | --- | --- | --- |
| Porphyrin Metabolism | 40 | 1 | 0.014472 | 1 | 0.562 |
| Bile Acid Biosynthesis | 65 | 2 | 0.034948 | 1 | 0.562 |
| Methionine Metabolism | 43 | 9 | 0.036661 | 1 | 0.562 |
| Betaine Metabolism | 21 | 3 | 0.053321 | 1 | 0.562 |
| Ammonia Recycling | 32 | 8 | 0.060519 | 1 | 0.562 |
| Alanine Metabolism | 17 | 4 | 0.073695 | 1 | 0.562 |
| Glutamate Metabolism | 49 | 7 | 0.079808 | 1 | 0.562 |
| Glutathione Metabolism | 21 | 3 | 0.091431 | 1 | 0.562 |
| Lysine Degradation | 30 | 3 | 0.095039 | 1 | 0.562 |
| Pyruvate Metabolism | 48 | 3 | 0.12336 | 1 | 0.562 |
| Pyruvaldehyde Degradation | 10 | 1 | 0.12979 | 1 | 0.562 |
| Purine Metabolism | 74 | 7 | 0.12997 | 1 | 0.562 |
| Phosphatidylcholine Biosynthesis | 14 | 1 | 0.13046 | 1 | 0.562 |
| Carnitine Synthesis | 22 | 4 | 0.13908 | 1 | 0.562 |
| Gluconeogenesis | 35 | 3 | 0.14098 | 1 | 0.562 |
| Glycine and Serine Metabolism | 59 | 12 | 0.15216 | 1 | 0.562 |
| Valine, Leucine and Isoleucine Degradation | 60 | 6 | 0.16064 | 1 | 0.562 |
| Glycolysis | 25 | 2 | 0.17928 | 1 | 0.562 |
| Transfer of Acetyl Groups into Mitochondria | 22 | 2 | 0.17928 | 1 | 0.562 |
| Phenylacetate Metabolism | 9 | 1 | 0.18905 | 1 | 0.562 |
| Amino Sugar Metabolism | 33 | 4 | 0.19377 | 1 | 0.562 |
| Spermidine and Spermine Biosynthesis | 18 | 4 | 0.20883 | 1 | 0.562 |
| Pterine Biosynthesis | 29 | 1 | 0.20937 | 1 | 0.562 |
| Steroid Biosynthesis | 48 | 1 | 0.20937 | 1 | 0.562 |
| Androgen and Estrogen Metabolism | 33 | 1 | 0.20937 | 1 | 0.562 |
| Androstenedione Metabolism | 24 | 1 | 0.20937 | 1 | 0.562 |
| Malate-Aspartate Shuttle | 10 | 2 | 0.21075 | 1 | 0.562 |
| Cysteine Metabolism | 26 | 2 | 0.22293 | 1 | 0.57095 |
| Aspartate Metabolism | 35 | 8 | 0.22997 | 1 | 0.57095 |
| Phosphatidylethanolamine Biosynthesis | 12 | 2 | 0.27336 | 1 | 0.61909 |
| Fatty Acid Biosynthesis | 35 | 6 | 0.27765 | 1 | 0.61909 |
| Nicotinate and Nicotinamide Metabolism | 37 | 3 | 0.2848 | 1 | 0.61909 |
| Warburg Effect | 58 | 7 | 0.28732 | 1 | 0.61909 |
| Lactose Synthesis | 20 | 1 | 0.29235 | 1 | 0.61909 |
| Tyrosine Metabolism | 72 | 6 | 0.31763 | 1 | 0.65342 |
| Folate Metabolism | 29 | 2 | 0.33187 | 1 | 0.66374 |
| Biotin Metabolism | 8 | 1 | 0.34776 | 1 | 0.67364 |
| Glucose-Alanine Cycle | 13 | 4 | 0.37114 | 1 | 0.67364 |
| Taurine and Hypotaurine Metabolism | 12 | 1 | 0.37227 | 1 | 0.67364 |
| Arachidonic Acid Metabolism | 69 | 3 | 0.38303 | 1 | 0.67364 |
| Arginine and Proline Metabolism | 53 | 10 | 0.38684 | 1 | 0.67364 |
| Citric Acid Cycle | 32 | 3 | 0.39902 | 1 | 0.67364 |
| Galactose Metabolism | 38 | 2 | 0.41721 | 1 | 0.67364 |
| Lactose Degradation | 9 | 2 | 0.41721 | 1 | 0.67364 |
| Phospholipid Biosynthesis | 29 | 3 | 0.42215 | 1 | 0.67364 |
| Urea Cycle | 29 | 9 | 0.43747 | 1 | 0.67364 |
| Nucleotide Sugars Metabolism | 20 | 1 | 0.43974 | 1 | 0.67364 |
| Ethanol Degradation | 19 | 2 | 0.47133 | 1 | 0.7006 |
| Ketone Body Metabolism | 13 | 4 | 0.4768 | 1 | 0.7006 |
| Beta-Alanine Metabolism | 34 | 4 | 0.49774 | 1 | 0.70323 |
| Tryptophan Metabolism | 60 | 5 | 0.49812 | 1 | 0.70323 |
| Threonine and 2-Oxobutanoate Degradation | 20 | 1 | 0.53171 | 1 | 0.73621 |
| Histidine Metabolism | 43 | 4 | 0.5506 | 1 | 0.74798 |
| Pyrimidine Metabolism | 59 | 3 | 0.56482 | 1 | 0.74949 |
| Homocysteine Degradation | 9 | 1 | 0.57253 | 1 | 0.74949 |
| Fructose and Mannose Degradation | 32 | 1 | 0.59899 | 1 | 0.77013 |
| Methylhistidine Metabolism | 4 | 1 | 0.61877 | 1 | 0.7816 |
| Sphingolipid Metabolism | 40 | 4 | 0.67529 | 1 | 0.83829 |
| Mitochondrial Beta-Oxidation of Short Chain Saturated Fatty Acids | 27 | 3 | 0.70731 | 1 | 0.84607 |
| Mitochondrial Electron Transport Chain | 19 | 2 | 0.72041 | 1 | 0.84607 |
| Phytanic Acid Peroxisomal Oxidation | 26 | 1 | 0.72156 | 1 | 0.84607 |
| Mitochondrial Beta-Oxidation of Long Chain Saturated Fatty Acids | 28 | 2 | 0.72856 | 1 | 0.84607 |
| Beta Oxidation of Very Long Chain Fatty Acids | 17 | 3 | 0.77046 | 1 | 0.88053 |
| Selenoamino Acid Metabolism | 28 | 2 | 0.80467 | 1 | 0.89589 |
| Fatty acid Metabolism | 43 | 2 | 0.81106 | 1 | 0.89589 |
| Catecholamine Biosynthesis | 20 | 1 | 0.83368 | 1 | 0.89589 |
| Thyroid hormone synthesis | 13 | 1 | 0.83368 | 1 | 0.89589 |
| Propanoate Metabolism | 42 | 3 | 0.85037 | 1 | 0.90039 |
| Phenylalanine and Tyrosine Metabolism | 28 | 5 | 0.89061 | 1 | 0.92933 |
| Butyrate Metabolism | 19 | 3 | 0.91092 | 1 | 0.93695 |
| Vitamin K Metabolism | 14 | 1 | 0.98311 | 1 | 0.99363 |
| Oxidation of Branched Chain Fatty Acids | 26 | 4 | 0.99363 | 1 | 0.99363 |
