# Supplementary material for: Association of Metabolomic Biomarkers with Sleeve Gastrectomy Weight Loss Outcomes
Source: Metabolites. 2023 Mar 31;13(4):506. doi: 10.3390/metabo13040506 (PMC10145663; doi:10.3390/metabo13040506)
Supplement: Supplementary file 1 [file metabolites-13-00506-s001.zip › Supplementary Table 9.docx]

**Supplementary Table S9A:** Serum Data, Univariate Analysis results of only females for each variable/metabolite of Tertile 3 at three months post-sleeve gastrectomy compared with only female patients at baseline.

**Note: p-value is calculated with t-test as a default. p-value with (W) is calculated by the Wilcoxon Mann Whitney test.**

| Name | Mean (SD) of 3M | Mean (SD) of BL | p-value | q-value (FDR) | Fold Change | 3M/BL | p.value.origin |
| --- | --- | --- | --- | --- | --- | --- | --- |
| 1-Methylhistidine | 131.136 (56.030) | 137.443 (44.696) | 0.3458 (W) | NA | -1.05 | Down | 0.34578 |
| 2-Hydroxybutyric acid | 79.627 (83.040) | 37.911 (16.810) | 0.0071 (W) | NA | 2.1 | Up | 0.00712 |
| Acetic acid | 20.460 (10.724) | 24.374 (34.131) | 0.4708 (W) | NA | -1.19 | Down | 0.47083 |
| Betaine | 100.300 (249.253) | 31.514 (16.259) | 0.4551 (W) | NA | 3.18 | Up | 0.45514 |
| Acetoacetate | 152.891 (135.172) | 21.446 (17.040) | < 0.0001 (W) | NA | 7.13 | Up | 0.00007 |
| Carnitine | 45.036 (73.602) | 27.251 (10.801) | 0.3606 (W) | NA | 1.65 | Up | 0.36058 |
| Creatine | 16.927 (15.881) | 47.897 (25.102) | < 0.0001 (W) | NA | -2.83 | Down | 0.00004 |
| Citric acid | 187.164 (157.423) | 72.831 (26.733) | < 0.0001 (W) | NA | 2.57 | Up | 0.00002 |
| Choline | 9.258 (16.606) | 4.659 (2.875) | 0.8873 (W) | NA | 1.99 | Up | 0.88733 |
| Ethanol | 166.773 (275.019) | 67.537 (49.422) | 0.1421 (W) | NA | 2.47 | Up | 0.14213 |
| D-Glucose | 5115.391 (2850.459) | 4693.677 (1549.976) | 0.4923 (W) | NA | 1.09 | Up | 0.49234 |
| Glycerol | 408.064 (657.902) | 205.511 (143.199) | 0.1235 (W) | NA | 1.99 | Up | 0.12351 |
| Formate | 16.700 (3.442) | 36.683 (113.630) | 0.7671 (W) | NA | -2.2 | Down | 0.76708 |
| Hypoxanthine | 0.300 (0.533) | 0.227 (0.254) | 0.6616 (W) | NA | 1.32 | Up | 0.66157 |
| L-Alanine | 61.236 (27.514) | 86.603 (45.275) | 0.0670 (W) | NA | -1.41 | Down | 0.067 |
| Isoleucine | 59.964 (27.768) | 80.966 (43.136) | 0.1132 (W) | NA | -1.35 | Down | 0.11323 |
| L-Histidine | 7.953 (13.322) | 26.498 (25.389) | 0.0296 (W) | NA | -3.33 | Down | 0.0296 |
| L-Lactic acid | 1883.082 (854.254) | 2019.100 (1032.320) | 0.7033 (W) | NA | -1.07 | Down | 0.70333 |
| Pyruvic acid | 55.264 (25.377) | 80.249 (58.095) | 0.1934 (W) | NA | -1.45 | Down | 0.19342 |
| Succinate | 27.945 (6.398) | 23.711 (6.889) | 0.0777 | NA | 1.18 | Up | 0.07768 |
| Urea | 110.909 (94.368) | 137.260 (138.273) | 0.3201 (W) | NA | -1.24 | Down | 0.32009 |
| 3-Hydroxybutyric acid | 497.555 (433.597) | 43.497 (44.850) | < 0.0001 (W) | NA | 11.44 | Up | 0 |
| L-Arginine | 42.564 (74.742) | 18.560 (19.539) | 0.2400 (W) | NA | 2.29 | Up | 0.24003 |
| Creatinine | 57.945 (8.173) | 60.146 (18.516) | 0.5839 | NA | -1.04 | Down | 0.58389 |
| Malonate | 19.818 (47.768) | 9.646 (7.955) | 0.0369 (W) | NA | 2.05 | Up | 0.03693 |
| Isopropyl alcohol | 1.935 (1.700) | 3.817 (2.688) | 0.0147 (W) | NA | -1.97 | Down | 0.01472 |
| Acetone | 412.345 (1041.830) | 17.903 (11.479) | < 0.0001 (W) | NA | 23.03 | Up | 0.00001 |
| Isobutyric acid | 8.718 (4.831) | 10.014 (4.737) | 0.435 | NA | -1.15 | Down | 0.43496 |
| Methanol | 293.855 (27.944) | 333.983 (107.184) | 0.1439 (W) | NA | -1.14 | Down | 0.14389 |
| Propylene glycol | 1.118 (1.911) | 1.102 (1.908) | 0.3510 (W) | NA | 1.01 | Up | 0.35104 |
| Dimethyl sulfone | 10.682 (3.893) | 18.974 (61.264) | 0.1682 (W) | NA | -1.78 | Down | 0.16817 |
| C0 | 27.155 (7.071) | 33.986 (8.938) | 0.0255 | NA | -1.25 | Down | 0.02555 |
| C2 | 12.793 (4.689) | 7.072 (2.884) | 0.0001 (W) | NA | 1.81 | Up | 0.0001 |
| C3 | 0.202 (0.059) | 0.374 (0.152) | 0.0001 (W) | NA | -1.85 | Down | 0.00014 |
| C3-DC (C4-OH) | 0.125 (0.065) | 0.061 (0.021) | 0.0014 (W) | NA | 2.06 | Up | 0.0014 |
| C4 | 0.207 (0.194) | 0.207 (0.058) | 0.0198 (W) | NA | 1 | Up | 0.01976 |
| C5 | 0.206 (0.266) | 0.204 (0.080) | 0.0071 (W) | NA | 1.01 | Up | 0.00711 |
| C10 | 0.967 (2.152) | 0.253 (0.134) | 0.0259 (W) | NA | 3.82 | Up | 0.02589 |
| C14:1 | 0.165 (0.188) | 0.102 (0.026) | 0.3029 (W) | NA | 1.61 | Up | 0.30287 |
| C16 | 0.127 (0.084) | 0.076 (0.024) | 0.0018 (W) | NA | 1.66 | Up | 0.00183 |
| C18 | 2.205 (1.367) | 1.340 (1.340) | 0.1130 (W) | NA | 1.65 | Up | 0.11296 |
| C18:1 | 0.242 (0.193) | 0.141 (0.050) | 0.0052 (W) | NA | 1.72 | Up | 0.00519 |
| C18:2 | 0.115 (0.088) | 0.081 (0.028) | 0.1385 (W) | NA | 1.42 | Up | 0.13849 |
| Asn | 37.800 (4.569) | 47.389 (9.968) | < 0.0001 | NA | -1.25 | Down | 0.00008 |
| Asp | 10.037 (3.172) | 15.094 (4.637) | 0.0007 (W) | NA | -1.5 | Down | 0.00074 |
| Cit | 25.145 (7.016) | 29.709 (8.590) | 0.1171 | NA | -1.18 | Down | 0.1171 |
| Gln | 684.091 (85.172) | 660.171 (79.755) | 0.3977 | NA | 1.04 | Up | 0.39766 |
| Glu | 31.836 (9.665) | 73.411 (31.905) | < 0.0001 (W) | NA | -2.31 | Down | 0.00002 |
| Gly | 387.273 (69.233) | 315.857 (108.064) | 0.0100 (W) | NA | 1.23 | Up | 0.01002 |
| His | 83.045 (14.084) | 104.957 (14.872) | < 0.0001 | NA | -1.26 | Down | 0.00009 |
| Leu | 173.000 (46.024) | 281.657 (125.544) | 0.0012 (W) | NA | -1.63 | Down | 0.00123 |
| Lys | 261.455 (36.563) | 376.486 (80.721) | < 0.0001 | NA | -1.44 | Down | 0 |
| Met | 19.855 (4.908) | 33.697 (9.575) | < 0.0001 (W) | NA | -1.7 | Down | 0.00002 |
| Orn | 72.818 (22.794) | 106.197 (33.530) | 0.0036 | NA | -1.46 | Down | 0.00362 |
| L-Phenylalanine | 38.809 (8.751) | 53.631 (17.513) | 0.0007 | NA | -1.38 | Down | 0.00067 |
| Pro | 209.636 (83.857) | 296.143 (85.333) | 0.0024 (W) | NA | -1.41 | Down | 0.00237 |
| Thr | 133.864 (32.997) | 166.629 (39.669) | 0.0191 (W) | NA | -1.24 | Down | 0.01907 |
| Trp | 49.755 (10.518) | 82.129 (24.741) | < 0.0001 | NA | -1.65 | Down | 0 |
| Tyr | 69.473 (17.399) | 129.237 (42.652) | < 0.0001 (W) | NA | -1.86 | Down | 0.00001 |
| Val | 276.455 (53.824) | 422.086 (121.075) | < 0.0001 | NA | -1.53 | Down | 0 |
| Ac-Orn | 0.276 (0.224) | 0.552 (0.512) | 0.0958 (W) | NA | -2 | Down | 0.09583 |
| ADMA | 0.367 (0.161) | 0.484 (0.188) | 0.0077 (W) | NA | -1.32 | Down | 0.00767 |
| alpha-AAA | 0.516 (0.554) | 1.496 (1.216) | 0.0009 (W) | NA | -2.9 | Down | 0.00089 |
| Histamine | 0.245 (0.064) | 0.250 (0.050) | 0.3459 (W) | NA | -1.02 | Down | 0.34585 |
| Kynurenine | 1.563 (0.375) | 2.551 (0.577) | < 0.0001 (W) | NA | -1.63 | Down | 0.00001 |
| Met-SO | 0.159 (0.190) | 1.248 (2.161) | 0.0023 (W) | NA | -7.84 | Down | 0.00227 |
| Putrescine | 0.133 (0.031) | 0.135 (0.046) | 0.8916 | NA | -1.02 | Down | 0.89157 |
| Sarcosine | 0.870 (0.314) | 1.126 (0.381) | 0.0381 (W) | NA | -1.29 | Down | 0.03814 |
| SDMA | 0.547 (0.078) | 0.490 (0.129) | 0.0445 (W) | NA | 1.12 | Up | 0.04453 |
| Serotonin | 0.215 (0.247) | 0.444 (0.428) | 0.0694 (W) | NA | -2.07 | Down | 0.06939 |
| t4-OH-Pro | 15.364 (6.785) | 18.707 (13.623) | 0.8975 (W) | NA | -1.22 | Down | 0.89752 |
| Taurine | 67.064 (21.200) | 72.946 (30.296) | 0.8167 (W) | NA | -1.09 | Down | 0.81671 |
| lysoPC a C16:0 | 87.364 (26.271) | 93.186 (29.827) | 0.5651 | NA | -1.07 | Down | 0.56509 |
| lysoPC a C16:1 | 1.704 (0.779) | 2.352 (1.002) | 0.0562 | NA | -1.38 | Down | 0.05619 |
| lysoPC a C17:0 | 1.210 (0.383) | 1.449 (0.605) | 0.4026 (W) | NA | -1.2 | Down | 0.40258 |
| lysoPC a C18:0 | 17.438 (4.781) | 24.406 (8.448) | 0.0066 (W) | NA | -1.4 | Down | 0.00658 |
| lysoPC a C18:1 | 13.820 (5.418) | 14.070 (4.996) | 0.8878 | NA | -1.02 | Down | 0.88777 |
| lysoPC a C18:2 | 23.735 (11.932) | 27.426 (9.087) | 0.2822 | NA | -1.16 | Down | 0.28222 |
| lysoPC a C20:3 | 1.217 (0.498) | 2.019 (0.851) | 0.0026 (W) | NA | -1.66 | Down | 0.00258 |
| lysoPC a C20:4 | 7.433 (2.984) | 6.702 (2.387) | 0.5536 (W) | NA | 1.11 | Up | 0.55364 |
| lysoPC a C28:1 | 0.192 (0.092) | 0.270 (0.073) | 0.0056 | NA | -1.41 | Down | 0.00563 |
| PC aa C28:1 | 2.202 (0.853) | 2.747 (0.759) | 0.0494 | NA | -1.25 | Down | 0.04943 |
| PC aa C30:0 | 2.097 (0.742) | 2.617 (0.895) | 0.0882 | NA | -1.25 | Down | 0.0882 |
| PC aa C32:0 | 10.755 (3.395) | 9.274 (2.018) | 0.1945 | NA | 1.16 | Up | 0.19455 |
| PC aa C32:1 | 5.388 (2.537) | 8.453 (4.685) | 0.0446 (W) | NA | -1.57 | Down | 0.04457 |
| PC aa C32:2 | 1.445 (1.113) | 3.096 (1.351) | 0.0003 (W) | NA | -2.14 | Down | 0.00033 |
| PC aa C32:3 | 0.357 (0.146) | 0.357 (0.096) | 0.995 | NA | -1 | Down | 0.99499 |
| PC aa C34:1 | 122.918 (42.841) | 128.326 (40.693) | 0.8367 (W) | NA | -1.04 | Down | 0.83673 |
| PC aa C34:2 | 195.909 (29.382) | 217.314 (34.597) | 0.0393 (W) | NA | -1.11 | Down | 0.03932 |
| PC aa C34:3 | 6.177 (2.803) | 10.341 (3.136) | 0.0006 (W) | NA | -1.67 | Down | 0.00061 |
| PC aa C34:4 | 0.513 (0.296) | 1.112 (0.421) | 0.0003 (W) | NA | -2.17 | Down | 0.00031 |
| PC aa C36:0 | 1.464 (0.545) | 1.434 (0.649) | 0.8889 | NA | 1.02 | Up | 0.88886 |
| PC aa C36:1 | 23.555 (8.766) | 35.054 (9.944) | 0.0010 (W) | NA | -1.49 | Down | 0.00098 |
| PC aa C36:2 | 129.873 (37.855) | 194.429 (35.979) | < 0.0001 | NA | -1.5 | Down | 0.00001 |
| PC aa C36:3 | 62.236 (26.250) | 92.920 (24.897) | 0.0024 (W) | NA | -1.49 | Down | 0.00237 |
| PC aa C36:4 | 150.364 (36.947) | 144.580 (32.778) | 0.6227 | NA | 1.04 | Up | 0.62274 |
| PC aa C36:5 | 8.170 (4.817) | 13.574 (5.046) | 0.0035 (W) | NA | -1.66 | Down | 0.00347 |
| PC aa C36:6 | 0.282 (0.196) | 0.573 (0.193) | 0.0007 (W) | NA | -2.03 | Down | 0.00074 |
| PC aa C38:0 | 1.780 (0.434) | 2.039 (0.596) | 0.2793 (W) | NA | -1.15 | Down | 0.27933 |
| PC aa C38:3 | 22.209 (7.078) | 38.591 (11.836) | < 0.0001 (W) | NA | -1.74 | Down | 0.00005 |
| PC aa C38:4 | 74.645 (17.781) | 92.474 (22.076) | 0.0125 (W) | NA | -1.24 | Down | 0.01247 |
| PC aa C38:5 | 30.936 (10.803) | 34.071 (9.112) | 0.3461 | NA | -1.1 | Down | 0.34607 |
| PC aa C38:6 | 48.709 (13.938) | 45.817 (14.588) | 0.3674 (W) | NA | 1.06 | Up | 0.36739 |
| PC aa C40:1 | 0.254 (0.031) | 0.266 (0.047) | 0.4405 | NA | -1.05 | Down | 0.44052 |
| PC aa C40:4 | 1.743 (0.385) | 2.396 (0.735) | 0.0100 (W) | NA | -1.38 | Down | 0.01001 |
| PC aa C40:5 | 5.705 (1.360) | 7.644 (2.523) | 0.0025 | NA | -1.34 | Down | 0.00252 |
| PC aa C40:6 | 14.664 (3.053) | 19.068 (5.628) | 0.0022 | NA | -1.3 | Down | 0.00221 |
| PC aa C42:0 | 0.354 (0.109) | 0.398 (0.141) | 0.358 | NA | -1.12 | Down | 0.35796 |
| PC aa C42:1 | 0.177 (0.044) | 0.183 (0.068) | 0.7743 | NA | -1.04 | Down | 0.77433 |
| PC aa C42:5 | 0.190 (0.050) | 0.243 (0.074) | 0.0503 (W) | NA | -1.28 | Down | 0.05027 |
| PC aa C42:6 | 0.225 (0.042) | 0.296 (0.080) | 0.0006 | NA | -1.31 | Down | 0.00058 |
| PC ae C30:0 | 0.148 (0.044) | 0.189 (0.055) | 0.0332 | NA | -1.27 | Down | 0.0332 |
| PC ae C30:1 | 0.094 (0.036) | 0.086 (0.035) | 0.4934 | NA | 1.1 | Up | 0.49339 |
| PC ae C32:1 | 1.961 (0.517) | 1.682 (0.397) | 0.0657 | NA | 1.17 | Up | 0.0657 |
| PC ae C32:2 | 0.539 (0.192) | 0.459 (0.123) | 0.2163 | NA | 1.17 | Up | 0.21633 |
| PC ae C34:0 | 0.646 (0.202) | 0.766 (0.207) | 0.098 | NA | -1.19 | Down | 0.09799 |
| PC ae C34:1 | 5.305 (1.548) | 5.480 (1.407) | 0.7266 | NA | -1.03 | Down | 0.72662 |
| PC ae C34:2 | 6.885 (2.040) | 7.853 (1.813) | 0.1406 | NA | -1.14 | Down | 0.14059 |
| PC ae C34:3 | 6.126 (1.479) | 5.379 (1.117) | 0.0806 | NA | 1.14 | Up | 0.08059 |
| PC ae C36:0 | 0.389 (0.121) | 0.388 (0.093) | 0.9815 | NA | 1 | Up | 0.98149 |
| PC ae C36:1 | 5.047 (1.551) | 6.462 (1.661) | 0.0162 | NA | -1.28 | Down | 0.01619 |
| PC ae C36:2 | 7.300 (2.242) | 9.123 (2.366) | 0.0291 | NA | -1.25 | Down | 0.02911 |
| PC ae C36:3 | 3.768 (1.143) | 4.818 (1.128) | 0.0102 | NA | -1.28 | Down | 0.01025 |
| PC ae C36:4 | 13.206 (3.498) | 14.291 (3.296) | 0.353 | NA | -1.08 | Down | 0.35301 |
| PC ae C36:5 | 10.532 (3.113) | 9.153 (2.020) | 0.0918 | NA | 1.15 | Up | 0.09177 |
| PC ae C38:0 | 1.164 (0.477) | 1.585 (0.426) | 0.0139 (W) | NA | -1.36 | Down | 0.0139 |
| PC ae C38:1 | 0.978 (0.507) | 1.098 (0.698) | 0.5030 (W) | NA | -1.12 | Down | 0.50304 |
| PC ae C38:2 | 1.431 (0.761) | 2.072 (0.680) | 0.0112 | NA | -1.45 | Down | 0.01119 |
| PC ae C38:3 | 3.226 (0.934) | 4.416 (1.194) | 0.0042 | NA | -1.37 | Down | 0.00419 |
| PC ae C38:4 | 8.669 (1.850) | 9.614 (2.023) | 0.1754 | NA | -1.11 | Down | 0.17538 |
| PC ae C38:5 | 12.503 (3.614) | 11.722 (2.466) | 0.4193 | NA | 1.07 | Up | 0.41929 |
| PC ae C38:6 | 5.430 (1.892) | 5.213 (1.216) | 0.655 | NA | 1.04 | Up | 0.65504 |
| PC ae C40:1 | 0.768 (0.294) | 0.895 (0.230) | 0.1416 | NA | -1.17 | Down | 0.1416 |
| PC ae C40:2 | 1.548 (0.479) | 1.639 (0.482) | 0.5864 | NA | -1.06 | Down | 0.58641 |
| PC ae C40:3 | 1.500 (0.447) | 1.711 (0.433) | 0.1691 | NA | -1.14 | Down | 0.16906 |
| PC ae C40:4 | 1.733 (0.342) | 2.069 (0.420) | 0.0178 (W) | NA | -1.19 | Down | 0.01775 |
| PC ae C40:5 | 3.394 (0.882) | 3.212 (0.724) | 0.495 | NA | 1.06 | Up | 0.49498 |
| PC ae C40:6 | 2.805 (0.660) | 2.956 (0.715) | 0.5372 | NA | -1.05 | Down | 0.53719 |
| PC ae C42:1 | 0.214 (0.065) | 0.227 (0.067) | 0.4025 (W) | NA | -1.06 | Down | 0.40247 |
| PC ae C42:2 | 0.273 (0.079) | 0.336 (0.084) | 0.0332 | NA | -1.23 | Down | 0.03315 |
| PC ae C42:3 | 0.440 (0.144) | 0.544 (0.141) | 0.0379 | NA | -1.24 | Down | 0.0379 |
| PC ae C42:4 | 0.627 (0.190) | 0.793 (0.216) | 0.0277 | NA | -1.26 | Down | 0.0277 |
| PC ae C42:5 | 1.980 (0.558) | 1.886 (0.465) | 0.4099 (W) | NA | 1.05 | Up | 0.40987 |
| PC ae C44:3 | 0.047 (0.014) | 0.065 (0.021) | 0.0204 (W) | NA | -1.38 | Down | 0.02042 |
| PC ae C44:4 | 0.224 (0.064) | 0.274 (0.071) | 0.0441 | NA | -1.22 | Down | 0.04406 |
| PC ae C44:5 | 1.308 (0.508) | 1.255 (0.414) | 0.7272 | NA | 1.04 | Up | 0.72723 |
| PC ae C44:6 | 0.893 (0.261) | 0.978 (0.292) | 0.3923 | NA | -1.1 | Down | 0.39226 |
| SM (OH) C14:1 | 5.926 (1.895) | 5.726 (1.771) | 0.7486 | NA | 1.04 | Up | 0.74862 |
| SM (OH) C16:1 | 3.343 (1.011) | 2.963 (0.954) | 0.2627 | NA | 1.13 | Up | 0.26268 |
| SM (OH) C22:1 | 9.145 (2.958) | 12.783 (2.952) | 0.0009 | NA | -1.4 | Down | 0.00089 |
| SM (OH) C22:2 | 9.310 (2.135) | 10.068 (2.760) | 0.4093 | NA | -1.08 | Down | 0.40929 |
| SM (OH) C24:1 | 0.945 (0.328) | 1.160 (0.282) | 0.0395 | NA | -1.23 | Down | 0.03952 |
| SM C16:0 | 116.264 (35.028) | 99.477 (21.334) | 0.1575 | NA | 1.17 | Up | 0.15754 |
| SM C16:1 | 20.573 (6.162) | 17.691 (4.144) | 0.0818 | NA | 1.16 | Up | 0.08177 |
| SM C18:0 | 30.409 (9.732) | 23.137 (6.252) | 0.0054 | NA | 1.31 | Up | 0.00543 |
| SM C18:1 | 16.439 (5.126) | 11.971 (3.447) | 0.0018 | NA | 1.37 | Up | 0.00181 |
| SM C20:2 | 0.528 (0.266) | 0.379 (0.129) | 0.1162 (W) | NA | 1.39 | Up | 0.11619 |
| SM C24:0 | 15.897 (5.836) | 18.363 (3.591) | 0.209 | NA | -1.16 | Down | 0.20905 |
| SM C24:1 | 56.518 (19.194) | 43.971 (11.184) | 0.0613 | NA | 1.29 | Up | 0.06129 |
| SM C26:0 | 0.101 (0.026) | 0.100 (0.028) | 0.9353 | NA | 1.01 | Up | 0.93532 |
| SM C26:1 | 0.257 (0.082) | 0.198 (0.072) | 0.0284 | NA | 1.29 | Up | 0.02842 |
| H1 | 6239.364 (3447.100) | 7642.086 (2568.570) | 0.0534 (W) | NA | -1.22 | Down | 0.05343 |

**Supplementary Table S9B:** Fecal Data, Univariate Analysis results of only females for each variable/metabolite of Tertile 3 at three months post-sleeve gastrectomy compared with only female patients at baseline.

**Note: p-value is calculated with t-test as a default. p-value with (W) is calculated by the Wilcoxon Mann Whitney test.**

| Name | Mean (SD) of 3M | Mean (SD) of BL | p-value | q-value (FDR) | Fold Change | 3M/BL | p.value.origin |
| --- | --- | --- | --- | --- | --- | --- | --- |
| 2-Oxoglutarate | 32.073 (63.574) | 17.891 (12.807) | 0.2244 (W) | NA | 1.79 | Up | 0.22439 |
| 3-Phenylpropionate | 42.289 (78.513) | 32.359 (20.037) | 0.0435 (W) | NA | 1.31 | Up | 0.04353 |
| 4-Hydroxyphenylacetate | 5.825 (4.108) | 10.721 (7.771) | 0.0026 (W) | NA | -1.84 | Down | 0.00255 |
| 4-Hydroxyphenyllactate | 9.572 (8.907) | 22.081 (31.378) | 0.0178 (W) | NA | -2.31 | Down | 0.01785 |
| Arabinose | 48.259 (36.818) | 73.549 (61.436) | 0.1597 (W) | NA | -1.52 | Down | 0.15971 |
| Butyrate | 438.583 (323.673) | 891.714 (602.753) | 0.0165 (W) | NA | -2.03 | Down | 0.01647 |
| Caprylate | 50.829 (59.033) | 91.792 (218.345) | 0.2983 (W) | NA | -1.81 | Down | 0.29828 |
| Creatine | 33.701 (24.650) | 56.646 (30.529) | 0.0011 (W) | NA | -1.68 | Down | 0.00108 |
| Hypoxanthine | 42.674 (25.821) | 53.697 (31.526) | 0.3179 | NA | -1.26 | Down | 0.31787 |
| Methylamine | 23.835 (13.581) | 23.727 (10.225) | 0.6524 (W) | NA | 1 | Up | 0.65236 |
| N6-Acetyllysine | 29.437 (30.668) | 28.092 (24.799) | 0.2142 (W) | NA | 1.05 | Up | 0.21415 |
| N-Acetylcysteine | 17.387 (15.629) | 23.525 (10.416) | 0.0048 (W) | NA | -1.35 | Down | 0.00476 |
| N-Acetylglutamate | 12.385 (9.202) | 20.417 (14.427) | 0.0028 (W) | NA | -1.65 | Down | 0.00279 |
| Nicotinate | 12.403 (9.039) | 21.012 (12.686) | 0.0477 (W) | NA | -1.69 | Down | 0.04771 |
| p-Cresol | 78.587 (77.585) | 67.193 (47.141) | 0.7954 (W) | NA | 1.17 | Up | 0.79535 |
| Phenylacetate | 55.223 (26.312) | 87.029 (46.992) | 0.0475 | NA | -1.58 | Down | 0.04754 |
| Propionate | 925.153 (707.280) | 1405.189 (691.500) | 0.0879 (W) | NA | -1.52 | Down | 0.08794 |
| Thymine | 36.924 (17.156) | 48.422 (20.899) | 0.1193 | NA | -1.31 | Down | 0.11928 |
| Trimethylamine | 15.615 (17.813) | 10.580 (8.626) | 0.8378 (W) | NA | 1.48 | Up | 0.83776 |
| Uracil | 99.706 (58.695) | 137.136 (60.528) | 0.0898 | NA | -1.38 | Down | 0.08981 |
| Urocanate | 15.126 (10.802) | 14.418 (7.809) | 0.8403 (W) | NA | 1.05 | Up | 0.84031 |
| Valerate | 189.280 (183.326) | 239.020 (158.566) | 0.1885 (W) | NA | -1.26 | Down | 0.18855 |
| ?-Methylhistidine | 10.351 (13.586) | 14.737 (10.041) | 0.0353 (W) | NA | -1.42 | Down | 0.03527 |
| Acetic acid | 756.445 (514.899) | 1408.066 (496.644) | 0.0007 | NA | -1.86 | Down | 0.00075 |
| Betaine | 13.930 (27.180) | 10.203 (13.899) | 0.1794 (W) | NA | 1.37 | Up | 0.17942 |
| Acetoacetate | 7.469 (5.612) | 18.309 (36.956) | 0.0853 (W) | NA | -2.45 | Down | 0.08533 |
| Creatinine | 13.971 (12.550) | 46.156 (92.993) | 0.0881 (W) | NA | -3.3 | Down | 0.08812 |
| Dimethylamine | 7.628 (14.988) | 5.681 (6.137) | 0.4863 (W) | NA | 1.34 | Up | 0.48629 |
| Choline | 2.910 (3.265) | 4.903 (5.022) | 0.0209 (W) | NA | -1.69 | Down | 0.02091 |
| Ethanol | 70.405 (74.851) | 113.242 (248.627) | 0.5629 (W) | NA | -1.61 | Down | 0.5629 |
| D-Glucose | 267.209 (246.071) | 403.179 (361.172) | 0.2618 (W) | NA | -1.51 | Down | 0.26175 |
| Glycine | 196.072 (121.937) | 292.591 (118.908) | 0.0295 | NA | -1.49 | Down | 0.02951 |
| Fumaric acid | 18.380 (13.348) | 22.585 (14.004) | 0.4863 (W) | NA | -1.23 | Down | 0.48631 |
| Formate | 27.451 (18.708) | 35.077 (18.677) | 0.1461 (W) | NA | -1.28 | Down | 0.14609 |
| D-Galactose | 32.112 (17.842) | 77.119 (184.954) | 0.1855 (W) | NA | -2.4 | Down | 0.18546 |
| L-Glutamic acid | 520.787 (234.469) | 917.337 (461.121) | 0.0009 | NA | -1.76 | Down | 0.00089 |
| Tyrosine | 29.757 (11.796) | 46.229 (22.126) | 0.0107 (W) | NA | -1.55 | Down | 0.01069 |
| L-Phenylalanine | 75.535 (34.519) | 130.551 (51.592) | 0.0011 (W) | NA | -1.73 | Down | 0.00108 |
| Ala | 620.374 (267.866) | 1176.252 (547.960) | 0.0007 (W) | NA | -1.9 | Down | 0.00067 |
| Pro | 178.863 (84.254) | 269.967 (131.494) | 0.0451 | NA | -1.51 | Down | 0.04511 |
| Methylamine.1 | 16.943 (9.116) | 25.696 (26.378) | 0.1164 (W) | NA | -1.52 | Down | 0.11645 |
| Thr | 153.173 (70.830) | 225.548 (102.938) | 0.0436 | NA | -1.47 | Down | 0.04365 |
| Asp | 113.339 (94.403) | 133.123 (95.495) | 0.4923 (W) | NA | -1.17 | Down | 0.49229 |
| Isoleucine | 92.139 (35.386) | 158.372 (68.886) | 0.0017 (W) | NA | -1.72 | Down | 0.00169 |
| L-Fucose | 18.418 (17.164) | 28.338 (58.848) | 0.2244 (W) | NA | -1.54 | Down | 0.22438 |
| His | 11.019 (11.791) | 23.730 (29.512) | 0.1678 (W) | NA | -2.15 | Down | 0.16785 |
| L-Lysine | 130.465 (84.853) | 231.848 (194.123) | 0.0420 (W) | NA | -1.78 | Down | 0.04195 |
| Ser | 134.159 (74.636) | 263.492 (211.000) | 0.0466 (W) | NA | -1.96 | Down | 0.04661 |
| L-Lactic acid | 45.721 (52.983) | 47.316 (28.969) | 0.1385 (W) | NA | -1.03 | Down | 0.13853 |
| Aspartate | 94.597 (34.308) | 170.924 (96.542) | 0.0209 (W) | NA | -1.81 | Down | 0.02091 |
| Phenylacetate.1 | 2.324 (1.045) | 4.420 (2.947) | 0.0107 (W) | NA | -1.9 | Down | 0.01069 |
| Pyruvic acid | 6.843 (6.081) | 8.577 (5.736) | 0.1944 (W) | NA | -1.25 | Down | 0.19441 |
| Taurine | 6.411 (10.209) | 57.963 (95.413) | 0.0041 (W) | NA | -9.04 | Down | 0.00414 |
| Succinate | 61.105 (65.487) | 272.287 (772.048) | 0.5999 (W) | NA | -4.46 | Down | 0.59989 |
| Xanthine | 1.399 (1.331) | 5.988 (27.154) | 0.6524 (W) | NA | -4.28 | Down | 0.65235 |
| Uracil.1 | 58.548 (36.629) | 79.376 (35.146) | 0.1087 | NA | -1.36 | Down | 0.10872 |
| 3-Hydroxybutyric acid | 9.078 (7.172) | 11.224 (13.492) | 0.7535 (W) | NA | -1.24 | Down | 0.75354 |
| L-Arginine | 8.944 (7.739) | 79.926 (238.367) | 0.4863 (W) | NA | -8.94 | Down | 0.48626 |
| Creatinine.1 | 139.633 (103.034) | 182.631 (133.563) | 0.4050 (W) | NA | -1.31 | Down | 0.40499 |
| L-Leucine | 37.652 (17.703) | 227.128 (673.563) | 0.1597 (W) | NA | -6.03 | Down | 0.15971 |
| Malonate | 45.170 (21.703) | 85.241 (36.130) | 0.0018 | NA | -1.89 | Down | 0.00182 |
| Methionine | 85.098 (34.959) | 128.980 (80.492) | 0.0171 | NA | -1.52 | Down | 0.01707 |
| Isovaleric acid | 0.879 (1.412) | 6.074 (14.965) | 0.0172 (W) | NA | -6.91 | Down | 0.01723 |
| 3-Hydroxyisovaleric acid | 11.609 (15.087) | 24.288 (56.016) | 0.5211 (W) | NA | -2.09 | Down | 0.52114 |
| Isopropyl alcohol | 176.455 (99.009) | 271.703 (109.730) | 0.0069 (W) | NA | -1.54 | Down | 0.00694 |
| Valine | 10.377 (17.312) | 20.133 (61.641) | 0.2404 (W) | NA | -1.94 | Down | 0.24039 |
| Trimethylamine.1 | 16.429 (7.845) | 29.376 (21.437) | 0.0353 (W) | NA | -1.79 | Down | 0.03527 |
| Acetone | 211.470 (122.000) | 320.967 (164.152) | 0.0572 | NA | -1.52 | Down | 0.05721 |
| Isobutyric acid | 666.422 (119.101) | 1051.923 (1649.285) | 0.0145 (W) | NA | -1.58 | Down | 0.01454 |
| Methanol | 0.337 (0.227) | 30.472 (168.917) | 0.0019 (W) | NA | -90.51 | Down | 0.00186 |
| C0 | 2.577 (0.782) | 3.895 (2.184) | 0.0533 (W) | NA | -1.51 | Down | 0.05329 |
| C2 | 0.055 (0.018) | 0.072 (0.033) | 0.0991 (W) | NA | -1.31 | Down | 0.09912 |
| C3 | 0.020 (0.013) | 0.030 (0.015) | 0.1552 (W) | NA | -1.49 | Down | 0.15523 |
| C3-DC (C4-OH) | 0.092 (0.059) | 0.138 (0.088) | 0.1176 (W) | NA | -1.49 | Down | 0.11758 |
| C3-OH | 0.012 (0.005) | 0.018 (0.006) | 0.0081 | NA | -1.46 | Down | 0.00807 |
| C3:1 | 0.013 (0.008) | 0.018 (0.009) | 0.3748 (W) | NA | -1.33 | Down | 0.37477 |
| C4 | 0.028 (0.009) | 0.039 (0.032) | 0.1621 (W) | NA | -1.38 | Down | 0.16213 |
| C4:1 | 0.049 (0.016) | 0.049 (0.014) | 0.7985 (W) | NA | 1.01 | Up | 0.79852 |
| C5 | 0.033 (0.011) | 0.046 (0.044) | 0.2736 (W) | NA | -1.38 | Down | 0.27358 |
| C5-DC (C6-OH) | 0.026 (0.014) | 0.033 (0.010) | 0.0499 (W) | NA | -1.27 | Down | 0.04986 |
| C5-M-DC | 0.024 (0.005) | 0.034 (0.010) | 0.0002 | NA | -1.4 | Down | 0.00023 |
| C5-OH (C3-DC-M) | 0.034 (0.013) | 0.074 (0.061) | 0.0048 (W) | NA | -2.17 | Down | 0.00476 |
| C5:1 | 0.031 (0.010) | 0.038 (0.032) | 0.5999 (W) | NA | -1.21 | Down | 0.59989 |
| C5:1-DC | 0.060 (0.019) | 0.097 (0.035) | 0.0006 (W) | NA | -1.61 | Down | 0.00059 |
| C6 (C4:1-DC) | 0.035 (0.011) | 0.045 (0.012) | 0.0162 | NA | -1.29 | Down | 0.01615 |
| C6:1 | 0.028 (0.005) | 0.035 (0.008) | 0.015 | NA | -1.25 | Down | 0.01497 |
| C7-DC | 0.017 (0.004) | 0.035 (0.044) | 0.0209 (W) | NA | -2.05 | Down | 0.02091 |
| C8 | 0.062 (0.009) | 0.065 (0.011) | 0.3524 | NA | -1.06 | Down | 0.35244 |
| C9 | 0.020 (0.006) | 0.031 (0.027) | 0.0607 (W) | NA | -1.59 | Down | 0.06073 |
| C10 | 0.048 (0.009) | 0.058 (0.019) | 0.0533 (W) | NA | -1.21 | Down | 0.05329 |
| C10:1 | 0.313 (0.078) | 0.321 (0.099) | 0.7961 | NA | -1.03 | Down | 0.79615 |
| C10:2 | 0.037 (0.010) | 0.053 (0.037) | 0.0379 (W) | NA | -1.45 | Down | 0.03787 |
| C12 | 0.055 (0.016) | 0.064 (0.027) | 0.1706 (W) | NA | -1.16 | Down | 0.17062 |
| C12-DC | 0.067 (0.017) | 0.076 (0.023) | 0.3807 (W) | NA | -1.12 | Down | 0.38073 |
| C12:1 | 0.414 (0.148) | 0.397 (0.143) | 0.7447 | NA | 1.04 | Up | 0.74472 |
| C14 | 0.031 (0.012) | 0.044 (0.025) | 0.1461 (W) | NA | -1.41 | Down | 0.14609 |
| C14:1 | 0.011 (0.003) | 0.014 (0.009) | 0.2736 (W) | NA | -1.35 | Down | 0.27358 |
| C14:1-OH | 0.030 (0.015) | 0.030 (0.017) | 0.7778 (W) | NA | -1.03 | Down | 0.77782 |
| C14:2 | 0.017 (0.008) | 0.020 (0.012) | 0.4110 (W) | NA | -1.16 | Down | 0.41096 |
| C14:2-OH | 0.013 (0.004) | 0.018 (0.007) | 0.0298 | NA | -1.41 | Down | 0.02979 |
| C16 | 0.067 (0.048) | 0.117 (0.151) | 0.4754 (W) | NA | -1.73 | Down | 0.47538 |
| C16-OH | 0.192 (0.188) | 0.160 (0.183) | 0.5629 (W) | NA | 1.2 | Up | 0.5629 |
| C16:1 | 0.022 (0.012) | 0.034 (0.026) | 0.0881 (W) | NA | -1.52 | Down | 0.08812 |
| C16:1-OH | 0.223 (0.215) | 0.184 (0.194) | 0.5813 (W) | NA | 1.21 | Up | 0.58126 |
| C16:2 | 0.014 (0.005) | 0.022 (0.019) | 0.0569 (W) | NA | -1.54 | Down | 0.05691 |
| C16:2-OH | 0.017 (0.005) | 0.025 (0.011) | 0.0048 (W) | NA | -1.52 | Down | 0.00476 |
| C18 | 0.074 (0.052) | 0.151 (0.173) | 0.0332 (W) | NA | -2.04 | Down | 0.03321 |
| C18:1 | 0.042 (0.027) | 0.076 (0.078) | 0.1794 (W) | NA | -1.82 | Down | 0.17942 |
| C18:1-OH | 0.018 (0.007) | 0.032 (0.018) | 0.0069 (W) | NA | -1.74 | Down | 0.00694 |
| C18:2 | 0.020 (0.011) | 0.033 (0.031) | 0.3807 (W) | NA | -1.67 | Down | 0.38073 |
| Cit | 112.658 (64.092) | 252.314 (139.870) | < 0.0001 | NA | -2.24 | Down | 0.00008 |
| Gln | 48.029 (30.977) | 93.090 (94.787) | 0.1900 (W) | NA | -1.94 | Down | 0.19003 |
| Glu | 731.172 (381.226) | 1119.131 (542.377) | 0.0407 | NA | -1.53 | Down | 0.04074 |
| Gly | 317.033 (129.699) | 549.186 (274.650) | 0.0083 (W) | NA | -1.73 | Down | 0.00832 |
| Orn | 60.698 (67.078) | 67.575 (55.685) | 0.4860 (W) | NA | -1.11 | Down | 0.48603 |
| Phe | 162.489 (118.655) | 297.545 (189.113) | 0.0217 (W) | NA | -1.83 | Down | 0.02166 |
| Trp | 26.963 (13.038) | 45.179 (25.475) | 0.0406 (W) | NA | -1.68 | Down | 0.04062 |
| Tyr | 159.900 (70.230) | 292.310 (175.249) | 0.001 | NA | -1.83 | Down | 0.00097 |
| Ac-Orn | 462.659 (1446.060) | 517.402 (1419.108) | 0.4863 (W) | NA | -1.12 | Down | 0.48626 |
| alpha-AAA | 10.851 (16.323) | 17.216 (67.786) | 0.7728 (W) | NA | -1.59 | Down | 0.77277 |
| Met-SO | 10.487 (7.422) | 25.937 (23.428) | 0.0732 (W) | NA | -2.47 | Down | 0.07321 |
| Putrescine | 11.202 (16.209) | 14.419 (20.532) | 0.4542 (W) | NA | -1.29 | Down | 0.4542 |
| Sarcosine | 137.274 (334.310) | 153.178 (287.212) | 0.0140 (W) | NA | -1.12 | Down | 0.01397 |
| SDMA | 0.202 (0.448) | 1.209 (4.122) | 0.0178 (W) | NA | -5.97 | Down | 0.01785 |
| Serotonin | 0.571 (0.801) | 0.457 (0.536) | 0.8366 (W) | NA | 1.25 | Up | 0.83659 |
| Spermidine | 5.097 (5.221) | 65.291 (120.810) | 0.0043 (W) | NA | -12.81 | Down | 0.00432 |
| t4-OH-Pro | 0.396 (0.518) | 3.217 (3.859) | 0.0009 (W) | NA | -8.13 | Down | 0.00089 |
| lysoPC a C14:0 | 4.087 (0.237) | 3.881 (0.288) | 0.045 | NA | 1.05 | Up | 0.04504 |
| lysoPC a C16:0 | 0.848 (0.421) | 2.171 (3.267) | 0.0830 (W) | NA | -2.56 | Down | 0.08298 |
| lysoPC a C16:1 | 0.052 (0.013) | 0.082 (0.042) | 0.0091 (W) | NA | -1.57 | Down | 0.0091 |
| lysoPC a C17:0 | 0.092 (0.031) | 0.128 (0.077) | 0.0569 (W) | NA | -1.39 | Down | 0.05691 |
| lysoPC a C18:0 | 0.644 (0.085) | 1.249 (1.844) | 0.0569 (W) | NA | -1.94 | Down | 0.05691 |
| lysoPC a C18:1 | 0.280 (0.402) | 0.608 (0.952) | 0.0193 (W) | NA | -2.17 | Down | 0.01933 |
| lysoPC a C18:2 | 0.356 (0.492) | 0.814 (1.928) | 0.0881 (W) | NA | -2.29 | Down | 0.08812 |
| lysoPC a C20:3 | 0.115 (0.035) | 0.116 (0.025) | 0.6188 (W) | NA | -1.01 | Down | 0.61878 |
| lysoPC a C20:4 | 0.038 (0.014) | 0.059 (0.069) | 0.2995 (W) | NA | -1.55 | Down | 0.29952 |
| lysoPC a C24:0 | 0.093 (0.016) | 0.094 (0.012) | 0.801 | NA | -1.01 | Down | 0.80103 |
| lysoPC a C26:0 | 0.112 (0.014) | 0.119 (0.013) | 0.1595 | NA | -1.06 | Down | 0.15954 |
| lysoPC a C26:1 | 0.029 (0.008) | 0.029 (0.010) | 0.7535 (W) | NA | 1.02 | Up | 0.75352 |
| lysoPC a C28:0 | 0.118 (0.013) | 0.129 (0.015) | 0.0392 | NA | -1.09 | Down | 0.03918 |
| lysoPC a C28:1 | 0.045 (0.006) | 0.048 (0.012) | 0.5813 (W) | NA | -1.06 | Down | 0.58126 |
| PC aa C24:0 | 0.035 (0.004) | 0.038 (0.006) | 0.1594 | NA | -1.08 | Down | 0.1594 |
| PC aa C26:0 | 0.222 (0.016) | 0.214 (0.017) | 0.2338 | NA | 1.03 | Up | 0.23379 |
| PC aa C28:1 | 0.020 (0.008) | 0.025 (0.006) | 0.2736 (W) | NA | -1.22 | Down | 0.27358 |
| PC aa C30:0 | 0.071 (0.025) | 0.096 (0.055) | 0.2858 (W) | NA | -1.34 | Down | 0.28575 |
| PC aa C32:0 | 0.185 (0.044) | 0.207 (0.083) | 0.1855 (W) | NA | -1.12 | Down | 0.18546 |
| PC aa C32:1 | 0.020 (0.012) | 0.034 (0.026) | 0.0466 (W) | NA | -1.69 | Down | 0.04661 |
| PC aa C32:2 | 0.023 (0.008) | 0.027 (0.012) | 0.2042 (W) | NA | -1.19 | Down | 0.20419 |
| PC aa C32:3 | 0.033 (0.004) | 0.036 (0.004) | 0.0271 | NA | -1.11 | Down | 0.02708 |
| PC aa C34:1 | 0.151 (0.114) | 0.373 (0.428) | 0.0328 (W) | NA | -2.47 | Down | 0.03282 |
| PC aa C34:2 | 0.102 (0.104) | 0.302 (0.658) | 0.0881 (W) | NA | -2.96 | Down | 0.08812 |
| PC aa C34:3 | 0.018 (0.005) | 0.023 (0.030) | 0.6967 (W) | NA | -1.29 | Down | 0.69669 |
| PC aa C34:4 | 0.022 (0.005) | 0.024 (0.004) | 0.1706 (W) | NA | -1.08 | Down | 0.17062 |
| PC aa C36:0 | 0.171 (0.022) | 0.173 (0.024) | 0.9679 (W) | NA | -1.01 | Down | 0.96786 |
| PC aa C36:1 | 0.035 (0.007) | 0.076 (0.074) | 0.0466 (W) | NA | -2.2 | Down | 0.04661 |
| PC aa C36:2 | 0.065 (0.040) | 0.216 (0.626) | 0.0781 (W) | NA | -3.32 | Down | 0.07809 |
| PC aa C36:3 | 0.070 (0.090) | 0.227 (0.801) | 0.0193 (W) | NA | -3.25 | Down | 0.01933 |
| PC aa C36:4 | 0.061 (0.081) | 0.360 (1.631) | 0.2983 (W) | NA | -5.86 | Down | 0.29828 |
| PC aa C36:5 | 0.021 (0.004) | 0.026 (0.024) | 0.9893 (W) | NA | -1.22 | Down | 0.98928 |
| PC aa C36:6 | 0.016 (0.002) | 0.018 (0.010) | 0.9893 (W) | NA | -1.15 | Down | 0.98928 |
| PC aa C38:0 | 0.035 (0.005) | 0.039 (0.007) | 0.1885 (W) | NA | -1.11 | Down | 0.18855 |
| PC aa C38:3 | 0.210 (0.028) | 0.218 (0.023) | 0.3309 | NA | -1.04 | Down | 0.33086 |
| PC aa C38:4 | 0.050 (0.006) | 0.051 (0.008) | 0.7957 | NA | -1.01 | Down | 0.79566 |
| PC aa C38:5 | 0.028 (0.005) | 0.036 (0.025) | 0.0935 (W) | NA | -1.3 | Down | 0.0935 |
| PC aa C38:6 | 0.030 (0.003) | 0.031 (0.006) | 0.2284 | NA | -1.06 | Down | 0.22843 |
| PC aa C40:1 | 0.103 (0.013) | 0.095 (0.015) | 0.1621 (W) | NA | 1.08 | Up | 0.16213 |
| PC aa C40:2 | 0.083 (0.008) | 0.088 (0.011) | 0.1643 | NA | -1.07 | Down | 0.1643 |
| PC aa C40:3 | 0.177 (0.019) | 0.191 (0.021) | 0.062 | NA | -1.08 | Down | 0.062 |
| PC aa C40:4 | 0.057 (0.006) | 0.059 (0.007) | 0.5101 | NA | -1.03 | Down | 0.5101 |
| PC aa C40:5 | 0.024 (0.005) | 0.026 (0.007) | 0.4110 (W) | NA | -1.09 | Down | 0.41096 |
| PC aa C40:6 | 0.101 (0.009) | 0.099 (0.013) | 0.8119 | NA | 1.01 | Up | 0.81191 |
| PC aa C42:0 | 0.058 (0.008) | 0.060 (0.008) | 0.3084 | NA | -1.05 | Down | 0.30843 |
| PC aa C42:1 | 0.039 (0.006) | 0.041 (0.006) | 0.4654 | NA | -1.04 | Down | 0.4654 |
| PC aa C42:2 | 0.037 (0.005) | 0.038 (0.008) | 0.9679 (W) | NA | -1.03 | Down | 0.96786 |
| PC aa C42:4 | 0.044 (0.009) | 0.044 (0.008) | 0.7964 | NA | 1.02 | Up | 0.79643 |
| PC aa C42:5 | 0.038 (0.006) | 0.045 (0.008) | 0.0353 (W) | NA | -1.17 | Down | 0.03527 |
| PC aa C42:6 | 0.041 (0.016) | 0.045 (0.013) | 0.4983 | NA | -1.08 | Down | 0.49834 |
| PC ae C30:0 | 0.028 (0.004) | 0.034 (0.010) | 0.0569 (W) | NA | -1.18 | Down | 0.05691 |
| PC ae C30:1 | 0.008 (0.002) | 0.010 (0.004) | 0.1888 | NA | -1.24 | Down | 0.18879 |
| PC ae C30:2 | 0.015 (0.003) | 0.017 (0.003) | 0.2736 (W) | NA | -1.09 | Down | 0.27358 |
| PC ae C32:1 | 0.021 (0.005) | 0.023 (0.014) | 0.6379 (W) | NA | -1.12 | Down | 0.63791 |
| PC ae C32:2 | 0.026 (0.004) | 0.028 (0.005) | 0.1980 (W) | NA | -1.09 | Down | 0.198 |
| PC ae C34:0 | 0.045 (0.006) | 0.047 (0.008) | 0.3688 | NA | -1.06 | Down | 0.36882 |
| PC ae C34:1 | 0.033 (0.010) | 0.060 (0.066) | 0.1885 (W) | NA | -1.8 | Down | 0.18855 |
| PC ae C34:2 | 0.031 (0.004) | 0.066 (0.074) | 0.0781 (W) | NA | -2.12 | Down | 0.07809 |
| PC ae C34:3 | 0.031 (0.004) | 0.041 (0.023) | 0.1243 (W) | NA | -1.31 | Down | 0.12428 |
| PC ae C36:0 | 0.036 (0.006) | 0.038 (0.006) | 0.2983 (W) | NA | -1.06 | Down | 0.29828 |
| PC ae C36:1 | 0.081 (0.013) | 0.101 (0.036) | 0.0533 (W) | NA | -1.25 | Down | 0.05329 |
| PC ae C36:2 | 0.027 (0.004) | 0.037 (0.016) | 0.0305 (W) | NA | -1.37 | Down | 0.03052 |
| PC ae C36:3 | 0.036 (0.005) | 0.046 (0.019) | 0.0091 (W) | NA | -1.28 | Down | 0.0091 |
| PC ae C36:4 | 0.059 (0.009) | 0.070 (0.024) | 0.1794 (W) | NA | -1.18 | Down | 0.17942 |
| PC ae C36:5 | 0.028 (0.004) | 0.033 (0.007) | 0.0140 (W) | NA | -1.18 | Down | 0.01397 |
| PC ae C38:0 | 0.022 (0.002) | 0.024 (0.004) | 0.2406 | NA | -1.07 | Down | 0.24058 |
| PC ae C38:1 | 0.103 (0.014) | 0.102 (0.020) | 0.8806 (W) | NA | 1.01 | Up | 0.88064 |
| PC ae C38:2 | 0.046 (0.011) | 0.049 (0.018) | 0.4588 (W) | NA | -1.08 | Down | 0.45879 |
| PC ae C38:3 | 0.076 (0.013) | 0.086 (0.021) | 0.1229 (W) | NA | -1.13 | Down | 0.12294 |
| PC ae C38:4 | 0.044 (0.007) | 0.046 (0.006) | 0.4804 | NA | -1.03 | Down | 0.48044 |
| PC ae C38:5 | 0.045 (0.006) | 0.047 (0.009) | 0.7778 (W) | NA | -1.04 | Down | 0.77782 |
| PC ae C38:6 | 0.018 (0.004) | 0.020 (0.004) | 0.1313 (W) | NA | -1.14 | Down | 0.13126 |
| PC ae C40:1 | 0.045 (0.005) | 0.048 (0.007) | 0.2446 | NA | -1.07 | Down | 0.24463 |
| PC ae C40:2 | 0.064 (0.010) | 0.068 (0.009) | 0.2997 | NA | -1.05 | Down | 0.29973 |
| PC ae C40:3 | 0.091 (0.009) | 0.097 (0.015) | 0.2297 | NA | -1.07 | Down | 0.22974 |
| PC ae C40:4 | 0.034 (0.005) | 0.038 (0.008) | 0.0991 (W) | NA | -1.13 | Down | 0.09912 |
| PC ae C40:5 | 0.055 (0.006) | 0.057 (0.008) | 0.3371 | NA | -1.05 | Down | 0.33705 |
| PC ae C40:6 | 0.033 (0.006) | 0.033 (0.005) | 0.5448 (W) | NA | -1.01 | Down | 0.54481 |
| PC ae C42:0 | 0.219 (0.018) | 0.222 (0.015) | 0.5448 (W) | NA | -1.01 | Down | 0.54481 |
| PC ae C42:1 | 0.041 (0.006) | 0.043 (0.008) | 0.4445 | NA | -1.05 | Down | 0.4445 |
| PC ae C42:2 | 0.041 (0.007) | 0.043 (0.006) | 0.4110 (W) | NA | -1.03 | Down | 0.41096 |
| PC ae C42:3 | 0.049 (0.007) | 0.050 (0.007) | 0.6757 | NA | -1.02 | Down | 0.67568 |
| PC ae C42:5 | 0.270 (0.011) | 0.277 (0.023) | 0.241 | NA | -1.02 | Down | 0.24096 |
| PC ae C44:3 | 0.036 (0.005) | 0.037 (0.008) | 0.8614 (W) | NA | -1.03 | Down | 0.86137 |
| PC ae C44:4 | 0.044 (0.005) | 0.047 (0.007) | 0.4425 (W) | NA | -1.06 | Down | 0.44252 |
| PC ae C44:5 | 0.030 (0.006) | 0.031 (0.006) | 0.8614 (W) | NA | -1.02 | Down | 0.86137 |
| PC ae C44:6 | 0.038 (0.006) | 0.041 (0.006) | 0.2858 | NA | -1.06 | Down | 0.28576 |
| SM (OH) C14:1 | 0.015 (0.007) | 0.023 (0.032) | 0.8825 (W) | NA | -1.5 | Down | 0.88254 |
| SM (OH) C16:1 | 0.015 (0.009) | 0.029 (0.047) | 0.7327 (W) | NA | -1.97 | Down | 0.73274 |
| SM (OH) C22:1 | 0.018 (0.006) | 0.033 (0.070) | 0.2179 (W) | NA | -1.89 | Down | 0.21789 |
| SM (OH) C22:2 | 0.017 (0.009) | 0.016 (0.012) | 0.5035 (W) | NA | 1.08 | Up | 0.50352 |
| SM (OH) C24:1 | 0.013 (0.008) | 0.012 (0.006) | 0.6711 | NA | 1.08 | Up | 0.6711 |
| SM C16:0 | 0.309 (0.216) | 0.762 (1.344) | 0.5813 (W) | NA | -2.47 | Down | 0.58126 |
| SM C16:1 | 0.041 (0.006) | 0.050 (0.024) | 0.2736 (W) | NA | -1.22 | Down | 0.27358 |
| SM C18:0 | 0.061 (0.054) | 0.193 (0.331) | 0.5999 (W) | NA | -3.14 | Down | 0.59989 |
| SM C18:1 | 0.010 (0.008) | 0.021 (0.039) | 0.6621 (W) | NA | -1.97 | Down | 0.66213 |
| SM C20:2 | 0.083 (0.009) | 0.085 (0.014) | 0.6369 | NA | -1.03 | Down | 0.63686 |
| SM C24:0 | 0.074 (0.007) | 0.093 (0.079) | 0.2572 (W) | NA | -1.26 | Down | 0.2572 |
| SM C26:0 | 0.010 (0.005) | 0.011 (0.006) | 0.7613 | NA | -1.06 | Down | 0.76131 |
