# Supplementary figures and images for: Association of Metabolomic Biomarkers with Sleeve Gastrectomy Weight Loss Outcomes
Source: Metabolites. 2023 Mar 31;13(4):506. doi: 10.3390/metabo13040506 (PMC10145663; doi:10.3390/metabo13040506)

A

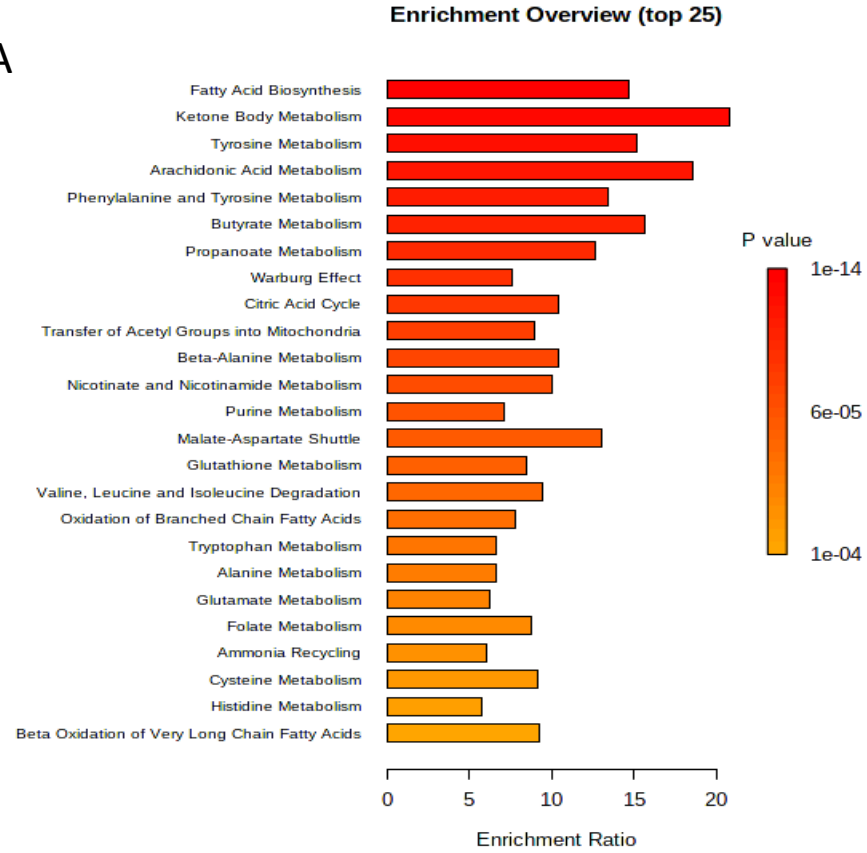

B

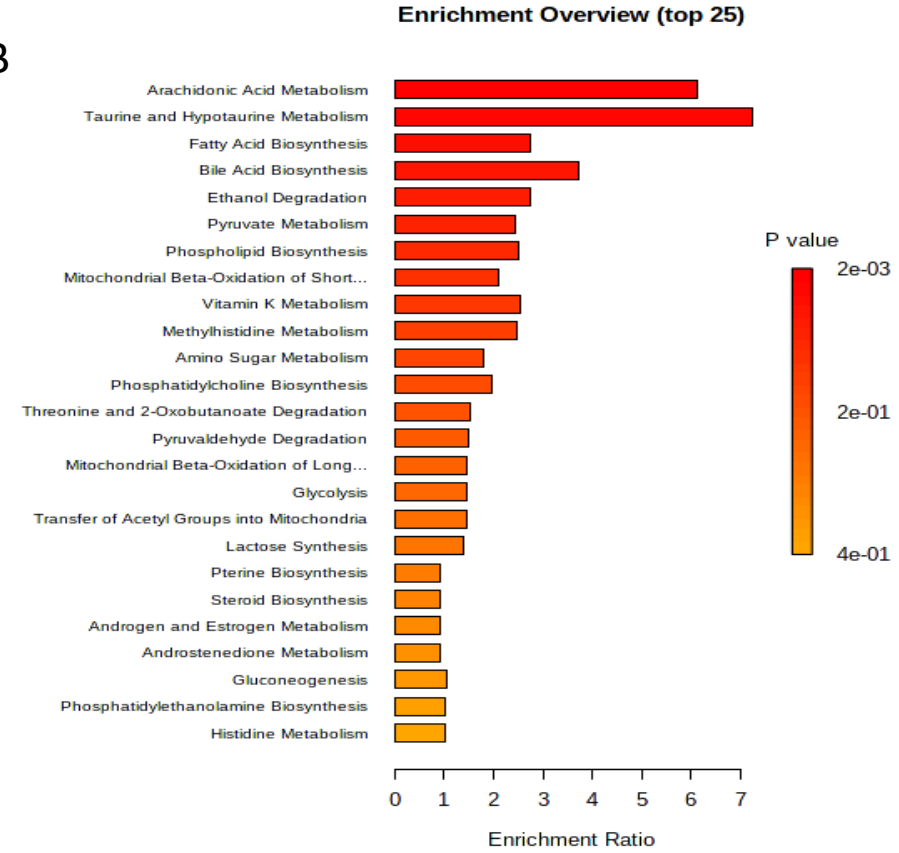

Supplement: Supplementary file 1 [file metabolites-13-00506-s001.zip › 1-Figure 1.pdf]

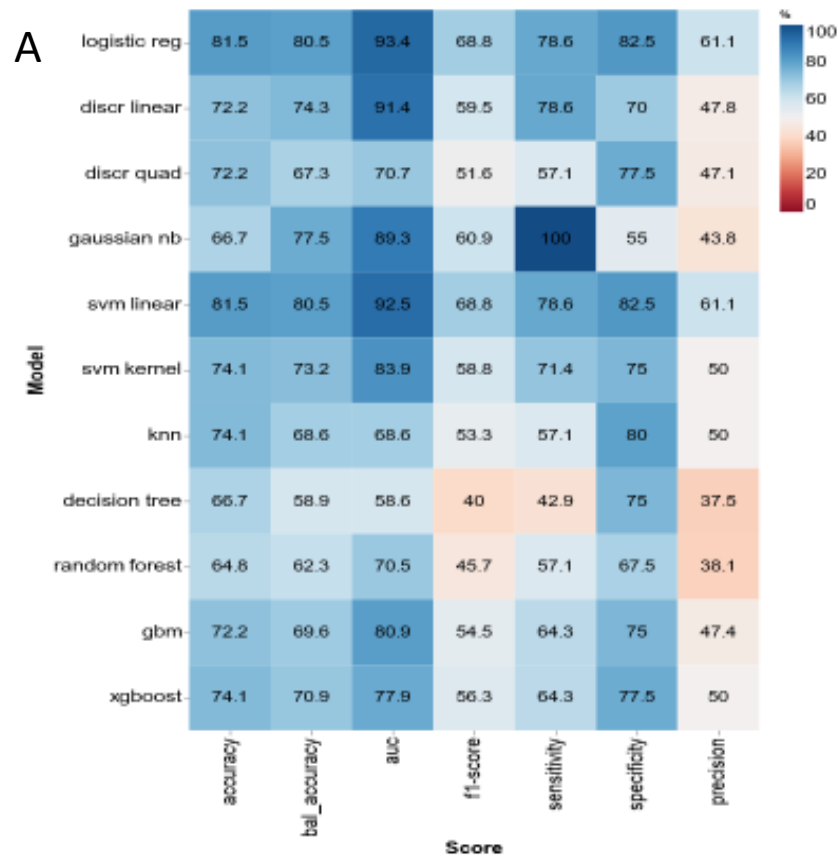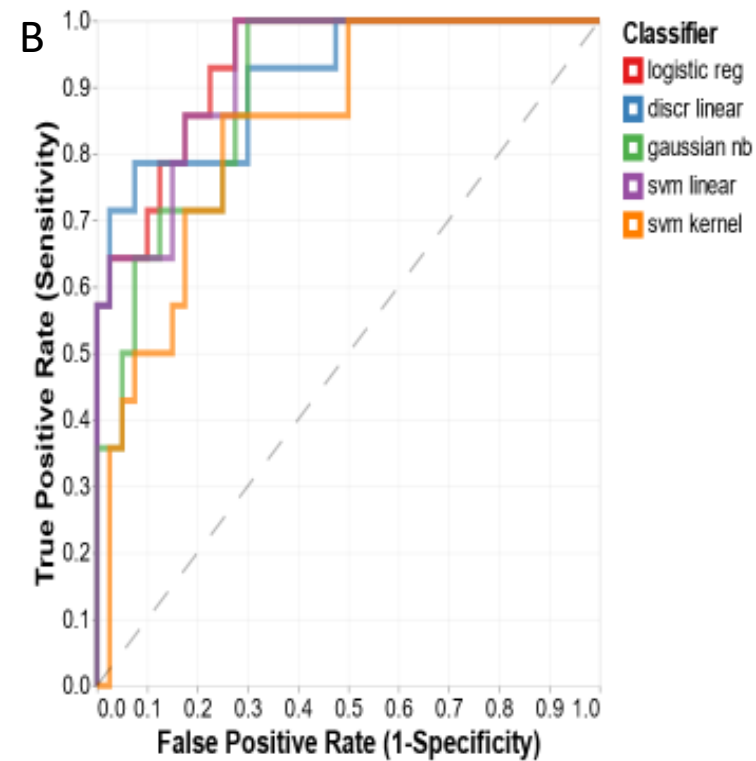

Supplement: Supplementary file 1 [file metabolites-13-00506-s001.zip › 3-Figure 3.pdf]
